# Catalytic asymmetric allylation of aldehydes with alkenes through allylic C(sp^3^)–H functionalization mediated by organophotoredox and chiral chromium hybrid catalysis†
†Electronic supplementary information (ESI) available. See DOI: 10.1039/c8sc05677c


**DOI:** 10.1039/c8sc05677c

**Published:** 2019-01-17

**Authors:** Harunobu Mitsunuma, Shun Tanabe, Hiromu Fuse, Kei Ohkubo, Motomu Kanai

**Affiliations:** a Graduate School of Pharmaceutical Sciences , The University of Tokyo , 7-3-1 Bunkyo-ku , Tokyo 113-0033 , Japan . Email: h-mitsunuma@mol.f.u-tokyo.ac.jp ; Email: kanai@mol.f.u-tokyo.ac.jp; b Institute for Advanced Co-Creation Studies , Open and Transdisciplinary Research Initiatives , Osaka University , Osaka 565-0871 , Japan

## Abstract

We describe a hybrid system that realizes cooperativity between an organophotoredox acridinium catalyst and a chiral chromium complex catalyst, thereby enabling unprecedented exploitation of unactivated hydrocarbon alkenes as precursors to chiral allylchromium nucleophiles for asymmetric allylation of aldehydes. The reaction proceeds under visible light irradiation at room temperature, affording the corresponding homoallylic alcohols with a diastereomeric ratio >20/1 and up to 99% ee. The addition of Mg(ClO_4_)_2_ markedly enhanced both the reactivity and enantioselectivity.

## Abstract 2

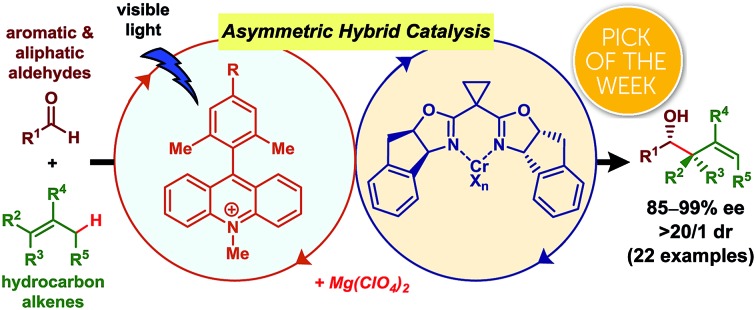
We have developed a catalytic asymmetric nucleophilic allylation of aldehydes using simple alkenes as pronucleophiles without relying on stoichiometric metals.

## Introduction

**Fig. 1:**
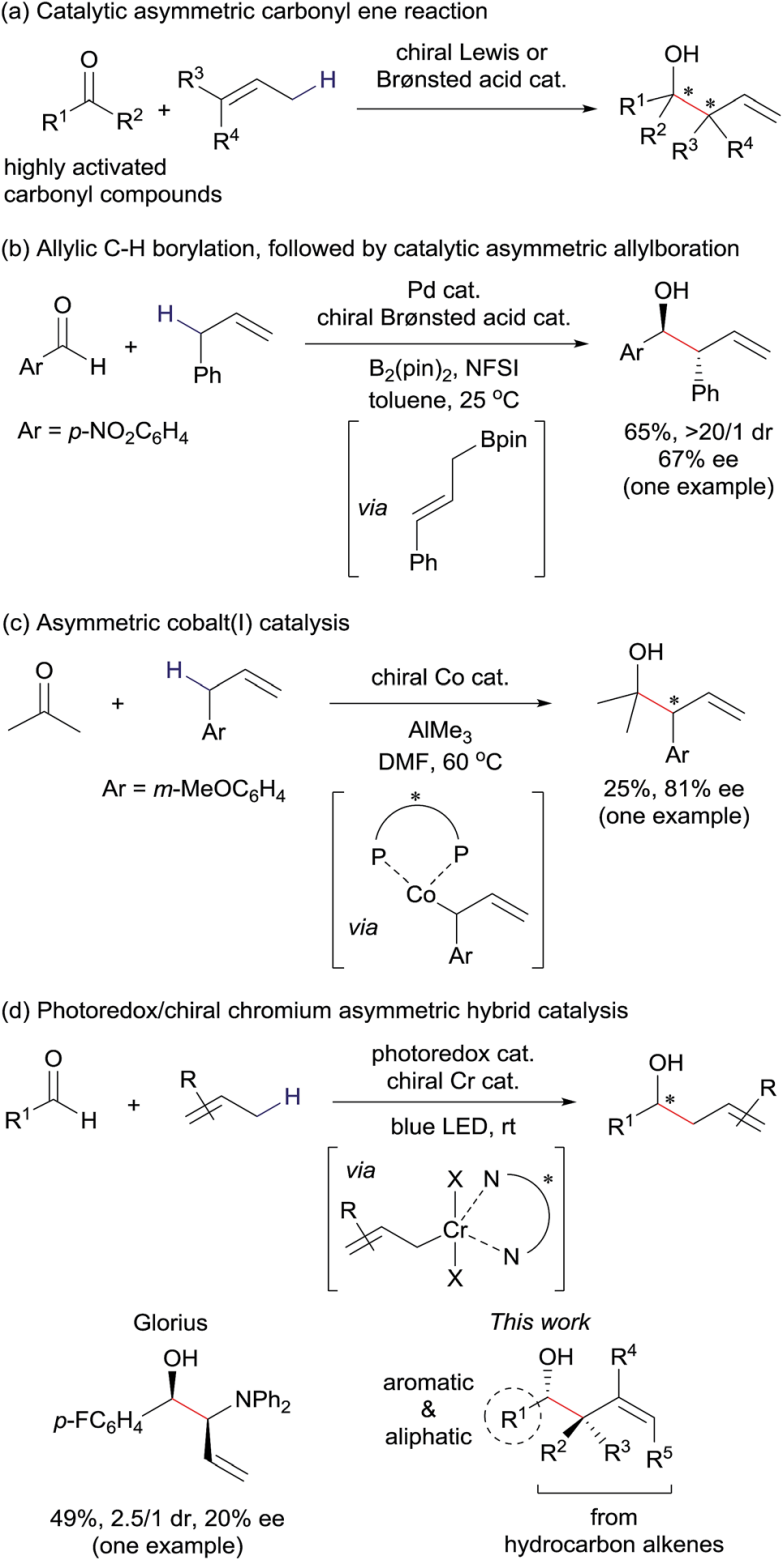
Methods for catalytic asymmetric allylation of carbonyl compounds through C(sp^3^)–H functionalization of unactivated alkenes as pronucleophiles. (a) Chiral Lewis acid or Brønsted acid-catalyzed carbonyl ene reactions.^3^ (b) One-pot, stepwise generation of allylboronate followed by chiral Brønsted acid-catalyzed allylboration.^5^ (c) Through chiral allylcobalt species.^6^ (d) Through chiral allylchromium species by Glorius^7^ and our group (this work).

Catalytic asymmetric C(sp^3^)–H bond functionalization is an emerging synthetic method affording direct access to useful chiral building blocks from stable organic molecules.^1^ For example, catalytic asymmetric allylation of aldehydes using unactivated alkenes as pronucleophiles produces enantiomerically-enriched homoallylic alcohols, which act as versatile synthetic intermediates for numerous functional molecules, including various drug leads.^2^ The catalytic asymmetric carbonyl ene reaction is a representative example (Fig. 1(a)).^3^ The electrophile scope of catalytic asymmetric carbonyl ene reactions, however, is limited to highly reactive aldehydes or ketones, such as glyoxylic esters, formaldehyde, fluoral, or ketoesters. In an effort to expand the substrate scope, we envisioned that a reaction mechanism dissected by chiral nucleophilic allylmetal species, which are generated from alkenes *via in situ* allylic C(sp^3^)–H bond activation, would be feasible.^4^ Some previous examples related to this strategy are reported. Gong presented a one-pot procedure for asymmetric nucleophilic allylation of an aldehyde with an alkene involving palladium-catalyzed allylic C(sp^3^)–H borylation of the alkene to generate an allylboronate and chiral phosphoric acid-catalyzed asymmetric allylation of the aldehyde with the thus-generated allylboronate (Fig. 1(b)).^5^ Mita and Sato reported a cobalt-catalyzed enantioselective allylation between acetone and an allylarene *via* nucleophilic chiral allylcobalt(i) species generated through oxidative addition of an allylic C(sp^3^)–H bond to a low valent cobalt complex (Fig. 1(c)).^6^ During our study, Glorius' group reported an asymmetric allylation between an aldehyde and an allylamine mediated by combining an iridium photoredox catalyst and a chiral chromium catalyst (Fig. 1(d)).^7^ Considering the attractive feature of this reaction to generate versatile chiral homoallylic alcohols in a single operation from aldehydes and alkenes, further investigation of this reaction type are highly desirable.^8^,^9^

Photocatalyzed C(sp^3^)–H bond activation followed by oxidative interception of the resulting carbon-centered radical by a metal complex catalyst is a groundbreaking concept for generating organometallic intermediates from substrates traditionally considered inert.^10^–^13^ Application of organometallic intermediates generated by this method, however, has mainly been limited to cross-coupling reactions. Extension of the chemistry to facilitate the addition of these nucleophiles to polar moieties, such as carbonyl groups, has yet to be explored, except for the recent example by Glorius using electron-rich aromatic- or amine-substituted alkenes.^7^ Herein we report an asymmetric hybrid catalyst system comprising an organophotoredox catalyst and a chiral chromium complex catalyst, which enables asymmetric allylation of aldehydes by nucleophilic chiral allylchromium species generated *in situ* from hydrocarbon feedstock alkenes by C(sp^3^)–H bond activation (Fig. 1(d)).

## Results and discussion

### Optimization of reaction conditions

**Fig. 2:**
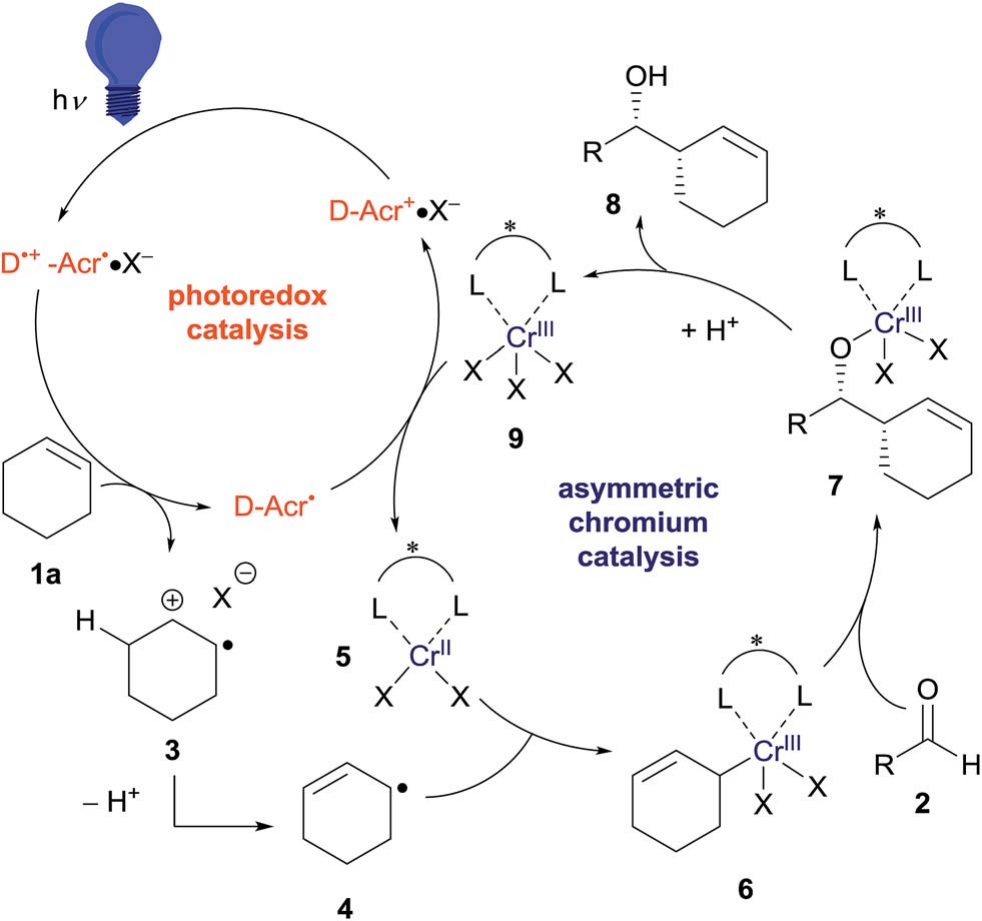
Proposed catalytic cycle.

Our mechanistic rationale for this transformation is illustrated in Fig. 2. Based on an earlier report by the Wu laboratory,^14^ allyl radical **4** should be accessible from alkene **1a***via* electron-transfer oxidation of the π-bond by a photoexcited electron-donor substituted acridinium catalyst (D˙^+^–Acr˙; D = 2,6-xylyl or mesityl) to generate radical cation **3**, followed by deprotonation. A reduced form of the chiral chromium(ii) catalyst **5** would then intercept the thus-formed allyl radical **4** to give chiral allyl chromium(iii) complex **6**. We anticipated that this species would react with aldehydes **2***via* a six-membered chair transition state to produce enantiomerically-enriched chromium alkoxide **7** in a *syn*-selective manner. Protonolysis of **7** would then afford the target homoallylic alcohol **8** and an oxidized chromium(iii) complex **9**. Finally, electron-transfer reduction of **9** by the reduced form of the photocatalyst (D–Acr˙) would regenerate **5** and the oxidized form of the photocatalyst (D–Acr^+^), thus closing the catalytic cycle.^15^

Based on this hypothesis, we began optimizing the reaction conditions using benzaldehyde (**2a**) and cyclohexene (**1a**: 20 equiv.) as model substrates, and a combination of 5 mol% CrCl_2_ and 2.5 mol% acridinium photoredox catalysts (2,6-Xyl–Acr^+^·ClO_4_^–^; **10**),^16^ under 430 nm visible light irradiation at room temperature (Table 1). As expected, the desired reaction did not proceed at all in the absence of the chromium complex (entry 1). In the presence of CrCl_2_, however, **8a** was obtained in 36% yield with an excellent diastereomeric ratio (dr) of >20/1 (entry 2). Encouraged by this finding, we then screened various chiral ligands for the chromium catalysts that were previously shown to be effective for asymmetric Nozaki–Hiyama–Kishi reactions (entries 3–6).^17^ The chiral catalysts strongly retarded the reaction, however, with only **L1** (ref. 18) affording **8a** with diminished yield (12%) and low enantioselectivity (20% ee). Through extensive screening of other chiral ligands, we identified an indane-BOX ligand (**L5**)^19^ that effectively induced good enantioselectivity (74% ee), although the yield of **8a** remained unsatisfactory (8%, entry 7).

**Table 1:** Optimization of the reaction conditions^*a*^

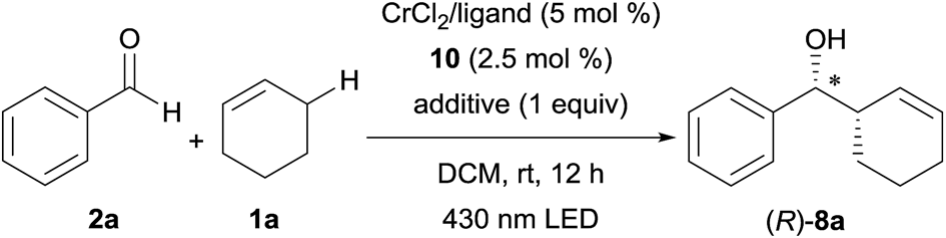					
Entry	Ligand	Additive	Yield (%)	dr	ee (%)
1^*b*^	None	None	0	n.d.	n.d.
2	None	None	36	>20/1	n.d.
3	**L1**	None	12	>20/1	20
4^*c*^	**L2**	None	0	n.d.	n.d.
5^*d*^	**L3**	None	0	n.d.	n.d.
6^*d*^	**L4**	None	0	n.d.	n.d.
7	**L5**	None	8	>20/1	74
8	**L5**	LiBF_4_	40	>20/1	63
9	**L5**	LiClO_4_	44	>20/1	99
10	**L5**	NaClO_4_	14	>20/1	99
11	**L5**	Ca(ClO_4_)_2_·*x*H_2_O	8	>20/1	98
12	**L5**	Mg(ClO_4_)_2_	68	>20/1	99
13^*e*^	**L5**	Mg(ClO_4_)_2_	63	>20/1	99
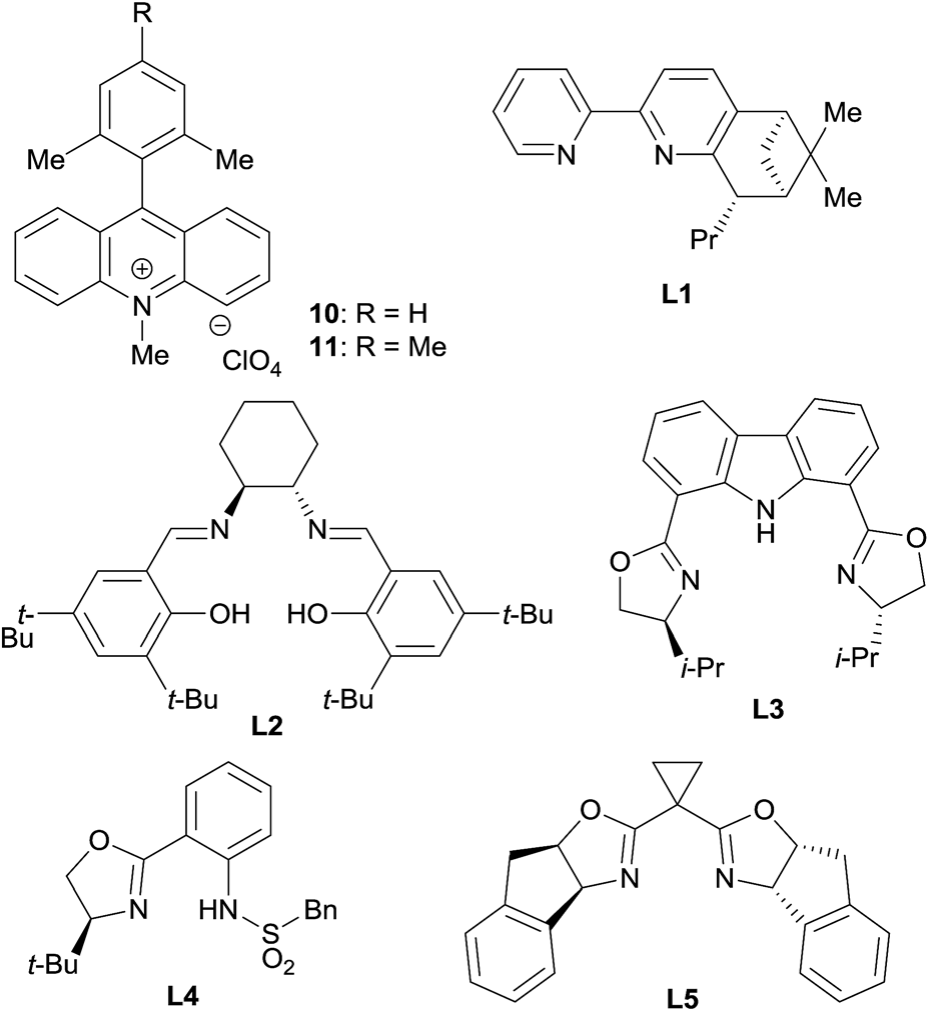					

^*a*^General reaction conditions: **2a** (0.25 mmol), **1a** (5.0 mmol), CrCl_2_ (0.0125 mmol), ligand (0.0125 mmol), **10** (0.00625 mmol), and additive (0.25 mmol) were reacted in dichloromethane (DCM; 2.5 mL) at room temperature under 430 nm LED irradiation for 12 h. Yield and diastereomeric ratio were determined by ^1^H NMR analysis of the crude mixture using 1,1,2,2-tetrachloroethane as an internal standard. The enantioselectivity of **8a** was determined by chiral stationary HPLC analysis after isolation. n.d. = not determined.

^*b*^Without CrCl_2_.

^*c*^10 mol% Et_3_N was added.

^*d*^5 mol% Et_3_N was added.

^*e*^Mes–Acr^+^·ClO_4_^–^**11** was used as a photocatalyst.

We supposed that the low reactivity was due to the high oxidation potential of **1a**. Due to the small oxidation potential difference between substrate **1a** and photoredox catalyst **10** (see ESI†), only low concentration of cation radical **3** were generated. To improve the reactivity, we investigated the effects of salt additives to stabilize cation radical **3**, which would accelerate the overall reaction rate.^20^ Screening of several electrolytes revealed that adding LiBF_4_ dramatically enhanced the reactivity; **8a** was obtained in 40% yield with 63% ee (entry 8). Moreover, the use of LiClO_4_ increased the enantioselectivity up to 99% (entry 9). Further exploration of alkali and alkali-earth metal perchlorates (entries 10–12) identified Mg(ClO_4_)_2_ as the optimal additive; **8a** was obtained in 68% yield with >20/1 dr and 99% ee (entry 12). Additionally, the use of photocatalyst **11**, bearing a mesityl group instead of a xylyl group, did not negatively affect these results (entry 13). It is noteworthy that compared to traditional catalytic asymmetric carbonyl allylations,^2^ except for the Krische's method,2i,p this reaction can bypass the preactivation step of the nucleophile using stoichiometric metal species.

### Substrate scope

Under these optimized conditions, we next evaluated the substrate scope (Table 2). The reaction of cyclohexene (**1a**) with substituted benzaldehydes afforded products **8a–8g** with almost complete diastereo- and enantioselectivity (up to >20/1 dr, 99% ee). The reaction well tolerated aryl halide moieties (**8b–8d**), and proceeded chemoselectively at the aldehyde functional group in the presence of a ketone (**8e**) or an ester (**8f**) functional group. The method was also easily extended to other cyclic alkenes, with both cyclopentene (**1b**) and cycloheptene (**1c**) reacting with excellent stereoselectivity (**8h–8k**).

**Table 2:** Substrate scope of catalytic asymmetric allylation^*a*^

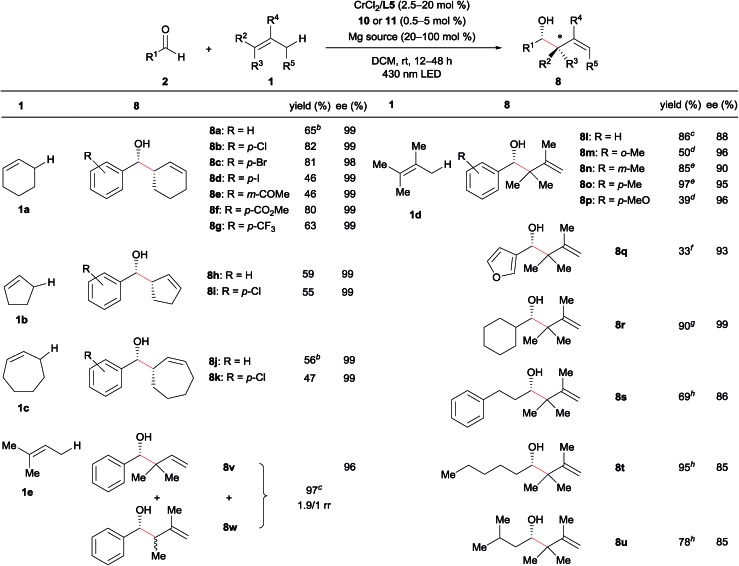

^*a*^General reaction conditions: aldehyde **2** (0.25 mmol), alkene **1** (5.0 mmol), CrCl_2_ (0.0125 mmol), **L5** (0.0125 mmol), **10** (0.00625 mmol), and Mg(ClO_4_)_2_ (0.25 mmol) were reacted in DCM (2.5 mL) at room temperature under 430 nm LED irradiation for 12 h. Yield is isolated yield. The diastereomeric ratio was >20/1 in each case (**8a–8k**), as determined by ^1^H NMR analysis of the crude mixture. The enantioselectivity was determined by chiral stationary HPLC analysis after isolation.

^*b*^CrCl_2_ (10 mol%) and **L5** (10 mol%) were used.

^*c*^Alkene (2 equiv.), CrCl_2_ (2.5 mol%), **L5** (2.5 mol%), **11** (0.5 mol%), Mg(ClO_4_)_2_ (1 equiv), and DCM (0.125 M) were used.

^*d*^Alkene (20 equiv.), CrCl_3_·3THF (10 mol%), NaO*t*-Bu (30 mol%), **L5** (10 mol%), **11** (1.25 mol%), Mg(ClO_4_)_2_ (1 equiv.), and DCM (0.0625 M) were used.

^*e*^Alkene (5 equiv.), CrCl_2_ (10 mol%), **L5** (10 mol%), **11** (1.25 mol%), Mg(ClO_4_)_2_ (1 equiv.), and DCM (0.0625 M) were used.

^*f*^Alkene (20 equiv.), CrCl_2_ (20 mol%), **L5** (20 mol%), **11** (5 mol%), Mg(ClO_4_)_2_ (1 equiv.), and DCM (0.0625 M) were used.

^*g*^Alkene (5 equiv.), CrCl_2_ (10 mol%), **L5** (10 mol%), **11** (5 mol%), Mg(ClO_4_)_2_ (1 equiv.), and DCE (0.05 M) were used.

^*h*^Alkene (20 equiv.), CrCl_2_ (20 mol%), **L5** (20 mol%), **11** (5 mol%), MgPhPO_3_ (20 mol%), and DCE (0.1 M) were used. Reaction time was 48 h.

Linear alkenes were also competent substrates. Tetrasubstituted alkene **1d** reacted with various aldehydes, including *ortho*-, *meta*-, and *para*-substituted benzaldehydes, an electron-rich benzaldehyde, and a heteroaromatic aldehyde, affording the corresponding products **8l–8q** (containing an allylic quaternary carbon) with excellent enantioselectivity. The loading of alkene **1d** could be reduced to 2 equiv., likely due to the lower oxidation potential of **1d** relative to **1a–1c**. For less reactive aldehydes such as *o*-tolualdehyde and *p*-methoxy benzaldehyde, the chiral chromium alkoxide complex generated from CrCl_3_·3THF and NaO*t*-Bu^21^ exhibited higher catalytic activity than the CrCl_2_-derived species (**8m** and **8p**). We postulate that this is as a result of allylchromium species **6** bearing alkoxide ligands (X = OR) with higher nucleophilicity than those bearing electron-withdrawing chloride ligands (X = Cl).^22^,^23^ The reaction of aliphatic aldehydes also proceeded with high enantioselectivity (**8r–8u**) following minor modifications of the reaction conditions (1,2-dichloroethane [DCE] as the solvent, 20 mol% MgPhPO_3_ additive). In the case of asymmetric trisubstituted alkene **1e**, an inseparable mixture of **8v** and **8w** (itself a diastereomixture) was produced with moderate regioselectivity (regioisomeric ratio; rr = **8v**/**8w** = 1.9/1). Nevertheless, both the reactivity and enantioselectivity of **8v** were very high: use of 2.5 mol% and 0.5 mol% loadings of the chromium catalyst and photocatalyst **11**, respectively, led to the products in 97% combined yield, with **8v** in 96% ee. Major isomer **8v** presumably derives from prenylchromium species with the chromium atom at the terminal carbon, while minor isomer **8w** originates from 2-methyl but-2-enylchromium species with chromium at the terminal carbon. We anticipate that improving the regioselectivity so that the carbon-centered radical can be intercepted by the metal complex in the case of asymmetric alkenes will be a very important avenue for future research. In addition, linear terminal alkenes and disubstituted internal alkenes (*e.g.* 1-hexene and 2-butene) were unreactive under the current optimal conditions probably due to their high oxidation potentials.

The following experimental results provide key insights into the reaction mechanism (see ESI† for details). First, the addition of TEMPO (2,2,6,6-tetramethylpiperidinyloxyl) as a radical trapping agent to the reaction between **1a** and **2a** under otherwise optimized conditions completely inhibited the desired reaction. A TEMPO adduct of **1a** at the terminal carbon was detected by ^1^H NMR analysis of the crude mixture after the workup. This result supports our hypothesis that the reaction proceeds through carbon-centered radicals derived from alkene **1**. Second, we performed a radical clock experiment using 2-phenylcyclopropylcarbaldehyde and **1d**. The reaction proceeded with 77% yield without any cyclopropane ring-opening, indicating that ketyl radicals derived from aldehydes are not involved in the catalytic cycle. These findings, together with the observation that the presence of the chromium complex was essential for the reaction (Table 1, entry 1), are consistent with our working hypothesis for the reaction mechanism depicted in Fig. 2.

**Fig. 3:**
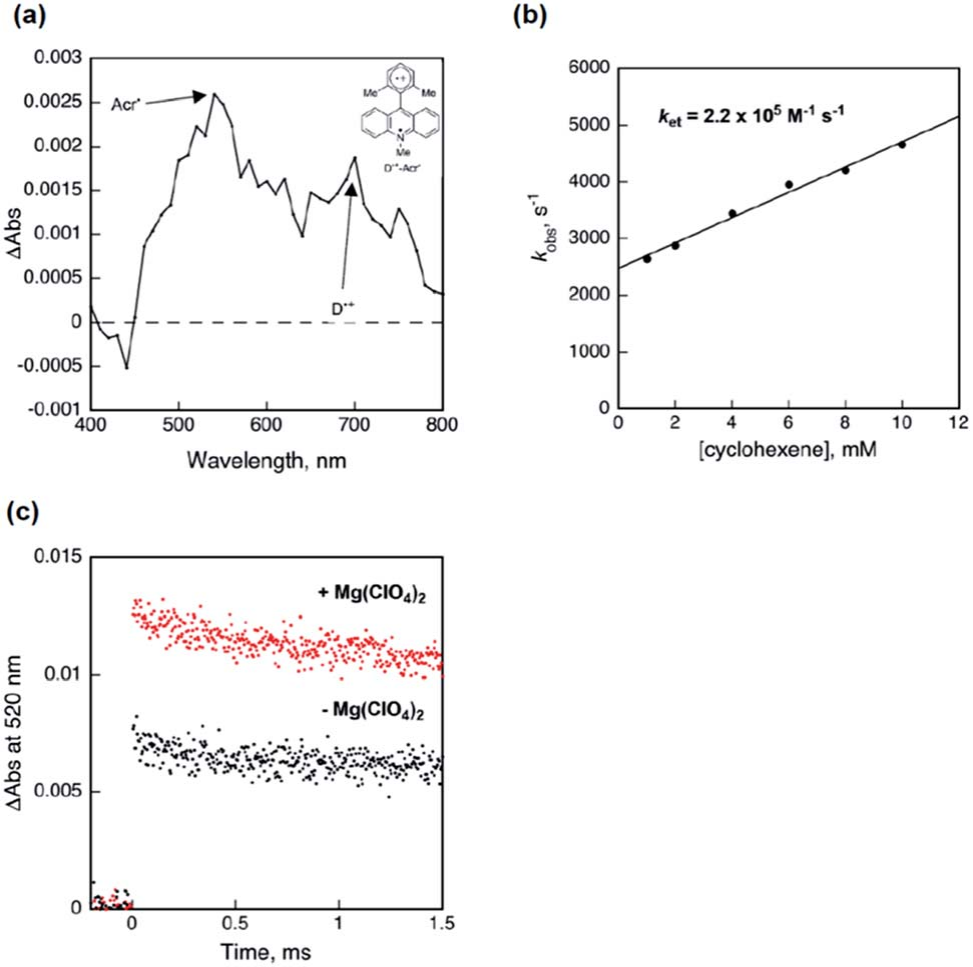
Detection of radical intermediates in the photocatalytic redox cycle. (a) Transient absorption spectrum of **10** (50 μM) measured in DCM at 200 μs after laser excitation at 355 nm. (b) Relationship between the decay rate constant (*k*_obs_) of the Xyl˙^+^ moiety observed at 700 nm and the concentration of **1a**. (c) Time profiles at 520 nm of Acr˙–Xyl generated by photoexcitation of Acr^+^–Xyl (50 μM) with cyclohexene (300 mM) in the absence (black) and presence (red) of Mg(ClO_4_)_2_.

To clarify the electron-transfer dynamics between **1a** and the photoexcited **10**, we examined the transient absorption measurements as shown in Fig. 3(a). Laser flash irradiation (*λ* = 355 nm) of **10** gave the electron-transfer state (Acr˙–Xyl˙^+^). The spectrum furnished absorption peaks at *λ*_max_ = 520 nm (ref. 24) and 700 nm,^25^ which attributed to the acridinyl radical moiety (Acr˙) and the xylyl radical cation moiety (Xyl˙^+^), respectively (Fig. 3(a)). The time profiles of the decay at 700 nm due to the Xyl˙^+^ moiety obeyed pseudo-first-order kinetics. The decay rate constant (*k*_obs_) linearly increased with the concentration of **1a** (Fig. 3(b)). The rate constant of electron transfer from **1a** to the acridinyl radical moiety of Acr˙–Xyl˙^+^ was determined from the linear plot to be 2.2 × 10^5^ M^–1^ s^–1^. Thus, electron transfer efficiently occurred to yield **3** and the reduced **10** (Acr˙–Xyl) as the initial step of the photocatalytic redox process.

Furthermore, to confirm that the dramatically increased reactivity in the presence of additive Mg(ClO_4_)_2_ was due to the increased concentration of the radical pair (**3** and Acr˙–Xyl) generated by electron transfer from **1a** to the electron-transfer state of **10** (Acr˙–Xyl˙^+^), we monitored the initial transient absorption intensities due to Acr˙–Xyl (*λ*_max_ = 520 nm) generated by laser irradiation of **10** with a large excess of **1a** (300 mM) in the absence and presence of Mg(ClO_4_)_2_ (Fig. 3(c)). The initial intensity in the presence of Mg(ClO_4_)_2_ was 1.9 times higher than that in the absence of Mg(ClO_4_)_2_ (Fig. 3(c)). The radical pair is efficiently stabilized in the presence of Mg(ClO_4_)_2_ salt by the electrostatic interaction of **3** with ClO_4_^–^.^20^ Therefore, the concentration of cation radical **3** is enhanced by the presence of Mg(ClO_4_)_2_, and this is likely the reason for the dramatically higher product yield in the presence of Mg(ClO_4_)_2_ (Table 1, entry 7 *vs.* 12).^26^

## Conclusions

In conclusion, we developed the first catalytic asymmetric allylation of aldehydes using unactivated hydrocarbon alkenes as pronucleophiles. The reaction enabled direct access to enantiomerically and diastereomerically-enriched homoallylic alcohols, starting from readily available and stable substrates. Critical to the success of the reaction was the development of an asymmetric hybrid catalyst system comprising an acridinium photoredox catalyst and a chiral chromium complex catalyst. The hybrid catalysis enabled a key radical–polar crossover process involving the catalytic generation of chiral and nucleophilic (*i.e.*, polar) organometallic species from simple alkenes *via* allylic C(sp^3^)–H activation. Further studies to improve the efficiency of the process, fully elucidate the reaction mechanism, and expand the substrate scope are ongoing.

## Conflicts of interest

There are no conflicts to declare.

## Supplementary Material

Supplementary information

Video abstract
