# Supplementary material for: Catalytic asymmetric allylation of aldehydes with alkenes through allylic C(sp3)–H functionalization mediated by organophotoredox and chiral chromium hybrid catalysis
Source: Chem Sci. 2019 Jan 17;10(12):3459–65. doi: 10.1039/c8sc05677c (PMC6430092; doi:10.1039/c8sc05677c)
Supplement: Supplementary file 1 [file SC-010-C8SC05677C-s001.pdf]

Electronic Supplementary Material (ESI) for Chemical Science.
This journal is © The Royal Society of Chemistry 2019

Supporting Information

Catalytic Asymmetric Allylation of Aldehydes with Alkenes through Allylic $C(sp^3)$–H Functionalization Mediated by Organophotoredox and Chiral Chromium Hybrid Catalysis

Harunobu Mitsunuma,*[,†] Shun Tanabe,[†] Hiromu Fuse,[†] Kei Ohkubo,[‡] and Motomu Kanai*[,†]

[†]Graduate School of Pharmaceutical Sciences, The University of Tokyo, Tokyo 113-0033, Japan

[‡]Institute for Advanced Co-Creation Studies and Open and Transdisciplinary Research Initiatives, Osaka University, Osaka 565-0871, Japan

*e-mail: h-mitsunuma@mol.f.u-tokyo.ac.jp; kanai@mol.f.u-tokyo.ac.jp

Contents

1. General Method
2. Preparation of Catalysts and Substrates
3. General Procedure for Catalytic Asymmetric Allylation of Aldehydes
4. Optimization Study for Aliphatic Aldehydes
5. Mechanistic Study
6. References
7. NMR Charts

1. General Method

^{1}H NMR spectra were recorded on JEOL ECX500 (500 MHz for ^{1}H NMR and 125.65 MHz for ^{13}C NMR), and JEOL ECS400 (400 MHz for ^{1}H NMR and 100 MHz for ^{13}C NMR, and 370 MHz for ^{19}F NMR) spectrometer. For ^{1}H NMR and ^{13}C NMR, chemical shifts were reported in the scale relative to $CDCl_3$ (δ = 7.26 for ^{1}H NMR and δ = 77.0 for ^{13}C NMR) used as an internal reference. Electrospray ionization (ESI)-mass spectra were measured on a JEOL JMS-T100LC AccuTOF spectrometer for HRMS. DART-mass spectra were measured on a JEOL JMS-T100LC AccuTOF spectrometer. Infrared (IR) spectra were recorded on a JASCO FT/IR 410 Fourier transform infrared spectrophotometer. The enantiomeric excess (ee) was determined by HPLC analysis (JASCO HPLC systems; pump: PU-986; detector: UV-2075, measured at 210 nm or 254 nm; column: CHIRALPAK IA3, IBN3, IC3, IF3). Optical rotations were measured on a JASCO P-1010 polarimeter. Column chromatographies were performed with silica gel Merck 60 (230-400 mesh ASTM), Biotage Isolera One and Biotage SNAP Ultra, or Yamazen Smart Flash and Universal Column Premium. All non-commercially available compounds were prepared and characterized as described in Section 5 of this SI. Other reagents were purchased from Aldrich, Tokyo Chemical Industry Co., Ltd. (TCI), Kanto Chemical Co., Inc., and Wako Pure Chemical Industries, Ltd., and were used as received. A Valore VBP-L24-C2 with 38W LED lamp (VBL-SE150-BBB (430)) was used as the 430 nm light source.

2. Preparation of Catalysts and Substrates

Alkenes (**1a**-**1e**), aldehydes (**2**), Cr catalysts ($CrCl_2$, $CrCl_3$•3THF), ligand **L2**, photocatalysts (**10**, **11**) and additives (LiCl, LiI, $LiBF_4$, $LiClO_4$, $NaClO_4$, $Ca(ClO_4)_2$•xH_2O, $Mg(ClO_4)_2$, NaO*t*Bu) were commercially available and used as purchased. Chiral ligands (**L1, L3**-**L5**) were prepared according to the reported method.[1-4] $MgPhPO_3$ was synthesized according to related procedure for the preparation of metal phosphates.[5]

Preparation of $MgPhPO_3$

To a solution of $Mg(OEt)_2$ (572 mg, 5 mmol) in EtOH (50 ml), phenylphosphonic acid (791 mg, 5 mmol) was added portion wise at 0 ºC. The mixture was stirred for 15 min and concentrated under vacuo. The white solid was used for next reaction without further purification.

3. General Procedure for Catalytic Asymmetric Allylation of Aldehydes

Procedure for preparation of 8a-8i, 8k

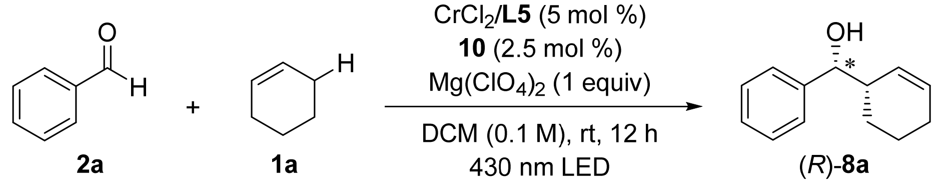

$CrCl_2$ (1.5 mg, 0.0125 mmol, 5 mol %) and **L5** (4.5 mg, 0.0125 mmol, 5 mol %) were dissolved in degassed

CH_2Cl_2 (2.5 mL) in a screw-capped vial under argon atmosphere and the mixture was stirred for 1 hour at room temperature. Cyclohexene **1a** (507 μL, 5.0 mmol, 20 eq), $Mg(ClO_4)_2$ (55.8 mg, 0.25 mmol, 100 mol %), benzaldehyde **2a** (25.5 μL, 0.25 mmol, 1 eq) and photocatalyst **10** (2.5 mg, 0.00625 mmol, 2.5 mol %) were added to the reaction mixture. The vial was subjected to 430 nm LED irradiation for 12 hours under temperature control (ca. 27–29 ºC). Then, the reaction mixture was passed through a pad of silica gel with CH_2Cl_2 elution. After evaporation, diastereomeric ratio was determined by NMR analysis (68%, >20/1). The residue was purified by silica gel flash column chromatography (CH_2Cl_2/hexane = 1/2 to 2/1, v/v) to afford (*R*)-**8a** as colorless oil (25.8 mg, 55%).

The amount of $CrCl_2$ and **L5** was increased to 10 mol% ($CrCl_2$: 3.0 mg, 0.025 mmol, L5: 9.0 mg, 0.025 mmol), (*R*)-**8a** was obtained in 65% (30.5 mg).

(*R*)-((*S*)-cyclohex-2-en-1-yl)(phenyl)methanol (8a).

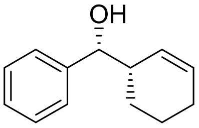

(*R*)-**8a**

All the spectroscopic data matches with the previously reported data.[6]

^{1}H NMR ($CDCl_3$, 500 MHz) δ 1.38-1.61 (m, 2H), 1.62-1.83 (m, 2H), 1.84-2.10 (m, 3H), 2.44-2.56 (m, 1H), 4.58 (d, J = 6.4 Hz, 1H), 5.38 (dd, J = 1.8, 10.1 Hz, 1H), 5.77-5.87 (m, 1H), 7.25-7.39 (m, 5H); ^{13}C NMR ($CDCl_3$, 125 MHz) δ 21.0, 23.8, 25.2, 43.0, 76.7, 126.5, 127.4, 128.0, 128.2, 130.4, 142.8; HPLC (chiral column: CHIRALPAK IA3; solvent: hexane/2-propanol = 200/1; flow rate: 1.0 mL/min; detection: at 210 nm): t_R = 26.7 min (minor) and 28.0 min (major); $[\alpha]_D^{20}$ = –4.8 (c = 0.76, $CHCl_3$, for 95% ee sample).

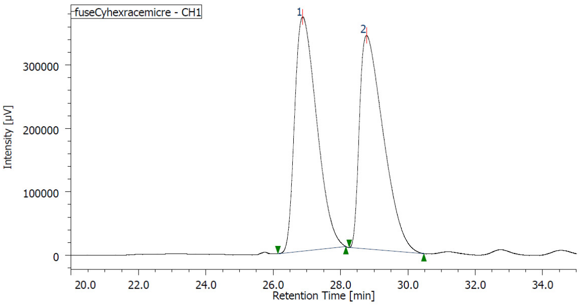

クロマトグラム情報	
ユーザー名	a
更新日時	2018/10/16 22:33:50
コメント	IA3 Hex IPA 200 1
HPLC システム名	HPLC-1
測定日	2018/10/16 17:43:26
注入量	10.00 [μL]
サンプル#	1
プロジェクト名	for you2
取込時間	90.0 [min]
測定シーケンス	fuseCyhexracemicre
コントロールメソッド	Morita_1.0ml_210nm
ピーク ID テーブル	
検量線テーブル	
追加情報	
チャンネル情報＋ピーク情報	
クロマトグラム名	fuseCyhexracemicre-CH1
サンプル名	
チャンネル名	CH1
データ取り込み間隔	500 [msec]
波形処理メソッド	（マニュアル）
数値計算式	
判定式	

#	ピーク名	CH	tR [min]	面積 [μV·sec]	高さ [μV]	面積%	高さ%	定量値	NTP	分離度	シンメトリー係数	警告
1	Unknown	1	26.875	16791441	368910	50.365	52.311	N/A	7741	1.502	1.560	
2	Unknown	1	28.775	16547901	336317	49.635	47.689	N/A	7663	N/A	2.008	

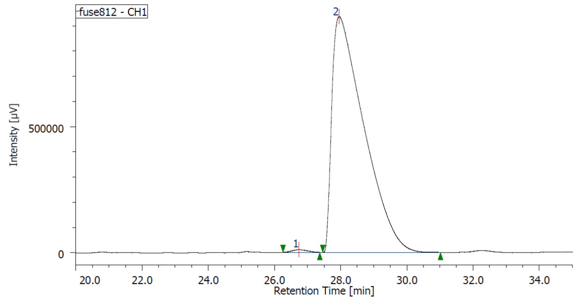

クロマトグラム情報

ユーザー名	a
更新日時	2018/10/17 19:02:17
コメント	IA3 Hex IPA 200 1
HPLC システム名	HPLC-1
測定日	2018/10/16 18:50:31
注入量	10.00 [µL]
サンプル#	1
プロジェクト名	for you2
取込時間	90.0 [min]
測定シーケンス	fuse812
コントロールメソッド	Morita_1.0ml_210nm
ピーク ID テーブル	
検量線テーブル	
追加情報	

チャンネル情報＋ピーク情報

クロマトグラム名	fuse812-CH1
サンプル名	
チャンネル名	CH1
データ取り込み間隔	500 [msec]
波形処理メソッド	（マニュアル）
数値計算式	
判定式	

#	ピーク名	CH	tR [min]	面積 [µV·sec]	高さ [µV]	面積%	高さ%	定量値	NTP	分離度	シンメトリー係数	警告
1	Unknown	1	26.742	354162	10343	0.560	1.093	N/A	12555	0.883	1.146	
2	Unknown	1	27.950	62936479	935934	99.440	98.907	N/A	3897	N/A	2.864	

(*R*)-(4-chlorophenyl)((*S*)-cyclohex-2-en-1-yl)methanol (8b).

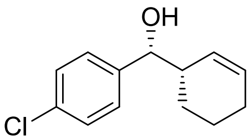

(*R*)-**8b**

Prepared according to the general procedure, then the crude material was purified by silica gel flash column chromatography (CH_2Cl_2/hexane = 1/10 to 1/0, v/v) to afford (*R*)-**8b** as colorless oil (45.8 mg, 82%).

IR (neat) ν 3396, 3022, 2927, 2861, 1698, 1651, 1597, 1541, 1492, 1410, 1090, 1013, 837, 725 cm^{-1}; ^1H NMR ($CDCl_3$, 500 MHz) δ 1.42-1.54 (m, 2 H), 1.58-1.68 (m, 1H), 1.69-1.78 (m, 1H), 1.86 (d, *J* = 2.3 Hz, 1H), 1.91-2.02 (m, 2H), 2.40-2.50 (m, 1H), 4.60 (dd, *J* = 2.3, 6.3 Hz, 1H), 5.39 (dd, *J* = 2.3, 10.3 Hz, 1H), 5.78-5.87 (m, 1H), 7.24-7.33 (m, 4H); ^{13}C NMR ($CDCl_3$, 125 MHz) δ 21.0, 23.6, 25.1, 43.0, 76.6, 127.5, 127.8, 128.3, 130.9, 132.9, 141.2; LRMS (ESI): *m*/*z* 245 [M+Na^+]; HRMS (ESI): *m*/*z* calculated for $C_{13}H_{15}ClNaO^+$ [M+Na^+]: 245.0704, found: 245.0701; HPLC (chiral column: CHIRALPAK IA3; solvent: hexane/2-propanol = 200/1; flow rate: 1.0 mL/min; detection: at 210 nm): t_R = 37.5 min (major) and 41.5 min (minor); $[\alpha]_D^{20}$ = –24.6 (*c* = 1.0, C_6H_6, for 99% ee sample).

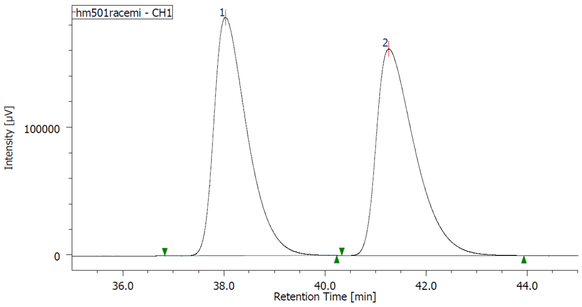

クロマトグラム情報

ユーザー名	a
更新日時	2018/09/17 22:15:48
コメント	IA3 Hex IPA 200 1
HPLC システム名	HPLC-2
測定日	2018/09/17 18:31:49
注入量	10.00 [µL]
サンプル#	1
プロジェクト名	for you2
取込時間	90.0 [min]
測定シーケンス	hm0495 496 501 racemi 200-1
コントロールメソッド	Morita_1.0ml_210nm
ピーク ID テーブル	
検量線テーブル	
追加情報	

チャンネル情報＋ピーク情報

クロマトグラム名	hm501racemi-CH1
サンプル名	
チャンネル名	CH1
データ取り込み間隔	500 [msec]
波形処理メソッド	（マニュアル）
数値計算式	
判定式	

#	ピーク名	CH	tR [min]	面積 [µV·sec]	高さ [µV]	面積%	高さ%	定量値	NTP	分離度	シンメトリー係数	警告
1	Unknown	1	38.033	8535464	186501	50.015	53.562	N/A	16680	2.546	1.686	
2	Unknown	1	41.258	8530335	161697	49.985	46.438	N/A	14687	N/A	1.836	

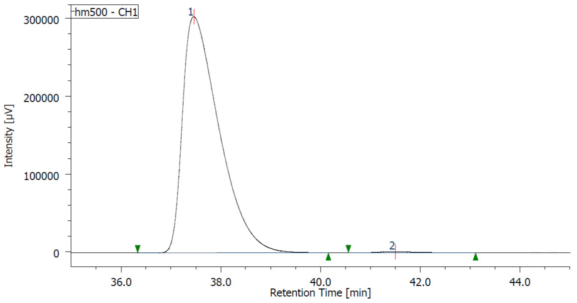

クロマトグラム情報	
ユーザー名	a
更新日時	2018/09/17 22:15:48
コメント	IA3 Hex IPA 200 1
HPLC システム名	HPLC-2
測定日	2018/09/17 21:24:23
注入量	10.00 [µL]
サンプル#	1
プロジェクト名	for you2
取込時間	90.0 [min]
測定シーケンス	hm0489 490 500 200-1
コントロールメソッド	Morita_1.0ml_210nm
ピーク ID テーブル	
検量線テーブル	
追加情報	

チャンネル情報＋ピーク情報	
クロマトグラム名	hm500-CH1
サンプル名	
チャンネル名	CH1
データ取り込み間隔	500 [msec]
波形処理メソッド	（マニュアル）
数値計算式	
判定式	

#	ピーク名	CH	tR [min]	面積 [µV·sec]	高さ [µV]	面積%	高さ%	定量値	NTP	分離度	シンメトリー係数	警告
1	Unknown	1	37.458	15486861	303150	99.535	99.511	N/A	12729	3.186	1.951	
2	Unknown	1	41.500	72335	1489	0.465	0.489	N/A	18639	N/A	1.336	

(*R*)-(4-bromophenyl)((*S*)-cyclohex-2-en-1-yl)methanol (8c).

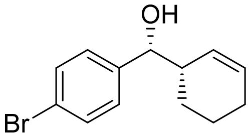

(*R*)-**8c**

Prepared according to the general procedure, then the crude material was purified by silica gel flash column chromatography (CH_2Cl_2/hexane = 1/10 to 1/0, v/v) to afford (*R*)-**8c** as colorless oil (54 mg, 81%).

IR (neat) ν 3396, 3022, 2927, 2860, 1651, 1592, 1488, 1404, 1072, 1010, 812, 725 cm^{-1}; ^{1}H NMR ($CDCl_3$, 500 MHz) δ 1.45-1.52 (m, 2 H), 1.57-1.68 (m, 1H), 1.70-1.78 (m, 1H), 1.85 (d, J = 2.9 Hz, 1H), 1.93-2.20 (m, 2H), 2.41-2.50 (m, 1H), 4.59 (dd, J = 2.3, 6.3 Hz, 1H), 5.39 (dd, J = 2.3, 10.3 Hz, 1H), 5.80-5.88 (m, 1H), 7.18-7.30 (m, 2H) , 7.43-7.49 (m, 2H); ^{13}C NMR ($CDCl_3$, 125 MHz) δ 21.2, 23.8, 25.4, 43.1, 77.5, 121.3, 127.8, 128.4, 131.1, 131.4, 142.0; LRMS (ESI): *m/z* 289 [M+Na$^+$]; HRMS (ESI): *m/z* calculated for $C_{13}H_{15}BrNaO^+$ [M+Na$^+$]: 289.0198, found: 289.0197; HPLC (chiral column: CHIRALPAK IA3; solvent: hexane/2-propanol = 200/1; flow rate: 1.0 mL/min; detection: at 210 nm): t_R = 42.0 min (major) and 46.6 min (minor); $[\alpha]_D^{20}$ = –5.8 (c = 0.66, $CHCl_3$, for 98% ee sample).

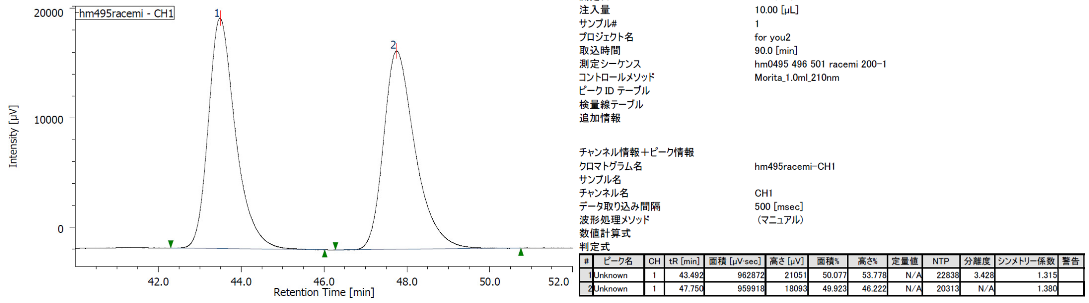

クロマトグラム情報	
ユーザー名	a
更新日時	2018/09/17 22:15:47
コメント	IA3 Hex IPA 200 1
HPLC システム名	HPLC-2
測定日	2018/09/17 16:30:36
注入量	10.00 [µL]
サンプル#	1
プロジェクト名	for you2
取込時間	90.0 [min]
測定シーケンス	hm0495 496 501 racemi 200-1
コントロールメソッド	Morita_1.0ml_210nm
ピーク ID テーブル	
検量線テーブル	
追加情報	

チャンネル情報＋ピーク情報	
クロマトグラム名	hm495racemi-CH1
サンプル名	
チャンネル名	CH1
データ取り込み間隔	500 [msec]
波形処理メソッド	（マニュアル）
数値計算式	
判定式	

#	ピーク名	CH	tR [min]	面積 [µV·sec]	高さ [µV]	面積%	高さ%	定量値	NTP	分離度	シンメトリー係数	警告
1	Unknown	1	43.492	962872	21051	50.077	53.778	N/A	22838	3.428	1.315	
2	Unknown	1	47.750	959918	18093	49.923	46.222	N/A	20313	N/A	1.380	

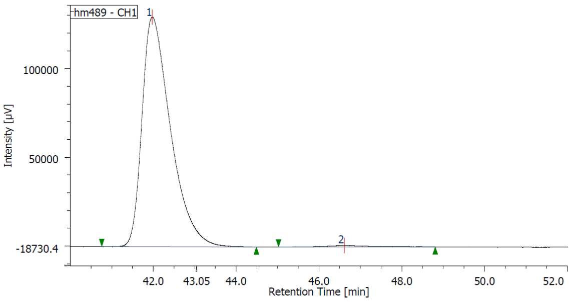

クロマトグラム情報	
ユーザー名	a
更新日時	2018/09/17 22:15:48
コメント	IA3 Hex IPA 200 1
HPLC システム名	HPLC-2
測定日	2018/09/17 19:34:38
注入量	10.00 [µL]
サンプル#	1
プロジェクト名	for you2
取込時間	90.0 [min]
測定シーケンス	hm0489 490 500 200-1
コントロールメソッド	Morita_1.0ml_210nm
ピーク ID テーブル	
検量線テーブル	
追加情報	
チャンネル情報＋ピーク情報	
クロマトグラム名	hm489-CH1
サンプル名	
チャンネル名	CH1
データ取り込み間隔	500 [msec]
波形処理メソッド	（マニュアル）
数値計算式	
判定式	

#	ピーク名	CH	tR [min]	面積 [µV·sec]	高さ [µV]	面積%	高さ%	定量値	NTP	分離度	シンメトリー係数	警告
1	Unknown	1	41.975	6196918	129351	99.165	99.431	N/A	18840	3.310	1.618	
2	Unknown	1	46.617	52173	740	0.835	0.569	N/A	13766	N/A	1.279	

(*R*)-((*S*)-cyclohex-2-en-1-yl)(4-iodophenyl)methanol (8d).

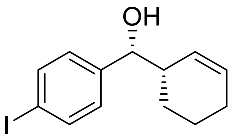

(*R*)-**8d**

Prepared according to the general procedure, then the crude material was purified by silica gel flash column chromatography (CH_2Cl_2/hexane = 1/10 to 1/0, v/v) to afford (*R*)-**8d** as colorless oil (36.3 mg, 46%).

IR (neat) ν 3392, 3021, 2926, 2859, 1698, 1651, 1588, 1481, 1399, 1059, 1004, 811, 725 cm^{-1}; ^1H NMR ($CDCl_3$, 500 MHz) δ 1.42-1.54 (m, 2 H), 1.59-1.67 (m, 1H), 1.68-1.79 (m, 1H), 1.86 (d, J = 1.7 Hz, 1H), 1.93-2.20 (m, 2H), 2.39-2.50 (m, 1H), 4.57 (dd, J = 1.7, 6.3 Hz, 1H), 5.39 (dd, J = 2.3, 10.3 Hz, 1H), 5.78-5.89 (m, 1H), 7.10 (d, J = 8.3 Hz, 2H), 7.67 (d, J = 8.3 Hz, 2H); ^{13}C NMR ($CDCl_3$, 125 MHz) δ 21.0, 23.5, 25.1, 42.9, 76.6, 92.7, 127.5, 128.5, 130.9, 137.2, 142.4; LRMS (ESI): *m*/*z* 337 [M+Na$^+$]; HRMS (ESI): *m*/*z* calculated for $C_{13}H_{15}INaO^+$ [M+Na$^+$]: 337.0060, found: 337.0073; HPLC (chiral column: CHIRALPAK IA3; solvent: hexane/2-propanol = 200/1; flow rate: 1.0 mL/min; detection: at 210 nm): t_R = 47.1 min (major) and 51.3 min (minor); $[\alpha]_D^{20}$ = –6.3 (c = 2.7, $CHCl_3$, for 99% ee sample).

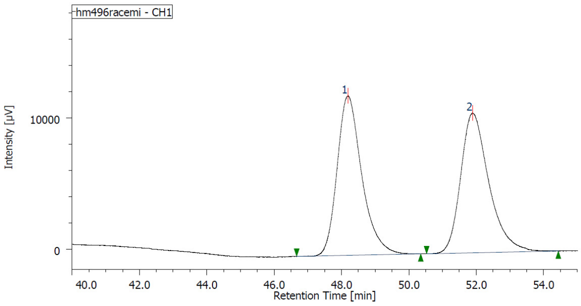

クロマトグラム情報	
ユーザー名	a
更新日時	2018/09/17 22:15:48
コメント	IA3 Hex IPA 200 1
HPLC システム名	HPLC-2
測定日	2018/09/17 17:35:01
注入量	10.00 [µL]
サンプル#	1
プロジェクト名	for you2
取込時間	90.0 [min]
測定シーケンス	hm0495 496 501 racemi 200-1
コントロールメソッド	Morita_1.0ml_210nm
ピーク ID テーブル	
検量線テーブル	
追加情報	
チャンネル情報＋ピーク情報	
クロマトグラム名	hm496racemi-CH1
サンプル名	
チャンネル名	CH1
データ取り込み間隔	500 [msec]
波形処理メソッド	（マニュアル）
数値計算式	
判定式	

#	ピーク名	CH	tR [min]	面積 [µV·sec]	高さ [µV]	面積%	高さ%	定量値	NTP	分離度	シンメトリー係数	警告
1	Unknown	1	48.192	617959	12130	49.923	53.293	N/A	22401	2.693	1.292	
2	Unknown	1	51.892	619873	10632	50.077	46.707	N/A	20000	N/A	1.341	

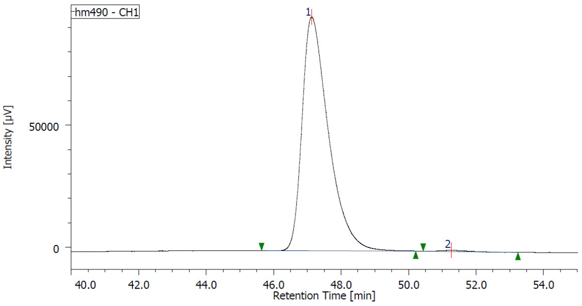

クロマトグラム情報
ユーザー名 a
更新日時 2018/09/17 22:15:48
コメント IA3 Hex IPA 200 1
HPLC システム名 HPLC-2
測定日 2018/09/17 20:27:13
注入量 10.00 [μL]
サンプル# 1
プロジェクト名 for you2
取込時間 90.0 [min]
測定シーケンス hm0489 490 500 200-1
コントロールメソッド Morita_1.0ml_210nm
ピーク ID テーブル
検量線テーブル
追加情報

チャンネル情報＋ピーク情報
クロマトグラム名 hm490-CH1
サンプル名
チャンネル名 CH1
データ取り込み間隔 500 [msec]
波形処理メソッド （マニュアル）
数値計算式
判定式

#	ピーク名	CH	tR [min]	面積 [μV·sec]	高さ [μV]	面積%	高さ%	定量値	NTP	分離度	シンメトリー係数	警告
1	Unknown	1	47.125	5199895	95799	99.450	99.430	N/A	18759	2.993	1.599	
2	Unknown	1	51.258	28751	549	0.550	0.570	N/A	21657	N/A	1.314	

1-(3-((*R*)-((*S*)-cyclohex-2-en-1-yl)(hydroxy)methyl)phenyl)ethan-1-one (8e).

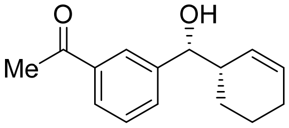

(*R*)-**8e**

Prepared according to the general procedure, then the crude material was purified by silica gel flash column chromatography (AcOEt/hexane = 1/10 to 1/1, v/v) to afford (*R*)-**8e** as colorless oil (26.7 mg, 46%).

IR (neat) ν 3444, 3021, 2927, 2860, 1683, 1601, 1584, 1433, 1360, 1276, 1184, 1025, 699 cm^{-1}; ^{1}H NMR ($CDCl_3$, 500 MHz) δ 1.45-1.54 (m, 2 H), 1.58-1.67 (m, 1H), 1.71-1.81 (m, 1H), 1.92 (d, *J* = 2.3 Hz, 1H), 1.96-2.03 (m, 2H), 2.48-2.57 (m, 1H), 2.62 (s, 3H), 4.71 (dd, *J* = 1.7, 5.7 Hz, 1H), 5.41 (dd, *J* = 1.7, 9.7 Hz, 1H), 5.83-5.90 (m, 1H), 7.45 (dd, *J* = 7.7, 7.7 Hz, 1H), 7.57 (d, *J* = 7.7 Hz, 1H), 7.87 (d, *J* = 7.7 Hz, 1H), 7.93 (s, 1H); ^{13}C NMR ($CDCl_3$, 125 MHz) δ 21.0, 23.4, 25.1, 26.7, 43.0, 76.8, 126.3, 127.4, 127.5, 128.4, 131.0, 131.2, 137.0, 143.4, 198.3; LRMS (DART): *m*/*z* 231 [$M+H^+$]; HRMS (DART): *m*/*z* calculated for $C_{15}H_{19}O_2^+$ [$M+H^+$]: 231.1380, found: 231.1371; HPLC (chiral column: CHIRALPAK IBN3; solvent: hexane/2-propanol = 50/1; flow rate: 1.0 mL/min; detection: at 210 nm): t_R = 42.4 min (minor) and 54.4 min (major); $[\alpha]_D^{20}$ = +6.9 (*c* = 0.54, $CHCl_3$, for 99% ee sample).

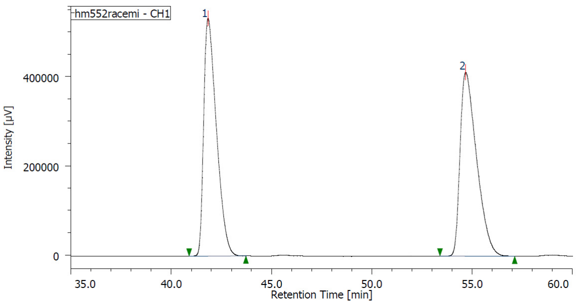

クロマトグラム情報
ユーザー名 a
更新日時 2018/12/01 17:14:31
コメント IBN3 Hex IPA 50 1
HPLC システム名 HPLC-2
測定日 2018/12/01 16:12:49
注入量 10.00 [μL]
サンプル# 1
プロジェクト名 for you2
取込時間 90.0 [min]
測定シーケンス hm0552 525
コントロールメソッド Morita_1.0ml_210nm
ピーク ID テーブル
検量線テーブル
追加情報

チャンネル情報＋ピーク情報
クロマトグラム名 hm552racemi-CH1
サンプル名
チャンネル名 CH1
データ取り込み間隔 500 [msec]
波形処理メソッド （マニュアル）
数値計算式
判定式

#	ピーク名	CH	tR [min]	面積 [μV·sec]	高さ [μV]	面積%	高さ%	定量値	NTP	分離度	シンメトリー係数	警告
1	Unknown	1	41.833	23520150	532949	49.933	56.400	N/A	20575	9.626	1.677	
2	Unknown	1	54.675	23583086	411993	50.067	43.600	N/A	21015	N/A	1.708	

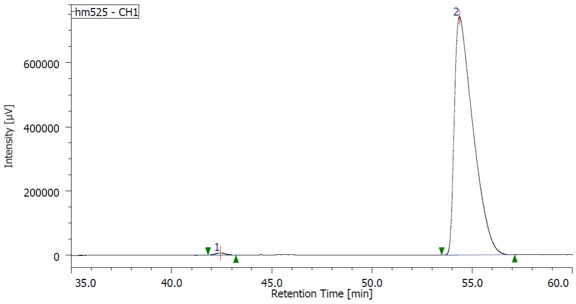

クロマトグラム情報
ユーザー名 a
更新日時 2018/12/01 18:18:33
コメント IBN3 Hex IPA 50 1
HPLC システム名 HPLC-2
測定日 2018/12/01 17:14:37
注入量 10.00 [μL]
サンプル# 1
プロジェクト名 for you2
取込時間 90.0 [min]
測定シーケンス hm0552 525
コントロールメソッド Morita_1.0ml_210nm
ピーク ID テーブル
検量線テーブル
追加情報

チャンネル情報＋ピーク情報
クロマトグラム名 hm525-CH1
サンプル名
チャンネル名 CH1
データ取り込み間隔 500 [msec]
波形処理メソッド （マニュアル）
数値計算式
判定式

#	ピーク名	CH	tR [min]	面積 [μV·sec]	高さ [μV]	面積%	高さ%	定量値	NTP	分離度	シンメトリー係数	警告
1	Unknown	1	42.425	250217	6803	0.518	0.908	N/A	29607	8.882	1.094	
2	Unknown	1	54.358	48030498	742411	99.482	99.092	N/A	16209	N/A	2.235	

Methyl 4-((*R*)-((*S*)-cyclohex-2-en-1-yl)(hydroxy)methyl)benzoate (8f).

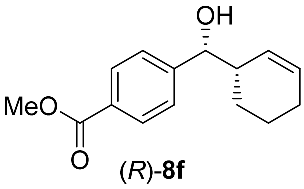

(*R*)-**8f**

Prepared according to the general procedure, then the crude material was purified by silica gel flash column chromatography (AcOEt/hexane = 1/10 to 1/1, v/v) to afford (*R*)-**8f** as colorless oil (49.4 mg, 80%). All the spectroscopic data matches with the previously reported data.[7]

^{1}H NMR ($CDCl_3$, 500 MHz) δ 1.44-1.62 (m, 1H), 1.66-1.80 (m, 1H), 1.90-2.02 (m, 3H), 2.41-2.46 (m, 1H), 3.91 (s, 3H), 4.70 (dd, J = 2.3, 6.3 Hz, 1H), 5.42 (dd, J = 1.7, 10.3 Hz, 1H), 5.84-5.90 (m, 1H), 7.42 (d, J = 8.3 Hz, 2H), 8.02 (d, J = 8.3 Hz, 2H); ^{13}C NMR ($CDCl_3$, 125 MHz) δ 21.0, 23.3, 25.1, 42.9, 52.0, 76.6, 126.4, 127.5, 129.0, 129.4, 131.0, 148.1, 167.0; HPLC (chiral column: CHIRALPAK IA3; solvent: hexane/2-propanol = 50/1; flow rate: 1.0 mL/min; detection: at 210 nm): t_R = 38.8 min (major) and 55.2 min (minor); $[\alpha]_D^{20.9}$ = +5.1 (c = 0.97, $CHCl_3$, for 99% ee sample).

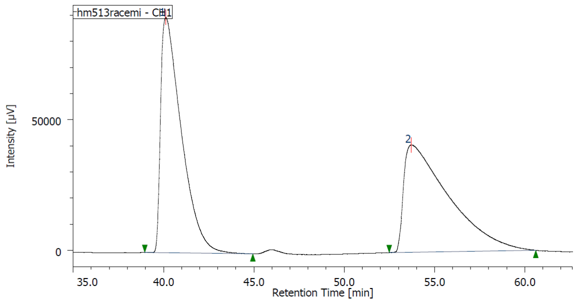

クロマトグラム情報
ユーザー名 a
更新日時 2018/12/01 17:15:54
コメント IA3 Hex IPA 50 1
HPLC システム名 HPLC-1
測定日 2018/12/01 16:10:16
注入量 10.00 [μL]
サンプル# 1
プロジェクト名 for you2
取込時間 90.0 [min]
測定シーケンス hm0510 513
コントロールメソッド Morita_1.0ml_210nm
ピーク ID テーブル
検量線テーブル
追加情報

チャンネル情報＋ピーク情報
クロマトグラム名 hm513racemi-CH1
サンプル名
チャンネル名 CH1
データ取り込み間隔 500 [msec]
波形処理メソッド （マニュアル）
数値計算式
判定式

#	ピーク名	CH	tR [min]	面積 [μV·sec]	高さ [μV]	面積%	高さ%	定量値	NTP	分離度	シンメトリー係数	警告
1	Unknown	1	40.117	7185891	90204	50.144	68.714	N/A	6147	4.200	2.689	
2	Unknown	1	53.708	7144503	41071	49.856	31.286	N/A	2339	N/A	4.133	

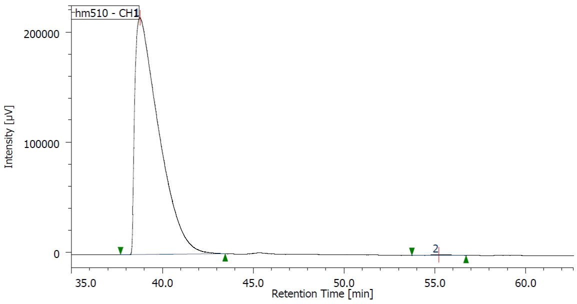

クロマトグラム情報
ユーザー名 a
更新日時 2018/12/01 18:18:41
コメント IA3 Hex IPA 50 1
HPLC システム名 HPLC-1
測定日 2018/12/01 17:16:02
注入量 10.00 [µL]
サンプル# 1
プロジェクト名 for you2
取込時間 90.0 [min]
測定シーケンス hm0510 513
コントロールメソッド Morita_1.0ml_210nm
ピーク ID テーブル
検量線テーブル
追加情報

チャンネル情報＋ピーク情報
クロマトグラム名 hm510-CH1
サンプル名
チャンネル名 CH1
データ取り込み間隔 500 [msec]
波形処理メソッド （マニュアル）
数値計算式
判定式

#	ピーク名	CH	tR [min]	面積 [µV·sec]	高さ [µV]	面積%	高さ%	定量値	NTP	分離度	シンメトリー係数	警告
1	Unknown	1	38.758	19708225	215085	99.778	99.736	N/A	4178	7.160	3.592	
2	Unknown	1	55.217	43919	570	0.222	0.264	N/A	9978	N/A	1.300	

(*R*)-((*S*)-cyclohex-2-en-1-yl)(4-(trifluoromethyl)phenyl)methanol (8g).

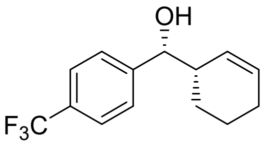

(*R*)-**8g**

Prepared according to the general procedure, then the crude material was purified by silica gel flash column chromatography (CH_2Cl_2/hexane = 1/10 to 1/0, v/v) to afford (*R*)-**8g** as colorless oil (40.2 mg, 63%). All the spectroscopic data matches with the previously reported data.[8]

^{1}H NMR ($CDCl_3$, 500 MHz) δ 1.45-1.62 (m, 2H), 1.70-1.81 (m, 1H), 1.91 (d, J = 2.3 Hz, 1H), 1.97-2.03 (m, 2H), 2.47-2.56 (m, 1H), 4.71 (d, J = 8.0 Hz, 2H), 5.42 (dd, J = 2.3, 10.3 Hz, 1H), 5.85-5.92 (m, 1H), 7.47 (d, J = 8.0 Hz, 2H), 7.60 (d, J = 8.3 Hz, 2H); ^{13}C NMR ($CDCl_3$, 125 MHz) δ 21.0, 23.2, 25.1, 43.0, 76.5, 124.2 (q, J = 270.7 Hz), 125.1 (q, J = 4.8 Hz), 126.7, 127.3, 129.5 (q, J = 32.2 Hz), 131.3, 146.7; HPLC (chiral column: CHIRALPAK IBN3; solvent: hexane/2-propanol = 200/1; flow rate: 1.0 mL/min; detection: at 210 nm): t_R = 23.4 min (major) and 24.6 min (minor); $[\alpha]_D^{20}$ = +8.6 (c = 1.1, $CHCl_3$, for 99% ee sample).

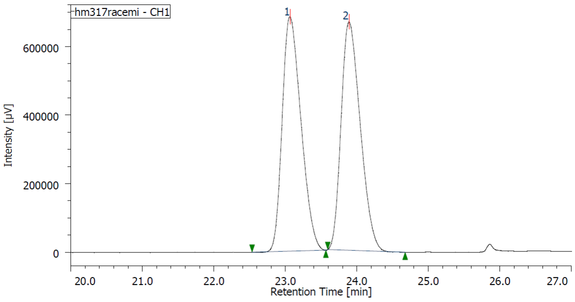

クロマトグラム情報
ユーザー名 a
更新日時 2018/10/17 21:38:04
コメント IBN3 Hex IPA 200 1
HPLC システム名 HPLC-2
測定日 2018/10/17 20:00:15
注入量 10.00 [µL]
サンプル# 1
プロジェクト名 for you2
取込時間 90.0 [min]
測定シーケンス hm0317racemi2
コントロールメソッド Morita_1.0ml_210nm
ピーク ID テーブル
検量線テーブル
追加情報

チャンネル情報＋ピーク情報
クロマトグラム名 hm317racemi-CH1
サンプル名
チャンネル名 CH1
データ取り込み間隔 500 [msec]
波形処理メソッド （マニュアル）
数値計算式
判定式

#	ピーク名	CH	tR [min]	面積 [µV·sec]	高さ [µV]	面積%	高さ%	定量値	NTP	分離度	シンメトリー係数	警告
1	Unknown	1	23.067	12490038	683977	50.190	50.659	N/A	35614	1.677	1.284	
2	Unknown	1	23.892	12395656	666173	49.810	49.341	N/A	36857	N/A	1.297	

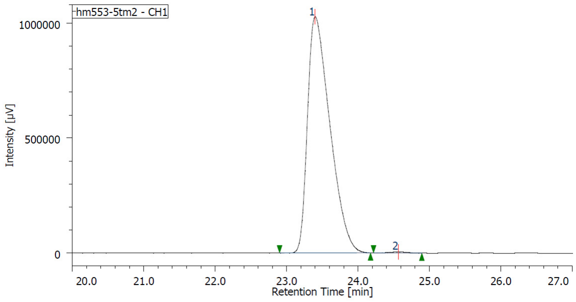

クロマトグラム情報	
ユーザー名	a
更新日時	2018/10/17 21:38:05
コメント	IBN3 Hex IPA 200 1
HPLC システム名	HPLC-2
測定日	2018/10/17 21:06:17
注入量	10.00 [µL]
サンプル#	1
プロジェクト名	for you2
取込時間	90.0 [min]
測定シーケンス	hm0553-5tm2
コントロールメソッド	Morita_1.0ml_210nm
ピーク ID テーブル	
検量線テーブル	
追加情報	
チャンネル情報＋ピーク情報	
クロマトグラム名	hm553-5tm2-CH1
サンプル名	
チャンネル名	CH1
データ取り込み間隔	500 [msec]
波形処理メソッド	（マニュアル）
数値計算式	
判定式	

#	ピーク名	CH	tR [min]	面積 [µV·sec]	高さ [µV]	面積%	高さ%	定量値	NTP	分離度	シンメトリー係数	警告
1	Unknown	1	23.400	22414982	1027720	99.597	99.538	N/A	26123	2.114	1.638	
2	Unknown	1	24.567	90689	4774	0.403	0.462	N/A	34718	N/A	1.001	

(*R*)-((*S*)-cyclopent-2-en-1-yl)(phenyl)methanol (8h).

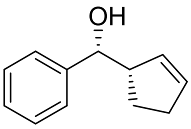

(*R*)-**8h**

Prepared according to the general procedure, then the crude material was purified by silica gel flash column chromatography (CH_2Cl_2/hexane = 1/2 to 2/1, v/v) to afford (*R*)-**8h** as colorless oil (25.5 mg, 59%). All the spectroscopic data matches with the previously reported data.[6]

^{1}H NMR ($CDCl_3$, 500 MHz) δ 1.78-2.00 (m, 3H), 2.24-2.45 (m, 2H), 3.05-3.17 (m, 1H), 4.58 (d, J = 6.4 Hz, 1H), 5.38-5.44 (m, 1H), 5.83-5.92 (m, 1H), 7.22-7.42 (m, 5H); ^{13}C NMR ($CDCl_3$, 125 MHz) δ 25.0, 32.2, 53.9, 76.7, 126.2, 127.3, 128.2, 131.2, 133.7, 143.4; HPLC (chiral column: CHIRALPAK IA3; solvent: hexane/2-propanol = 200/1; flow rate: 1.0 mL/min; detection: at 210 nm): t_R = 25.2 min (minor) and 26.1 min (major); $[\alpha]_D^{20}$ = –67 (c = 1.56, $CHCl_3$, for 99% ee sample).

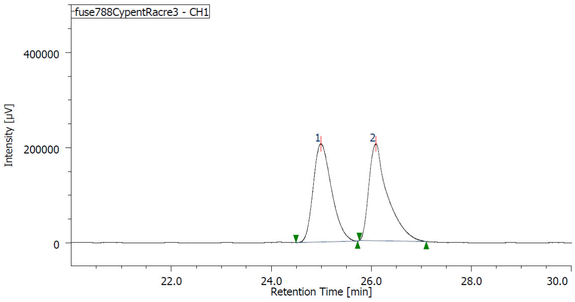

クロマトグラム情報	
ユーザー名	a
更新日時	2018/09/22 13:56:13
コメント	IA3 Hex IPA 200 1
HPLC システム名	HPLC-2
測定日	2018/09/22 11:43:27
注入量	10.00 [µL]
サンプル#	1
プロジェクト名	for you2
取込時間	90.0 [min]
測定シーケンス	fuse788Cypentracre3
コントロールメソッド	Morita_1.0ml_210nm
ピーク ID テーブル	
検量線テーブル	
追加情報	
チャンネル情報＋ピーク情報	
クロマトグラム名	fuse788CypentRacre3-CH1
サンプル名	
チャンネル名	CH1
データ取り込み間隔	500 [msec]
波形処理メソッド	（マニュアル）
数値計算式	
判定式	

#	ピーク名	CH	tR [min]	面積 [µV·sec]	高さ [µV]	面積%	高さ%	定量値	NTP	分離度	シンメトリー係数	警告
1	Unknown	1	24.992	5247381	206143	49.799	50.320	N/A	22315	1.705	1.316	
2	Unknown	1	26.092	5289771	203524	50.201	49.680	N/A	27919	N/A	1.797	

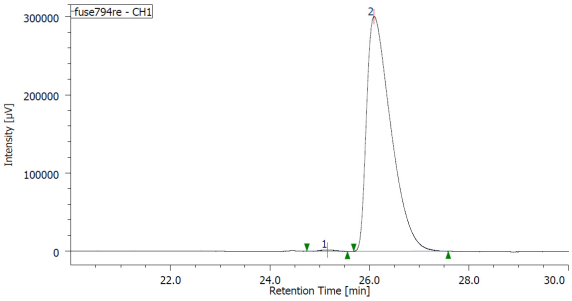

クロマトグラム情報
ユーザー名 a
更新日時 2018/09/22 21:32:14
コメント IA3 Hex IPA 200 1
HPLC システム名 HPLC-2
測定日 2018/09/22 14:50:48
注入量 10.00 [μL]
サンプル# 1
プロジェクト名 for you2
取込時間 90.0 [min]
測定シーケンス fuse794re
コントロールメソッド Morita_1.0ml_210nm
ピーク ID テーブル
検量線テーブル
追加情報

チャンネル情報＋ピーク情報
クロマトグラム名 fuse794re-CH1
サンプル名
チャンネル名 CH1
データ取り込み間隔 500 [msec]
波形処理メソッド （マニュアル）
数値計算式
判定式

#	ピーク名	CH	tR [min]	面積 [μV·sec]	高さ [μV]	面積%	高さ%	定量値	NTP	分離度	シンメトリー係数	警告
1	Unknown	1	25.158	40605	1835	0.398	0.607	N/A	26670	1.242	1.079	
2	Unknown	1	26.092	10165615	300336	99.602	99.393	N/A	13736	N/A	1.982	

(*R*)-(4-chlorophenyl)((*S*)-cyclopent-2-en-1-yl)methanol (8i).

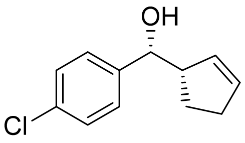

(*R*)-**8i**

Prepared according to the general procedure, then the crude material was purified by silica gel flash column chromatography (CH_2Cl_2/hexane = 1/2 to 2/1, v/v) to afford (*R*)-**8i** as colorless solid (28.5 mg, 55%).

IR (neat) ν 3390, 3053, 2929, 2850, 1902, 1716, 1597, 1489, 1410, 1293, 1089, 1013, 827, 726 cm^{-1}; ^{1}H NMR ($CDCl_3$, 500 MHz) δ 1.72-1.97 (m, 3H), 2.23-2.43 (m, 2H), 3.01-3.12 (m, 1H), 4.58 (d, *J* = 6.4 Hz, 1H), 5.37-5.45 (m, 1H), 5.84-5.93 (m, 1H), 7.24-7.34 (m, 4H); ^{13}C NMR ($CDCl_3$, 125 MHz) δ 24.6, 32.2, 53.9, 76.0, 127.6, 128.3, 130.8, 132.9, 134.2, 141.8; LRMS (ESI): *m*/*z* 231 [$M+Na^+$]; HRMS (ESI): *m*/*z* calculated for $C_{12}H_{13}ClNaO^+$ [$M+Na^+$]: 231.0547, found: 231.0550; HPLC (chiral column: CHIRALPAK IC3; solvent: hexane/2-propanol = 200/1; flow rate: 1.0 mL/min; detection: at 210 nm): t_R = 16.4 min (major) and 20.8 min (minor); $[\alpha]_D^{20}$ = –60 (*c* = 0.98, $CHCl_3$, for 99% ee sample).

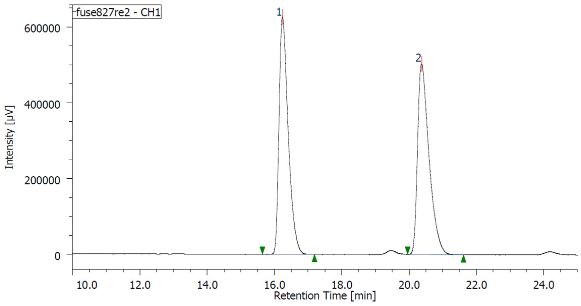

クロマトグラム情報
ユーザー名 a
更新日時 2018/10/17 16:11:46
コメント IC3 Hex IPA 200 1
HPLC システム名 HPLC-2
測定日 2018/10/17 15:24:12
注入量 10.00 [μL]
サンプル# 1
プロジェクト名 for you2
取込時間 90.0 [min]
測定シーケンス fuse827re2
コントロールメソッド Morita_1.0ml_210nm
ピーク ID テーブル
検量線テーブル
追加情報

チャンネル情報＋ピーク情報
クロマトグラム名 fuse827re2-CH1
サンプル名
チャンネル名 CH1
データ取り込み間隔 500 [msec]
波形処理メソッド （マニュアル）
数値計算式
判定式

#	ピーク名	CH	tR [min]	面積 [μV·sec]	高さ [μV]	面積%	高さ%	定量値	NTP	分離度	シンメトリー係数	警告
1	Unknown	1	16.233	11877023	626219	49.969	55.473	N/A	17705	7.582	1.593	
2	Unknown	1	20.375	11891615	502642	50.031	44.527	N/A	18000	N/A	1.666	

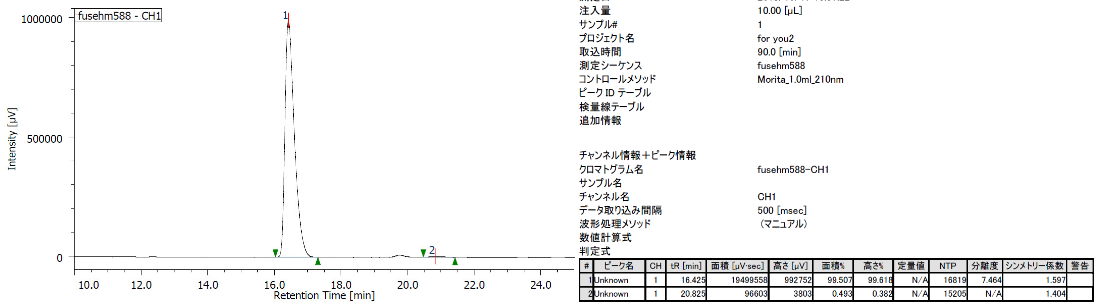

クロマトグラム情報
ユーザー名 a
更新日時 2018/10/17 17:11:23
コメント IC3 Hex IPA 200 1
HPLC システム名 HPLC-2
測定日 2018/10/17 16:37:22
注入量 10.00 [µL]
サンプル# 1
プロジェクト名 for you2
取込時間 90.0 [min]
測定シーケンス fusehm588
コントロールメソッド Morita_1.0ml_210nm
ピーク ID テーブル
検量線テーブル
追加情報

チャンネル情報＋ピーク情報
クロマトグラム名 fusehm588-CH1
サンプル名
チャンネル名 CH1
データ取り込み間隔 500 [msec]
波形処理メソッド （マニュアル）
数値計算式
判定式

#	ピーク名	CH	tR [min]	面積 [µV·sec]	高さ [µV]	面積%	高さ%	定量値	NTP	分離度	シンメトリー係数	警告
1	Unknown	1	16.425	19499558	992752	99.507	99.618	N/A	16819	7.464	1.597	
2	Unknown	1	20.825	96603	3803	0.493	0.382	N/A	15205	N/A	1.404	

(*R*)-(4-chlorophenyl)((*S*)-cyclohept-2-en-1-yl)methanol (8k).

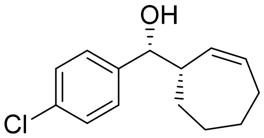

(*R*)-**8k**

Prepared according to the general procedure, then the crude material was purified by silica gel flash column chromatography (CH_2Cl_2/hexane = 1/2 to 2/1, v/v) to afford (*R*)-**8k** as colorless solid (28.1 mg, 47%).

IR (neat) ν 3389, 3020, 2923, 2852, 1901, 1721, 1650, 1597, 1490, 1444, 1090, 1013, 832, 687 cm^{-1}; ^{1}H NMR ($CDCl_3$, 500 MHz) δ 1.23-1.41 (m, 2H), 1.47-1.72 (m, 2H), 1.77-2.21 (m, 5H), 2.56-2.65 (m, 1H), 4.65 (d, J = 6.3 Hz, 1H), 5.46-5.54 (m, 1H), 5.75-5.84 (m, 1H), 7.21-7.37 (m, 4H); ^{13}C NMR ($CDCl_3$, 125 MHz) δ 26.7, 27.9, 28.5, 29.8, 47.0, 76.7, 128.0, 128.4, 132.8, 133.1, 133.1, 141.6; LRMS (ESI): m/z 259[$M+Na^+$]; HRMS (ESI): m/z calculated for $C_{14}H_{17}ClNaO^+$ [$M+Na^+$]: 259.0860, found: 259.0854; HPLC (chiral column: CHIRALPAK IA3; solvent: hexane/2-propanol = 200/1; flow rate: 1.0 mL/min; detection: at 210 nm): t_R = 43.8 min (major) and 50.6 min (minor); $[\alpha]_D^{20.9}$ = +63 (c = 0.92, $CHCl_3$, for 99% ee sample).

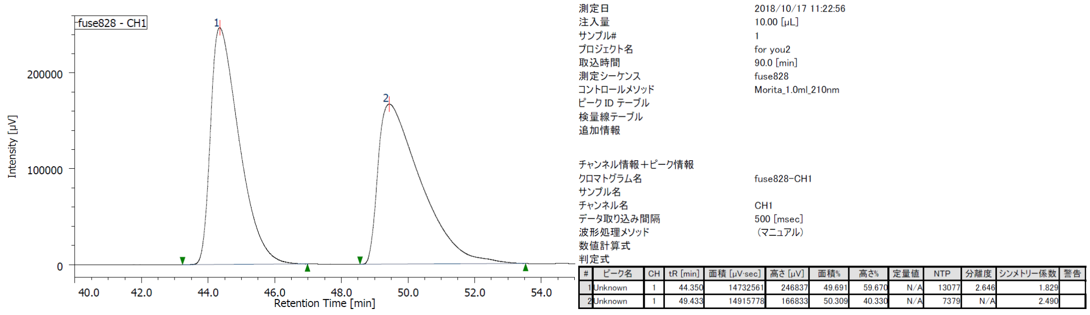

クロマトグラム情報
ユーザー名 a
更新日時 2018/10/17 13:18:22
コメント IA3 Hex IPA 200 1
HPLC システム名 HPLC-1
測定日 2018/10/17 11:22:56
注入量 10.00 [µL]
サンプル# 1
プロジェクト名 for you2
取込時間 90.0 [min]
測定シーケンス fuse828
コントロールメソッド Morita_1.0ml_210nm
ピーク ID テーブル
検量線テーブル
追加情報

チャンネル情報＋ピーク情報
クロマトグラム名 fuse828-CH1
サンプル名
チャンネル名 CH1
データ取り込み間隔 500 [msec]
波形処理メソッド （マニュアル）
数値計算式
判定式

#	ピーク名	CH	tR [min]	面積 [µV·sec]	高さ [µV]	面積%	高さ%	定量値	NTP	分離度	シンメトリー係数	警告
1	Unknown	1	44.350	14732561	246837	49.691	59.670	N/A	13077	2.646	1.829	
2	Unknown	1	49.433	14915778	166833	50.309	40.330	N/A	7379	N/A	2.490	

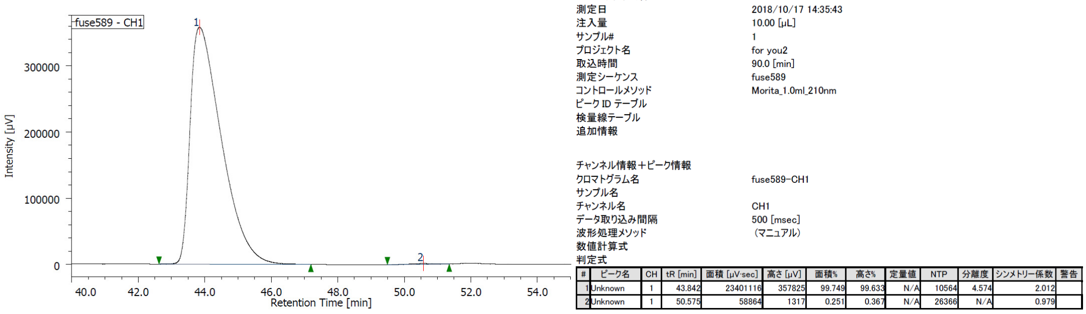

クロマトグラム情報
ユーザー名 a
更新日時 2018/10/17 15:46:17
コメント IA3 Hex IPA 200 1
HPLC システム名 HPLC-1
測定日 2018/10/17 14:35:43
注入量 10.00 [µL]
サンプル# 1
プロジェクト名 for you2
取込時間 90.0 [min]
測定シーケンス fuse589
コントロールメソッド Morita_1.0ml_210nm
ピーク ID テーブル
検量線テーブル
追加情報

チャンネル情報＋ピーク情報
クロマトグラム名 fuse589-CH1
サンプル名
チャンネル名 CH1
データ取り込み間隔 500 [msec]
波形処理メソッド （マニュアル）
数値計算式
判定式

#	ピーク名	CH	tR [min]	面積 [µV·sec]	高さ [µV]	面積%	高さ%	定量値	NTP	分離度	シンメトリー係数	警告
1	Unknown	1	43.842	23401116	357825	99.749	99.633	N/A	10564	4.574	2.012	
2	Unknown	1	50.575	58864	1317	0.251	0.367	N/A	26366	N/A	0.979	

Procedure for preparation of 8j

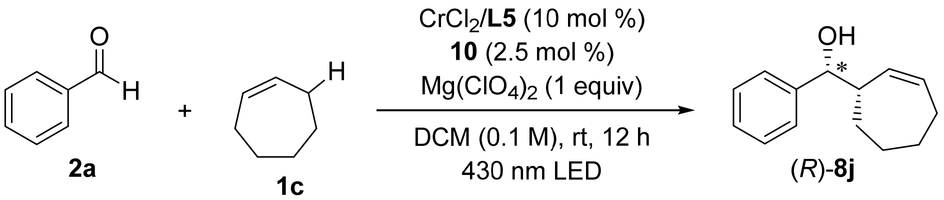

$CrCl_2$ (3.0 mg, 0.025 mmol, 10 mol %) and **L5** (9.0 mg, 0.025 mmol, 10 mol %) were dissolved in degassed CH_2Cl_2 (2.5 mL) in a screw-capped vial under argon atmosphere and the mixture was stirred for 1 hour at room temperature. Cyclohexene **1c** (584 µL, 5.0 mmol, 20 eq), $Mg(ClO_4)_2$ (55.8 mg, 0.25 mmol, 100 mol %), benzaldehyde **2a** (25.5 µL, 0.25 mmol, 1 eq) and photocatalyst **10** (2.5 mg, 0.00625 mmol, 2.5 mol %) were added to the reaction mixture. The vial was subjected to 430 nm LED irradiation for 12 hours under temperature control (ca. 27–29 ºC). Then, the reaction mixture was passed through a pad of silica gel with CH_2Cl_2 elution. After evaporation, diastereomeric ratio was determined by NMR analysis (68%, >20/1). The residue was purified by silica gel flash column chromatography (CH_2Cl_2/hexane = 1/2 to 2/1, v/v) to afford (*R*)-**8a** as colorless oil (28.4 mg, 56%).

(*R*)-((*S*)-cyclohept-2-en-1-yl)(phenyl)methanol (8j).

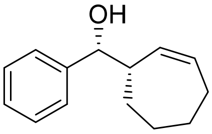

(*R*)-**8j**

All the spectroscopic data matches with the previously reported data.[9]

^{1}H NMR ($CDCl_3$, 500 MHz) δ 1.21-1.40 (m, 2H), 1.48-1.75 (m, 2H), 1.85-2.24 (m, 5H), 2.62-2.71 (m, 1H), 4.66 (d, *J* = 7.3 Hz, 1H), 5.53 (dd, *J* = 3.2, 14.5 Hz, 1H), 5.73-5.83 (m, 1H), 7.25-7.39 (m, 5H); ^{13}C NMR ($CDCl_3$, 125 MHz) δ 26.7, 28.1, 28.6, 30.0, 46.9, 77.4, 126.7, 127.5, 128.3, 132.4, 133.5, 143.2; HPLC (chiral column: CHIRALPAK IA3; solvent: hexane/2-propanol = 200/1; flow rate: 1.0 mL/min; detection: at 210 nm): t_R = 32.8 min (minor) and 38.4 min (major); $[\alpha]_D^{20}$ = +84 (*c* = 0.77, $CHCl_3$, for 99% ee sample).

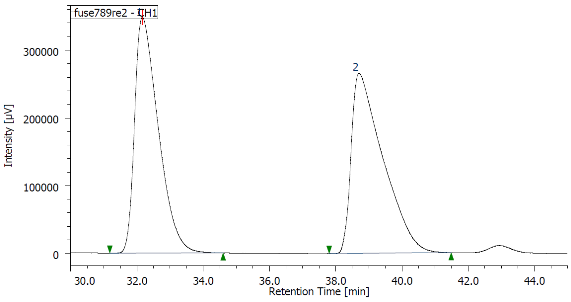

クロマトグラム情報
ユーザー名 a
更新日時 2018/10/01 13:53:26
コメント IA3 Hex IPA 200 1
HPLC システム名 HPLC-2
測定日 2018/10/01 13:07:38
注入量 10.00 [µL]
サンプル# 1
プロジェクト名 for you2
取込時間 90.0 [min]
測定シーケンス fuse789re2
コントロールメソッド Morita_1.0ml_210nm
ピーク ID テーブル
検量線テーブル
追加情報

チャンネル情報＋ピーク情報
クロマトグラム名 fuse789re2-CH1
サンプル名
チャンネル名 CH1
データ取り込み間隔 500 [msec]
波形処理メソッド （マニュアル）
数値計算式
判定式

#	ピーク名	CH	tR [min]	面積 [µV·sec]	高さ [µV]	面積%	高さ%	定量値	NTP	分離度	シンメトリー係数	警告
1	Unknown	1	32.167	17322583	348106	49.448	56.682	N/A	9837	4.310	1.714	
2	Unknown	1	38.700	17709163	266034	50.552	43.318	N/A	7891	N/A	2.286	

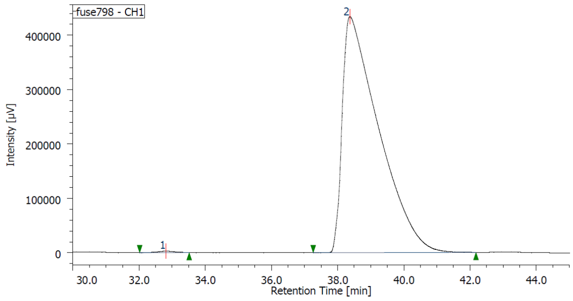

クロマトグラム情報
ユーザー名 a
更新日時 2018/10/01 15:16:11
コメント IA3 Hex IPA 200 1
HPLC システム名 HPLC-2
測定日 2018/10/01 14:27:14
注入量 10.00 [µL]
サンプル# 1
プロジェクト名 for you2
取込時間 90.0 [min]
測定シーケンス fuse798
コントロールメソッド Morita_1.0ml_210nm
ピーク ID テーブル
検量線テーブル
追加情報

チャンネル情報＋ピーク情報
クロマトグラム名 fuse798-CH1
サンプル名
チャンネル名 CH1
データ取り込み間隔 500 [msec]
波形処理メソッド （マニュアル）
数値計算式
判定式

#	ピーク名	CH	tR [min]	面積 [µV·sec]	高さ [µV]	面積%	高さ%	定量値	NTP	分離度	シンメトリー係数	警告
1	Unknown	1	32.808	105664	2784	0.317	0.638	N/A	17244	3.714	0.988	
2	Unknown	1	38.367	33190188	433694	99.683	99.362	N/A	5879	N/A	2.791	

Procedure for preparation of 8l and 8v

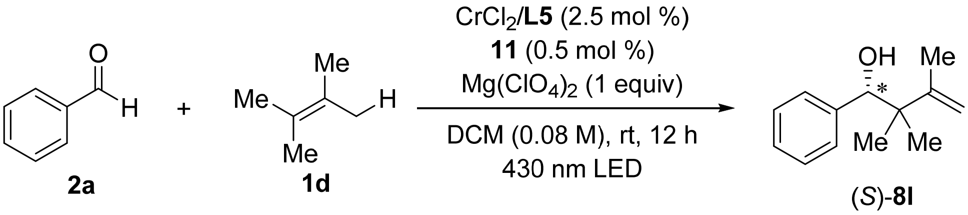

$CrCl_2$ (1.5 mg, 0.0125 mmol, 2.5 mol %) and **L5** (4.5 mg, 0.0125 mmol, 2.5 mol %) were dissolved in degassed CH_2Cl_2 (4 mL) in a screw-capped vial under argon atmosphere and the mixture was stirred for 1 hour at room temperature. 2,3-Dimethyl-2-butene **1d** (106 µL, 1.0 mmol, 2 eq), $Mg(ClO_4)_2$ (112 mg, 0.5 mmol, 100 mol %), benzaldehyde **2a** (51 µL, 0.5 mmol, 1 eq) and photocatalyst **11** (1.0 mg, 0.0025 mmol, 0.5 mol %) were added to the reaction mixture. The vial was subjected to 430 nm LED irradiation for 12 hours under temperature control (ca. 27–29 ºC). Then, the reaction mixture was directly loaded to silica gel and purified by flash column chromatography (CH_2Cl_2/hexane = 1/10 to 1/0, v/v) to afford (*R*)-**8l** as colorless oil (82.1 mg, 87%).

(*S*)-2,2,3-trimethyl-1-phenylbut-3-en-1-ol (8l).

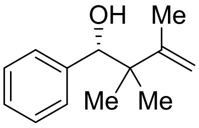

(S)-**8l**

All the spectroscopic data matches with the previously reported data.[10]

^{1}H NMR ($CDCl_3$, 500 MHz) δ 0.94 (s, 3H), 0.99 (s, 3H), 1.87 (s, 3H), 2.08 (d, J = 1.7 Hz, 1H), 4.63 (s, 1H), 4.97 (s, 1H), 5.04 (s, 1H), 7.18-7.36 (m, 5H); ^{13}C NMR ($CDCl_3$, 125 MHz) δ 19.8, 20.1, 24.2, 44.5, 77.4, 113.2, 127.2, 127.4, 127.9, 140.2, 150.6; HPLC (chiral column: CHIRALPAK IA3; solvent: hexane/2-propanol = 100/1; flow rate: 1.0 mL/min; detection: at 210 nm): t_R = 11.1 min (minor) and 13.5 min (major); $[\alpha]_D^{20}$ = +99 (c = 0.24, $CHCl_3$, for 88% ee sample).

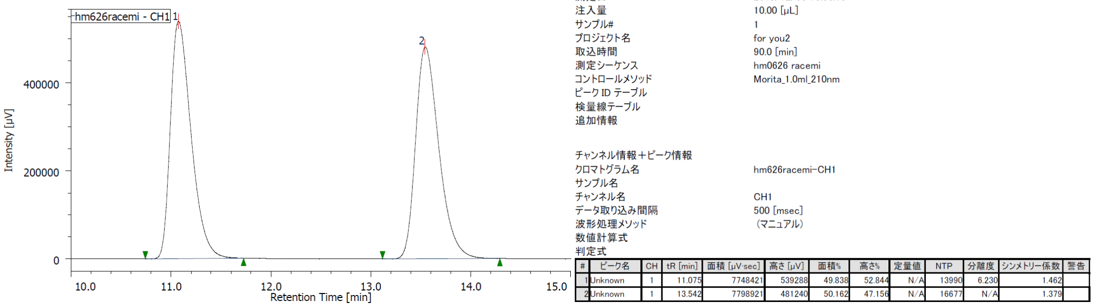

クロマトグラム情報
ユーザー名 a
更新日時 2018/12/03 13:55:23
コメント IA3 Hex IPA 100 1
HPLC システム名 HPLC-1
測定日 2018/12/03 13:36:16
注入量 10.00 [µL]
サンプル# 1
プロジェクト名 for you2
取込時間 90.0 [min]
測定シーケンス hm0626 racemi
コントロールメソッド Morita_1.0ml_210nm
ピーク ID テーブル
検量線テーブル
追加情報

チャンネル情報＋ピーク情報
クロマトグラム名 hm626racemi-CH1
サンプル名
チャンネル名 CH1
データ取り込み間隔 500 [msec]
波形処理メソッド （マニュアル）
数値計算式
判定式

#	ピーク名	CH	tR [min]	面積 [µV·sec]	高さ [µV]	面積%	高さ%	定量値	NTP	分離度	シンメトリー係数	警告
1	Unknown	1	11.075	7748421	539288	49.838	52.844	N/A	13990	6.230	1.462	
2	Unknown	1	13.542	7798921	481240	50.162	47.156	N/A	16677	N/A	1.379	

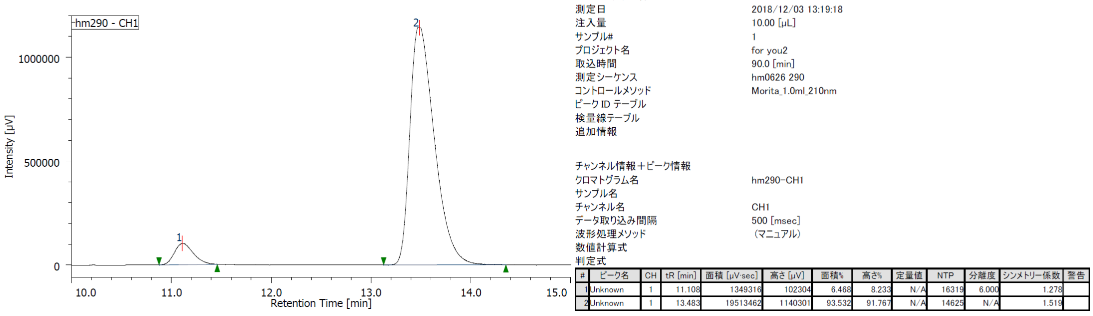

クロマトグラム情報
ユーザー名 a
更新日時 2018/12/03 13:35:51
コメント IA3 Hex IPA 100 1
HPLC システム名 HPLC-1
測定日 2018/12/03 13:19:18
注入量 10.00 [µL]
サンプル# 1
プロジェクト名 for you2
取込時間 90.0 [min]
測定シーケンス hm0626 290
コントロールメソッド Morita_1.0ml_210nm
ピーク ID テーブル
検量線テーブル
追加情報

チャンネル情報＋ピーク情報
クロマトグラム名 hm290-CH1
サンプル名
チャンネル名 CH1
データ取り込み間隔 500 [msec]
波形処理メソッド （マニュアル）
数値計算式
判定式

#	ピーク名	CH	tR [min]	面積 [µV·sec]	高さ [µV]	面積%	高さ%	定量値	NTP	分離度	シンメトリー係数	警告
1	Unknown	1	11.108	1349316	102304	6.468	8.233	N/A	16319	6.000	1.278	
2	Unknown	1	13.483	19513462	1140301	93.532	91.767	N/A	14625	N/A	1.519	

(R)-2,2-dimethyl-1-phenylbut-3-en-1-ol (8v).

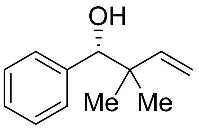

(R)-**8v**

Prepared according to the general procedure, then the reaction mixture was passed through a pad of silica gel with CH_2Cl_2. After evaporation, regioisomeric ratio was determined by NMR analysis (1.9/1 rr). The residue was purified by silica gel flash column chromatography (CH_2Cl_2/hexane = 1/10 to 1/0, v/v) to afford (R)-**8v**

as colorless oil along with the inseparable mixture of stereoisomers (**8w**) (42.8 mg, 97%). All the spectroscopic data matches with the previously reported data.[11] The following spectroscopic data were collected with a pure sample obtained by preparative TLC.

^{1}H NMR ($CDCl_3$, 500 MHz) δ 0.88 (s, 3H), 0.93 (s, 3H), 2.01 (s, 1H), 4.33 (s, 1H), 4.99 (dd, J = 1.4, 17.9 Hz, 1H), 5.05 (dd, J = 1.4, 11.0 Hz, 1H), 5.83 (dd, J = 11.0, 17.9 Hz, 1H), 7.12-7.26 (m, 5H); ^{13}C NMR ($CDCl_3$, 125 MHz) δ 20.1, 24.4, 42.2, 80.6, 113.8, 127.4, 127.4, 127.7, 140.7, 145.0; HPLC (chiral column: CHIRALPAK IE3; solvent: hexane/2-propanol = 500/1; flow rate: 1.0 mL/min; detection: at 210 nm): t_R = 27.3 min (minor) and 34.2 min (major); $[\alpha]_D^{20}$ = +111.5 (c = 1.23, $CHCl_3$, for 83% ee sample).

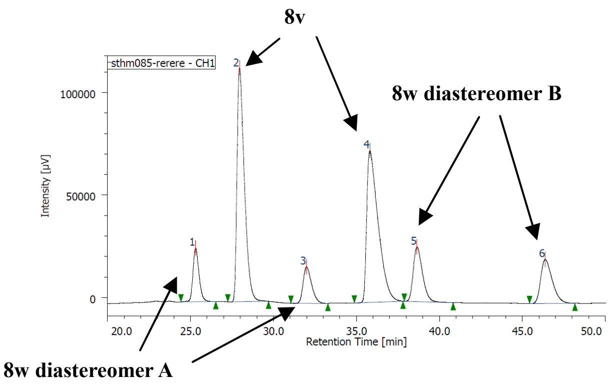

クロマトグラム情報	
ユーザー名	a
更新日時	2018/11/29 16:55:00
コメント	IE3 Hex IPA 500 1
HPLC システム名	HPLC-1
測定日	2018/11/29 15:57:05
注入量	10.00 [µL]
サンプル#	1
プロジェクト名	for you2
取込時間	90.0 [min]
測定シーケンス	st-data-hex-ipa-500-1-IE3-rerere
コントロールメソッド	Morita_1.0ml_210nm
ピーク ID テーブル	
検量線テーブル	
追加情報	
チャンネル情報＋ピーク情報	
クロマトグラム名	sthm085-rerere-CH1
サンプル名	
チャンネル名	CH1
データ取り込み間隔	500 [msec]
波形処理メソッド	（マニュアル）
数値計算式	
判定式	

#	ピーク名	CH	tR [min]	面積 [µV·sec]	高さ [µV]	面積%	高さ%	定量値	NTP	分離度	シンメトリー係数	警告
1	Unknown	1	25.300	663848	26122	6.365	9.334	N/A	24361	3.676	1.278	
2	Unknown	1	27.950	3521192	114106	33.763	40.773	N/A	19658	4.685	1.723	
3	Unknown	1	31.983	628204	17683	6.024	6.319	N/A	18960	3.520	1.391	
4	Unknown	1	35.800	3538154	73896	33.926	26.405	N/A	13222	2.525	2.240	
5	Unknown	1	38.642	1036199	26610	9.936	9.508	N/A	23367	6.808	1.381	
6	Unknown	1	46.375	1041446	21442	9.986	7.662	N/A	21444	N/A	1.428	

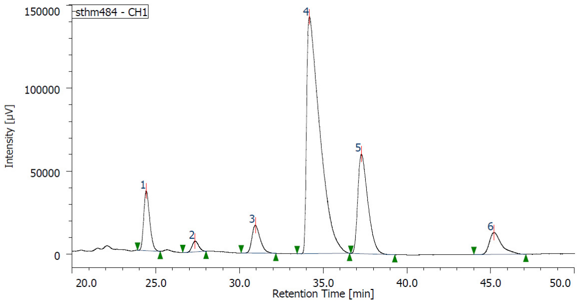

クロマトグラム情報	
ユーザー名	a
更新日時	2018/11/29 16:54:59
コメント	IE3 Hex IPA 500 1
HPLC システム名	HPLC-1
測定日	2018/11/29 13:14:06
注入量	10.00 [µL]
サンプル#	1
プロジェクト名	for you2
取込時間	90.0 [min]
測定シーケンス	st-data-hex-ipa-500-1-IE3
コントロールメソッド	Morita_1.0ml_210nm
ピーク ID テーブル	
検量線テーブル	
追加情報	
チャンネル情報＋ピーク情報	
クロマトグラム名	sthm484-CH1
サンプル名	
チャンネル名	CH1
データ取り込み間隔	500 [msec]
波形処理メソッド	（マニュアル）
数値計算式	
判定式	

#	ピーク名	CH	tR [min]	面積 [µV·sec]	高さ [µV]	面積%	高さ%	定量値	NTP	分離度	シンメトリー係数	警告
1	Unknown	1	24.425	889822	36031	7.023	13.088	N/A	22744	4.247	1.292	
2	Unknown	1	27.342	175879	6465	1.388	2.348	N/A	22476	4.486	1.180	
3	Unknown	1	30.942	572707	16815	4.520	6.108	N/A	19789	2.817	1.379	
4	Unknown	1	34.167	7850949	142704	61.963	51.834	N/A	9320	2.488	3.229	
5	Unknown	1	37.275	2508996	60055	19.802	21.814	N/A	18718	6.876	1.616	
6	Unknown	1	45.192	672092	13238	5.304	4.808	N/A	21985	N/A	1.692	

Procedure for preparation of 8m and 8p

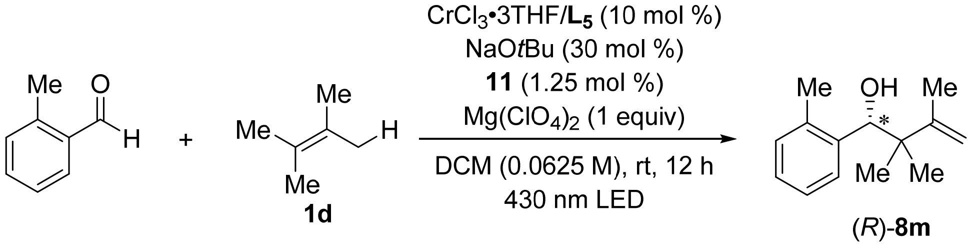

$CrCl_3$•3THF (9.4 mg, 0.025 mmol, 10 mol %), NaOtBu (7.2 mg, 0.075 mmol, 30 mol %) and **L5** (9.0 mg, 0.025 mmol, 10 mol %) were dissolved in degassed CH_2Cl_2 (4 mL) in a screw-capped vial under argon

atmosphere and the mixture was stirred for 1 hour at room temperature. 2,3-Dimethyl-2-butene **1d** (540 µL, 5.0 mmol, 20 eq), $Mg(ClO_4)_2$ (55.8 mg, 0.25 mmol, 100 mol %), *o*-tolaldehyde (29 µL, 0.25 mmol, 1 eq) and photocatalyst **11** (1.3 mg, 0.03125 mmol, 1.25 mol %) were added to the reaction mixture. The vial was subjected to 430 nm LED irradiation for 12 hours under temperature control (ca. 27–29 ºC). Then, the reaction mixture was directly loaded to silica gel and purified by flash column chromatography (CH_2Cl_2/hexane = 1/10 to 1/0, v/v) to afford (*R*)-**8m** as colorless oil (51.1 mg, 50%).

(*R*)-2,2,3-trimethyl-1-(*o*-tolyl)but-3-en-1-ol (8m).

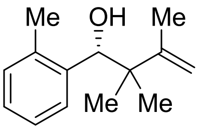

(*R*)-**8m**

IR (neat) ν 3466, 3090, 2972, 1633, 1607, 1463, 1377, 1150, 1049, 892, 790, 734 cm^{-1}; ^1H NMR ($CDCl_3$, 500 MHz) δ 0.93 (s, 3H), 1.10 (s, 3H), 1.90 (s, 3H), 2.00 (d, J = 1.7 Hz, 1H), 2.38 (s, 3H), 4.90-5.10 (m, 2H), 5.05 (s, 1H), 7.10-7.25 (m, 3H), 7.50 (dd, J = 1.7, 7.4 Hz, 1H); ^{13}C NMR ($CDCl_3$, 125 MHz) δ 20.1, 20.4, 20.5, 23.8, 45.8, 72.2, 113.3, 125.3, 127.1, 128.3, 130.1,136.1, 138.6, 150.9; LRMS (ESI): *m*/*z* 227 [M+Na$^+$]; HRMS (ESI): *m*/*z* calculated for $C_{14}H_{20}NaO^+$ [M+Na$^+$]: 227.1406, found: 227.1395; HPLC (chiral column: CHIRALPAK IA3; solvent: hexane/2-propanol = 100/1; flow rate: 1.0 mL/min; detection: at 210 nm): t_R = 9.7 min (major) and 10.7 min (minor); $[\alpha]_D^{20}$ = +110 (c = 1.05, $CHCl_3$, for 96% ee sample).

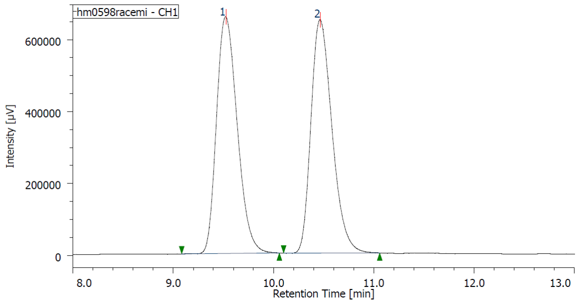

クロマトグラム情報
ユーザー名 a
更新日時 2018/10/17 23:27:56
コメント IA3 Hex IPA 100 1
HPLC システム名 HPLC-1
測定日 2018/10/17 22:43:47
注入量 10.00 [µL]
サンプル# 1
プロジェクト名 for you2
取込時間 90.0 [min]
測定シーケンス hm0598racemi
コントロールメソッド Morita_1.0ml_210nm
ピーク ID テーブル
検量線テーブル
追加情報

チャンネル情報＋ピーク情報
クロマトグラム名 hm0598racemi-CH1
サンプル名
チャンネル名 CH1
データ取り込み間隔 500 [msec]
波形処理メソッド （マニュアル）
数値計算式
判定式

#	ピーク名	CH	tR [min]	面積 [µV·sec]	高さ [µV]	面積%	高さ%	定量値	NTP	分離度	シンメトリー係数	警告
1	Unknown	1	9.525	9458527	658152	49.945	50.376	N/A	9996	2.471	1.240	
2	Unknown	1	10.467	9479392	648329	50.055	49.624	N/A	11935	N/A	1.285	

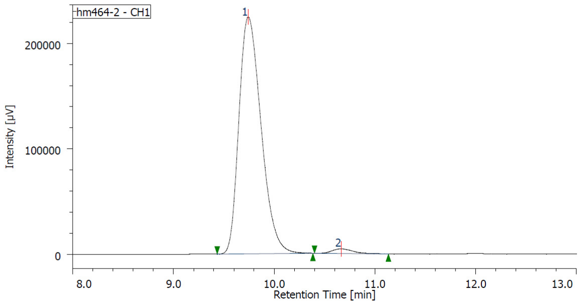

クロマトグラム情報
ユーザー名 a
更新日時 2018/09/12 0:11:54
コメント IA3 Hex IPA 100 1
HPLC システム名 HPLC-1
測定日 2018/09/11 22:56:32
注入量 10.00 [µL]
サンプル# 1
プロジェクト名 for you2
取込時間 90.0 [min]
測定シーケンス hm0464-2
コントロールメソッド Morita_1.0ml_210nm
ピーク ID テーブル
検量線テーブル
追加情報

チャンネル情報＋ピーク情報
クロマトグラム名 hm464-2-CH1
サンプル名
チャンネル名 CH1
データ取り込み間隔 500 [msec]
波形処理メソッド （マニュアル）
数値計算式
判定式

#	ピーク名	CH	tR [min]	面積 [µV·sec]	高さ [µV]	面積%	高さ%	定量値	NTP	分離度	シンメトリー係数	警告
1	Unknown	1	9.742	3436238	224758	98.131	98.051	N/A	9306	2.369	1.329	
2	Unknown	1	10.667	65429	4467	1.869	1.949	N/A	12662	N/A	1.405	

(*S*)-1-(4-methoxyphenyl)-2,2,3-trimethylbut-3-en-1-ol (8p).

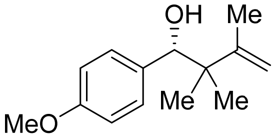

(*S*)-**8p**

Prepared according to the general procedure, then the crude material was purified by silica gel flash column chromatography (AcOEt/hexane = 1/10 to 1/1, v/v) to afford (*S*)-**8p** as colorless oil (21.5 mg, 39%). All the spectroscopic data matches with the previously reported data.[10]

^{1}H NMR ($CDCl_3$, 500 MHz) δ 0.92 (s, 3H), 0.98 (s, 3H), 1.86 (s, 3H), 2.03 (d, J = 1.7 Hz, 1H), 3.81 (s, 3H), 4.59 (s, 1H), 4.96 (s, 1H), 5.02 (s, 1H), 6.83-6.88 (m, 2H), 7.23-7.27 (m, 2H); ^{13}C NMR ($CDCl_3$, 125 MHz) δ 19.8, 20.0, 24.2, 44.6, 55.2, 77.1, 112.8, 113.1, 129.0, 132.3, 150.7, 158.8; HPLC (chiral column: CHIRALPAK IA3; solvent: hexane/2-propanol = 200/1; flow rate: 1.0 mL/min; detection: at 210 nm): t_R = 27.2 min (minor) and 28.8 min (major); $[\alpha]_D^{20}$ = +138 (c = 0.49, $CHCl_3$, for 96% ee sample).

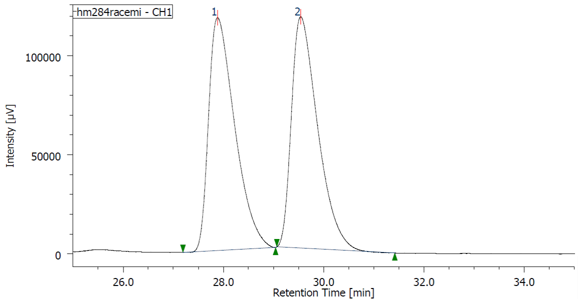

クロマトグラム情報
ユーザー名 a
更新日時 2018/10/17 23:27:56
コメント IA3 Hex IPA 200 1
HPLC システム名 HPLC-1
測定日 2018/10/17 19:58:24
注入量 10.00 [µL]
サンプル# 1
プロジェクト名 for you2
取込時間 90.0 [min]
測定シーケンス hm0284 racemi2
コントロールメソッド Morita_1.0ml_210nm
ピーク ID テーブル
検量線テーブル
追加情報

チャンネル情報＋ピーク情報
クロマトグラム名 hm284racemi-CH1
サンプル名
チャンネル名 CH1
データ取り込み間隔 500 [msec]
波形処理メソッド （マニュアル）
数値計算式
判定式

#	ピーク名	CH	tR [min]	面積 [µV·sec]	高さ [µV]	面積%	高さ%	定量値	NTP	分離度	シンメトリー係数	警告
1	Unknown	1	27.883	4261053	117442	50.050	50.169	N/A	13740	1.750	1.691	
2	Unknown	1	29.542	4252623	116651	49.950	49.831	N/A	15495	N/A	1.658	

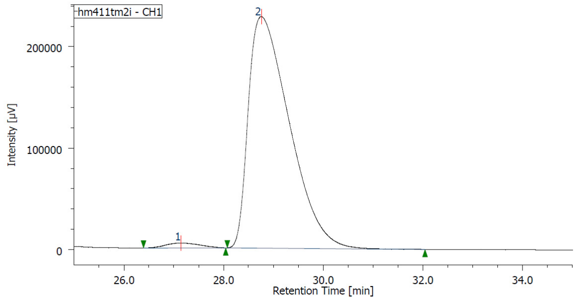

クロマトグラム情報
ユーザー名 a
更新日時 2018/10/17 23:27:56
コメント IA3 Hex IPA 200 1
HPLC システム名 HPLC-1
測定日 2018/10/17 21:19:18
注入量 10.00 [µL]
サンプル# 1
プロジェクト名 for you2
取込時間 90.0 [min]
測定シーケンス hm0411tm2
コントロールメソッド Morita_1.0ml_210nm
ピーク ID テーブル
検量線テーブル
追加情報

チャンネル情報＋ピーク情報
クロマトグラム名 hm411tm2i-CH1
サンプル名
チャンネル名 CH1
データ取り込み間隔 500 [msec]
波形処理メソッド （マニュアル）
数値計算式
判定式

#	ピーク名	CH	tR [min]	面積 [µV·sec]	高さ [µV]	面積%	高さ%	定量値	NTP	分離度	シンメトリー係数	警告
1	Unknown	1	27.150	237435	4956	1.785	2.126	N/A	6624	1.127	1.131	
2	Unknown	1	28.758	13066026	228098	98.215	97.874	N/A	5680	N/A	1.884	

Procedure for preparation of 8n and 8o

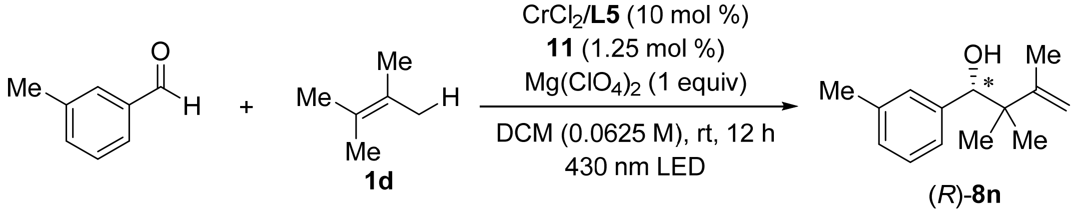

(*R*)-**8n**

$CrCl_2$ (3.0 mg, 0.025 mmol, 10 mol %) and **L5** (9.0 mg, 0.025 mmol, 10 mol %) were dissolved in degassed CH_2Cl_2 (4 mL) in a screw-capped vial under argon atmosphere and the mixture was stirred for 1 hour at room temperature. 2,3-Dimethyl-2-butene **1d** (148 μL, 1.25 mmol, 5 eq), $Mg(ClO_4)_2$ (55.8 mg, 0.25 mmol, 100 mol %), *m*-tolaldehyde (29 μL, 0.25 mmol, 1 eq) and photocatalyst **11** (1.3 mg, 0.003125 mmol, 1.25 mol %) were added to the reaction mixture. The vial was subjected to 430 nm LED irradiation for 12 hours under temperature control (ca. 27–29 ºC). Then, the reaction mixture was directly loaded to silica gel and purified by flash column chromatography (CH_2Cl_2/hexane = 1/10 to 1/0, v/v) to afford (*S*)-**8n** as colorless oil (43.2 mg, 85%).

(*S*)-2,2,3-trimethyl-1-(*m*-tolyl)but-3-en-1-ol (8n).

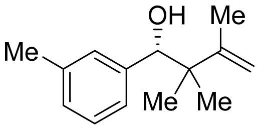

(*R*)-**8n**

IR (neat) ν 3468, 2974, 1715, 1632, 1461, 1378, 1181, 1039, 1007, 893, 764, 738 cm^{-1}; ^1H NMR ($CDCl_3$, 500 MHz) δ 0.94 (s, 3H), 0.99 (s, 3H), 1.87 (s, 3H), 2.05 (s, 1H), 2.36 (s, 3H), 4.60 (s, 1H), 4.97 (d, *J* = 1.1 Hz, 1H), 5.03 (d, *J* = 1.1 Hz, 1H), 7.08 (d, *J* = 7.4 Hz, 1H), 7.12 (d, *J* = 7.4 Hz, 1H), 7.16 (s, 1H), 7.20 (dd, *J* = 7.4, 7.4 Hz, 1H); ^{13}C NMR ($CDCl_3$, 125 MHz) δ 19.8, 20.0, 21.5, 24.3, 44.4, 77.4, 113.1, 125.1, 127.2, 128.0, 128.6, 136.8, 140.2, 150.7; LRMS (ESI): *m*/*z* 227 [M+Na$^+$]; HRMS (ESI): *m*/*z* calculated for $C_{14}H_{20}NaO^+$ [M+Na$^+$]: 227.1406, found: 227.1399; HPLC (chiral column: CHIRALPAK IBN3; solvent: hexane/2-propanol = 200/1; flow rate: 1.0 mL/min; detection: at 210 nm): t_R = 16.2 min (minor) and 21.9 min (major); $[\alpha]_D^{20}$ = +103 (*c* = 0.9, $CHCl_3$, for 90% ee sample).

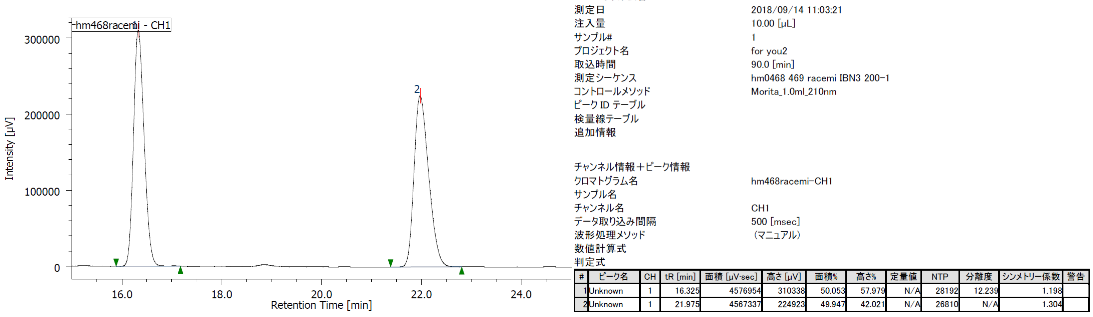

クロマトグラム情報
ユーザー名 a
更新日時 2018/09/14 12:44:27
コメント IBN3 Hex IPA 200 1
HPLC システム名 HPLC-1
測定日 2018/09/14 11:03:21
注入量 10.00 [μL]
サンプル# 1
プロジェクト名 for you2
取込時間 90.0 [min]
測定シーケンス hm0468 469 racemi IBN3 200-1
コントロールメソッド Morita_1.0ml_210nm
ピーク ID テーブル
検量線テーブル
追加情報

チャンネル情報＋ピーク情報
クロマトグラム名 hm468racemi-CH1
サンプル名
チャンネル名 CH1
データ取り込み間隔 500 [msec]
波形処理メソッド （マニュアル）
数値計算式
判定式

#	ピーク名	CH	tR [min]	面積 [μV·sec]	高さ [μV]	面積%	高さ%	定量値	NTP	分離度	シンメトリー係数	警告
1	Unknown	1	16.325	4576954	310338	50.053	57.979	N/A	28192	12.239	1.198	
2	Unknown	1	21.975	4567337	224923	49.947	42.021	N/A	26810	N/A	1.304	

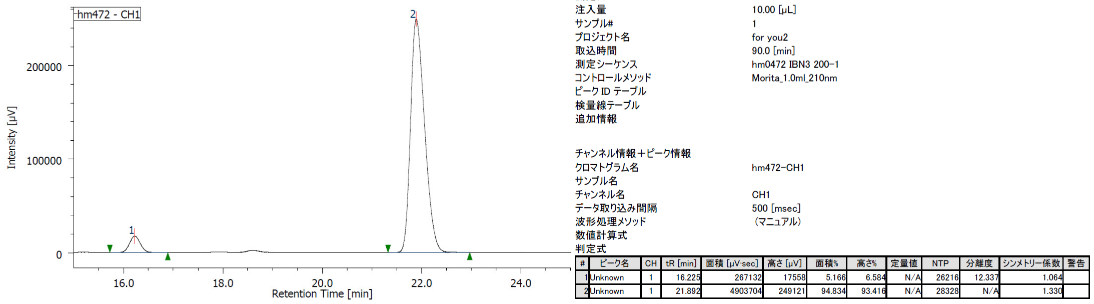

クロマトグラム情報	
ユーザー名	a
更新日時	2018/09/14 12:44:27
コメント	IBN3 Hex IPA 200 1
HPLC システム名	HPLC-1
測定日	2018/09/14 12:14:14
注入量	10.00 [µL]
サンプル#	1
プロジェクト名	for you2
取込時間	90.0 [min]
測定シーケンス	hm0472 IBN3 200-1
コントロールメソッド	Morita_1.0ml_210nm
ピーク ID テーブル	
検量線テーブル	
追加情報	
チャンネル情報＋ピーク情報	
クロマトグラム名	hm472-CH1
サンプル名	
チャンネル名	CH1
データ取り込み間隔	500 [msec]
波形処理メソッド	（マニュアル）
数値計算式	
判定式	

#	ピーク名	CH	tR [min]	面積 [µV·sec]	高さ [µV]	面積%	高さ%	定量値	NTP	分離度	シンメトリー係数	警告
1	Unknown	1	16.225	267132	17558	5.166	6.584	N/A	26216	12.337	1.064	
2	Unknown	1	21.892	4903704	249121	94.834	93.416	N/A	28328	N/A	1.330	

(*S*)-2,2,3-trimethyl-1-(*p*-tolyl)but-3-en-1-ol (8o).

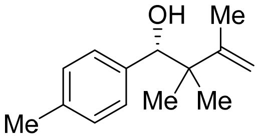

(*S*)-**8o**

Prepared according to the general procedure, then the crude material was purified by silica gel flash column chromatography (CH_2Cl_2/hexane = 1/10 to 1/0, v/v) to afford (*S*)-**8o** as colorless oil (49.4 mg, 97%). All the spectroscopic data matches with the previously reported data.[12]

^{1}H NMR ($CDCl_3$, 500 MHz) δ 0.93 (s, 3H), 0.98 (s, 3H), 1.86 (s, 3H), 2.03 (d, J = 1.7 Hz, 1H), 2.34 (s, 3H), 4.60 (s, 1H), 4.96 (d, J = 1.1 Hz, 1H), 5.02 (d, J = 1.1 Hz, 1H), 7.12 (d, J = 8.0 Hz, 2H), 7.22 (d, J = 8.0 Hz, 2H); ^{13}C NMR ($CDCl_3$, 125 MHz) δ 19.8, 20.1, 21.1, 24.2, 44.4, 77.3, 113.0, 127.8, 128.1, 136.8, 137.3, 150.7; HPLC (chiral column: CHIRALPAK IA3; solvent: hexane/2-propanol = 200/1; flow rate: 1.0 mL/min; detection: at 210 nm): t_R = 15.7 min (minor) and 17.1 min (major); $[\alpha]_D^{20}$ = +134 (c = 1.76, $CHCl_3$, for 95% ee sample).

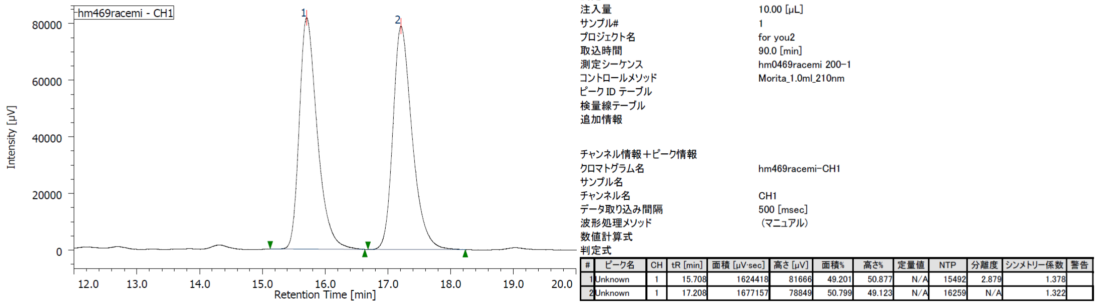

クロマトグラム情報	
ユーザー名	a
更新日時	2018/09/14 13:30:15
コメント	IA3 Hex IPA 200 1
HPLC システム名	HPLC-2
測定日	2018/09/14 12:39:41
注入量	10.00 [µL]
サンプル#	1
プロジェクト名	for you2
取込時間	90.0 [min]
測定シーケンス	hm0469racemi 200-1
コントロールメソッド	Morita_1.0ml_210nm
ピーク ID テーブル	
検量線テーブル	
追加情報	
チャンネル情報＋ピーク情報	
クロマトグラム名	hm469racemi-CH1
サンプル名	
チャンネル名	CH1
データ取り込み間隔	500 [msec]
波形処理メソッド	（マニュアル）
数値計算式	
判定式	

#	ピーク名	CH	tR [min]	面積 [µV·sec]	高さ [µV]	面積%	高さ%	定量値	NTP	分離度	シンメトリー係数	警告
1	Unknown	1	15.708	1624418	81666	49.201	50.877	N/A	15492	2.879	1.378	
2	Unknown	1	17.208	1677157	78849	50.799	49.123	N/A	16259	N/A	1.322	

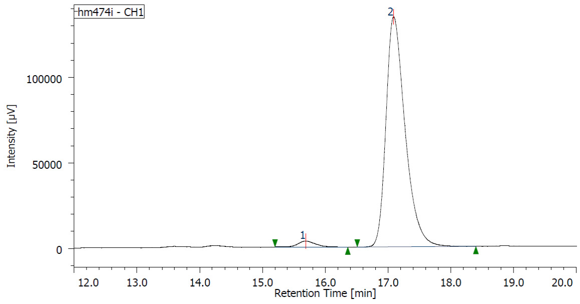

クロマトグラム情報	
ユーザー名	a
更新日時	2018/09/14 13:30:15
コメント	IA3 Hex IPA 200 1
HPLC システム名	HPLC-2
測定日	2018/09/14 13:00:54
注入量	10.00 [µL]
サンプル#	1
プロジェクト名	for you2
取込時間	90.0 [min]
測定シーケンス	hm0474 200-1
コントロールメソッド	Morita_1.0ml_210nm
ピーク ID テーブル	
検量線テーブル	
追加情報	

チャンネル情報＋ピーク情報	
クロマトグラム名	hm474i-CH1
サンプル名	
チャンネル名	CH1
データ取り込み間隔	500 [msec]
波形処理メソッド	（マニュアル）
数値計算式	
判定式	

#	ピーク名	CH	tR [min]	面積 [µV·sec]	高さ [µV]	面積%	高さ%	定量値	NTP	分離度	シンメトリー係数	警告
1	Unknown	1	15.692	68323	3457	2.337	2.507	N/A	15761	2.699	1.169	
2	Unknown	1	17.092	2855095	134465	97.663	97.493	N/A	16027	N/A	1.401	

Procedure for preparation of 8q

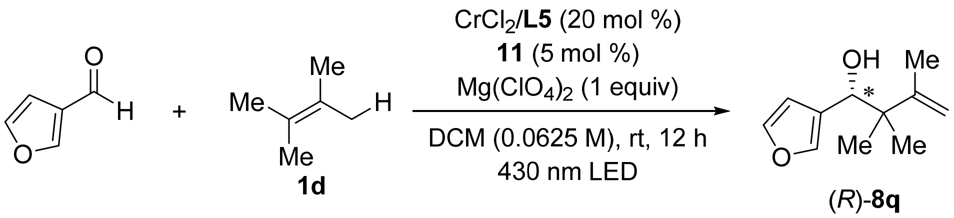

$CrCl_2$ (6.1 mg, 0.05 mmol, 20 mol %) and **L5** (17.9 mg, 0.05 mmol, 20 mol %) were dissolved in degassed CH_2Cl_2 (4 mL) in a screw-capped vial under argon atmosphere and the mixture was stirred for 1 hour at room temperature. 2,3-Dimethyl-2-butene **1d** (593 µL, 5.0 mmol, 20 eq), $Mg(ClO_4)_2$ (55.8 mg, 0.25 mmol, 100 mol %), 3-furaldehyde (21 µL, 0.25 mmol, 1 eq) and photocatalyst **11** (5.2 mg, 0.00125 mmol, 5 mol %) were added to the reaction mixture. The vial was subjected to 430 nm LED irradiation for 12 hours under temperature control (ca. 27–29 ºC). Then, the reaction mixture was directly loaded to silica gel and purified by flash column chromatography (CH_2Cl_2/hexane = 1/10 to 1/0, v/v) to afford (*R*)-**8q** as colorless oil (14.8 mg, 33%).

(*R*)-1-(furan-3-yl)-2,2,3-trimethylbut-3-en-1-ol (8q).

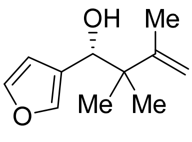

(*R*)-**8q**

IR (neat) ν 3464, 3091, 2971, 1716, 1635, 1501, 1460, 1377, 1261, 1163, 1023, 875, 798, 736 cm^{-1}; ^{1}H NMR ($CDCl_3$, 500 MHz) δ 0.98 (s, 3H), 1.05 (s, 3H), 1.83 (s, 3H), 1.93 (s, 1H), 4.61 (s, 1H), 4.95 (s, 1H), 5.01 (s, 1H), 6.39 (s, 1H), 7.35-7.39 (m, 2H); ^{13}C NMR ($CDCl_3$, 125 MHz) δ 19.8, 20.5, 23.7, 44.2, 71.3, 110.3, 113.1, 124.9, 140.3, 142.3, 150.3; LRMS (ESI): *m*/*z* 203 [M+Na$^+$]; HRMS (ESI): *m*/*z* calculated for $C_{11}H_{16}NaO_2^+$ [M+Na$^+$]: 203.1043, found: 203.1040; HPLC (chiral column: CHIRALPAK IBN3; solvent: hexane/2-propanol = 100/1; flow rate: 1.0 mL/min; detection: at 210 nm): t_R = 12.1 min (major) and 13.1 min (minor); $[\alpha]_D^{20}$ = +56 (*c* = 0.54, $CHCl_3$, for 93% ee sample).

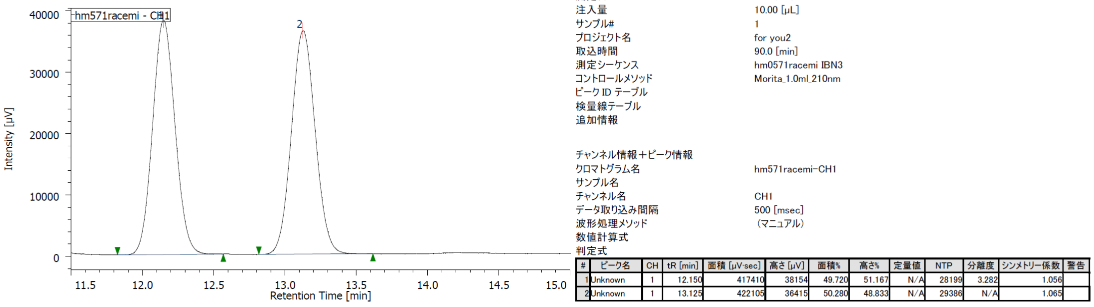

クロマトグラム情報
ユーザー名 a
更新日時 2018/10/08 20:37:34
コメント IBN3 Hex IPA 100 1
HPLC システム名 HPLC-1
測定日 2018/10/07 17:06:53
注入量 10.00 [µL]
サンプル# 1
プロジェクト名 for you2
取込時間 90.0 [min]
測定シーケンス hm0571racemi IBN3
コントロールメソッド Morita_1.0ml_210nm
ピーク ID テーブル
検量線テーブル
追加情報

チャンネル情報＋ピーク情報
クロマトグラム名 hm571racemi-CH1
サンプル名
チャンネル名 CH1
データ取り込み間隔 500 [msec]
波形処理メソッド （マニュアル）
数値計算式
判定式

#	ピーク名	CH	tR [min]	面積 [µV·sec]	高さ [µV]	面積%	高さ%	定量値	NTP	分離度	シンメトリー係数	警告
1	Unknown	1	12.150	417410	38154	49.720	51.167	N/A	28199	3.282	1.056	
2	Unknown	1	13.125	422105	36415	50.280	48.833	N/A	29386	N/A	1.065	

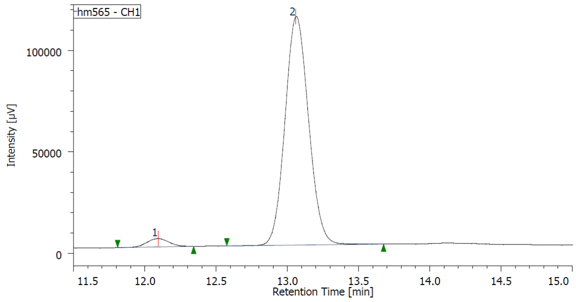

クロマトグラム情報
ユーザー名 a
更新日時 2018/10/08 20:37:34
コメント IBN3 Hex IPA 100 1
HPLC システム名 HPLC-1
測定日 2018/10/07 17:44:05
注入量 10.00 [µL]
サンプル# 1
プロジェクト名 for you2
取込時間 90.0 [min]
測定シーケンス hm0565 IBN3
コントロールメソッド Morita_1.0ml_210nm
ピーク ID テーブル
検量線テーブル
追加情報

チャンネル情報＋ピーク情報
クロマトグラム名 hm565-CH1
サンプル名
チャンネル名 CH1
データ取り込み間隔 500 [msec]
波形処理メソッド （マニュアル）
数値計算式
判定式

#	ピーク名	CH	tR [min]	面積 [µV·sec]	高さ [µV]	面積%	高さ%	定量値	NTP	分離度	シンメトリー係数	警告
1	Unknown	1	12.092	44103	4124	3.292	3.536	N/A	28812	3.297	1.034	
2	Unknown	1	13.058	1295406	112504	96.708	96.464	N/A	29717	N/A	1.112	

Procedure for preparation of 8r

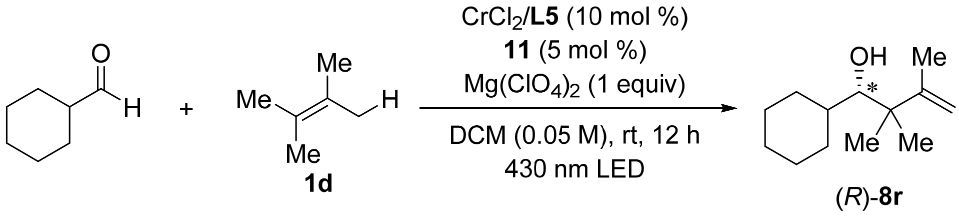

$CrCl_2$ (2.4 mg, 0.02 mmol, 20 mol %) and **L5** (7.2 mg, 0.02 mmol, 20 mol %) were dissolved in degassed 1,2-dichloroethane (4 mL) in a screw-capped vial under argon atmosphere and the mixture was stirred for 1 hour at room temperature. 2,3-Dimethyl-2-butene **1a** (119 µL, 1.0 mmol, 5 eq), $Mg(ClO_4)_2$ (44.6 mg, 0.2 mmol, 100 mol %), cyclohexanecarbaldehyde (24 µL, 0.2 mmol, 1 eq) and photocatalyst **11** (4.1 mg, 0.01 mmol, 5 mol %) were added to the reaction mixture. The vial was subjected to 430 nm LED irradiation for 12 hours under temperature control (ca. 27–29 ºC). Then, the reaction mixture was directly loaded to silica gel and purified by flash column chromatography (CH_2Cl_2/hexane = 1/10 to 1/0, v/v) to afford (*S*)-**8r** as colorless oil (35.5 mg, 90%).

(*S*)-1-cyclohexyl-2,2,3-trimethylbut-3-en-1-ol (8r).

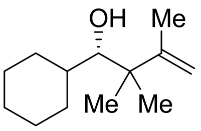

(*S*)-**8r**

All the spectroscopic data matches with the previously reported data.[10] Enantiomeric excess was determined after protection of **8r** by *p*-NO_2-benzoyl chloride.

^{1}H NMR ($CDCl_3$, 500 MHz) δ 1.06 (s, 3H), 1.08 (s, 3H), 1.19-1.37 (m, 6H), 1.43 (d, *J* = 4.6 Hz, 1H), 1.45-1.66 (m, 2H), 1.68-1.80 (m, 6H), 3.28 (dd, *J* = 2.9, 4.0 Hz, 1H), 4.83 (s, 1H), 4.87 (s, 1H); ^{13}C NMR ($CDCl_3$, 125 MHz) δ 20.0, 22.9, 23.2, 26.4, 26.4, 26.9, 28.0, 34.0, 39.1, 44.6, 79.5, 111.7, 151.7; HPLC (chiral column: CHIRALPAK IA3; solvent: hexane/2-propanol = 200/1; flow rate: 1.0 mL/min; detection: at 254 nm): t_R = 7.6 min (minor) and 8.8 min (major); $[\alpha]_D^{20}$ = –49 (*c* = 1.19, $CHCl_3$, for 99% ee sample).

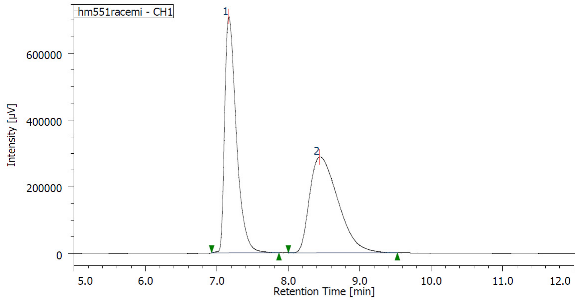

クロマトグラム情報	
ユーザー名	a
更新日時	2018/09/30 1:01:43
コメント	IA3 Hex IPA 200 1
HPLC システム名	HPLC-2
測定日	2018/09/29 12:26:07
注入量	10.00 [µL]
サンプル#	1
プロジェクト名	for you2
取込時間	90.0 [min]
測定シーケンス	hm0551racemi 557 558 200-1
コントロールメソッド	1ml254nm
ピーク ID テーブル	
検量線テーブル	
追加情報	
チャンネル情報＋ピーク情報	
クロマトグラム名	hm551racemi-CH1
サンプル名	
チャンネル名	CH1
データ取り込み間隔	500 [msec]
波形処理メソッド	(マニュアル)
数値計算式	
判定式	

#	ピーク名	CH	tR [min]	面積 [µV·sec]	高さ [µV]	面積%	高さ%	定量値	NTP	分離度	シンメトリー係数	警告
1	Unknown	1	7.167	8039357	708853	50.204	71.113	N/A	9844	2.484	1.739	
2	Unknown	1	8.442	7974122	287949	49.796	28.887	N/A	2080	N/A	1.615	

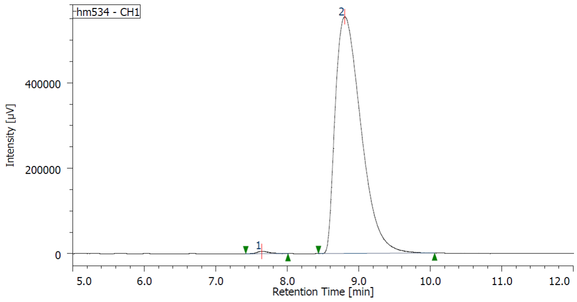

クロマトグラム情報	
ユーザー名	a
更新日時	2018/09/26 23:17:09
コメント	IA3 Hex IPA 200 1
HPLC システム名	HPLC-2
測定日	2018/09/26 20:56:32
注入量	10.00 [µL]
サンプル#	1
プロジェクト名	for you2
取込時間	90.0 [min]
測定シーケンス	hm0534 200-1
コントロールメソッド	1ml254nm
ピーク ID テーブル	
検量線テーブル	
追加情報	
チャンネル情報＋ピーク情報	
クロマトグラム名	hm534-CH1
サンプル名	
チャンネル名	CH1
データ取り込み間隔	500 [msec]
波形処理メソッド	(マニュアル)
数値計算式	
判定式	

#	ピーク名	CH	tR [min]	面積 [µV·sec]	高さ [µV]	面積%	高さ%	定量値	NTP	分離度	シンメトリー係数	警告
1	Unknown	1	7.642	68770	5797	0.493	1.035	N/A	10031	2.390	1.501	
2	Unknown	1	8.800	13880821	554307	99.507	98.965	N/A	2788	N/A	1.725	

Procedure for preparation of 8s-8u

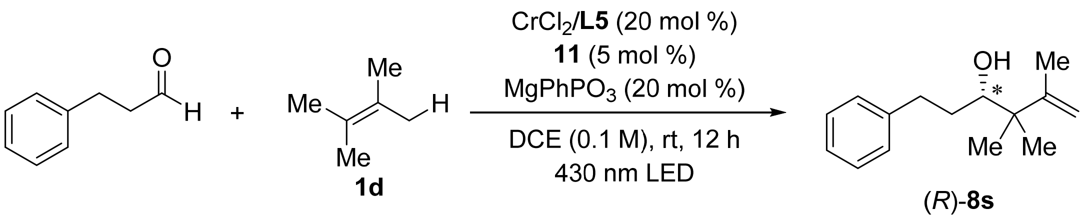

$CrCl_2$ (6.1 mg, 0.05 mmol, 20 mol %) and **L5** (17.9 mg, 0.05 mmol, 20 mol %) were dissolved in degassed

1,2-dichloroethane (2.5 mL) in a screw-capped vial under argon atmosphere and the mixture was stirred for 1 hour at room temperature. 2,3-Dimethyl-2-butene **1d** (593 μL, 5.0 mmol, 20 eq), $MgPhPO_3$ (9.0 mg, 0.05 mmol, 20 mol %), hydrocinnamaldehyde (33 μL, 0.25 mmol, 1 eq) and photocatalyst **11** (5.2 mg, 0.0125 mmol, 5 mol %) were added to the reaction mixture. The vial was subjected to 430 nm LED irradiation for 48 hours under temperature control (ca. 27–29 ºC). Then, the reaction mixture was directly loaded to silica gel and purified by flash column chromatography (CH_2Cl_2/hexane = 1/10 to 1/0, v/v) to afford (*S*)-**8s** as colorless oil (33.8 mg, 69%).

(*S*)-4,4,5-trimethyl-1-phenylhex-5-en-3-ol (8s).

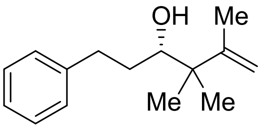

(*S*)-**8s**

IR (neat) ν 3479, 3087, 3027, 2970, 1634, 1603, 1496, 1455, 1377, 1302, 1067, 894, 748, 699 cm^{-1}; ^{1}H NMR ($CDCl_3$, 500 MHz) δ 0.99 (s, 3H), 1.04 (s, 3H), 1.50-1.81 (m, 6H), 2.55-2.66 (m, 1H), 2.88-2.99 (m, 1H), 3.50 (d, J = 10.3 Hz, 1H), 4.84 (s, 1H), 4.90 (s, 1H), 7.15-7.32 (m, 5H); ^{13}C NMR ($CDCl_3$, 125 MHz) δ 19.6, 21.5, 22.4, 33.0, 33.6, 43.7, 74.8, 112.1, 125.7, 128.3, 128.5, 142.5, 150.8; LRMS (DART): m/z 219 [M+H$^+$]; HRMS (DART): m/z calculated for $C_{15}H_{23}O^+$ [M+H$^+$]: 219.1743, found: 219.1753; HPLC (chiral column: CHIRALPAK IA3; solvent: hexane/2-propanol = 100/1; flow rate: 1.0 mL/min; detection: at 210 nm): t_R = 9.2 min (minor) and 10.5 min (major); $[\alpha]_D^{20}$ = +98 (c = 1.38, $CHCl_3$, for 86% ee sample).

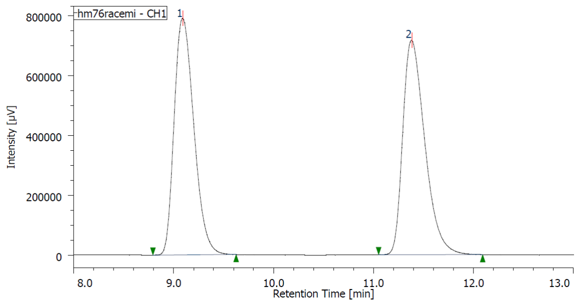

クロマトグラム情報

ユーザー名	a
更新日時	2018/12/03 12:17:45
コメント	IA3 Hex IPA 100 1
HPLC システム名	HPLC-1
測定日	2018/12/03 10:47:43
注入量	10.00 [μL]
サンプル#	1
プロジェクト名	for you2
取込時間	90.0 [min]
測定シーケンス	hm0626 290 76 547
コントロールメソッド	Morita_1.0ml_210nm
ピーク ID テーブル	
検量線テーブル	
追加情報	

チャンネル情報＋ピーク情報

クロマトグラム名	hm76racemi-CH1
サンプル名	
チャンネル名	CH1
データ取り込み間隔	500 [msec]
波形処理メソッド	（マニュアル）
数値計算式	
判定式	

#	ピーク名	CH	tR [min]	面積 [μV·sec]	高さ [μV]	面積%	高さ%	定量値	NTP	分離度	シンメトリー係数	警告
1	Unknown	1	9.092	10832123	790412	49.681	52.481	N/A	9965	6.018	1.286	
2	Unknown	1	11.383	10971109	715671	50.319	47.519	N/A	13002	N/A	1.432	

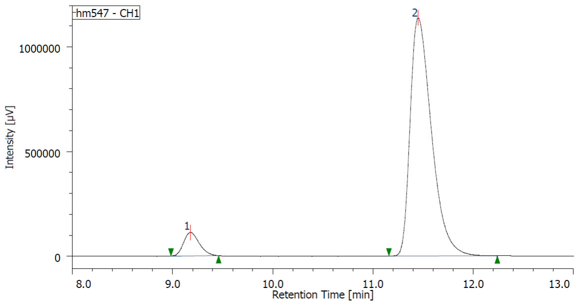

クロマトグラム情報

ユーザー名	a
更新日時	2018/12/03 13:02:46
コメント	IA3 Hex IPA 100 1
HPLC システム名	HPLC-1
測定日	2018/12/03 12:20:13
注入量	10.00 [μL]
サンプル#	1
プロジェクト名	for you2
取込時間	90.0 [min]
測定シーケンス	hm0626 290 76 547
コントロールメソッド	Morita_1.0ml_210nm
ピーク ID テーブル	
検量線テーブル	
追加情報	

チャンネル情報＋ピーク情報

クロマトグラム名	hm547-CH1
サンプル名	
チャンネル名	CH1
データ取り込み間隔	500 [msec]
波形処理メソッド	（マニュアル）
数値計算式	
判定式	

#	ピーク名	CH	tR [min]	面積 [μV·sec]	高さ [μV]	面積%	高さ%	定量値	NTP	分離度	シンメトリー係数	警告
1	Unknown	1	9.175	1227247	112731	6.795	9.020	N/A	16433	6.808	1.264	
2	Unknown	1	11.450	16833150	1137073	93.205	90.980	N/A	14236	N/A	1.569	

(*S*)-2,3,3-trimethylnon-1-en-4-ol (8t).

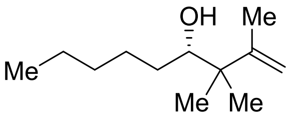

(*S*)-**8t**

Prepared according to the general procedure, then the crude material was purified by silica gel flash column chromatography (CH_2Cl_2/hexane = 1/10 to 1/0, v/v) to afford (*S*)-**8t** as colorless oil (43.6 mg, 95%). Enantiomeric excess was determined after protection of **8t** by *p*-NO_2-benzoyl chloride.

IR (neat) ν 3446, 2928, 2856, 1732, 1635, 1457, 1377, 1289, 1120, 1073, 934, 892 cm^{-1}; ^{1}H NMR ($CDCl_3$, 500 MHz) δ 0.89 (t, *J* = 6.9 Hz, 3H), 1.00 (s, 3H), 1.04 (s, 3H), 1.18-1.37 (m, 6H), 1.38-1.47 (m, 1H), 1.47-1.63 (m, 2H), 1.75 (d, *J* = 1.1 Hz, 3H), 3.46 (d, *J* = 10.3 Hz, 1H), 4.84 (s, 1H), 4.91 (s, 1H); ^{13}C NMR ($CDCl_3$, 125 MHz) δ 14.1, 19.7, 21.4, 22.6, 22.7, 27.1, 31.0, 32.0, 43.7, 75.5, 111.9, 151.1; LRMS (DART): *m*/*z* 633 [M+H$^+$]; HRMS (DART): *m*/*z* calculated for $C_{12}H_{25}O^+$ [M+H$^+$]: 185.1900, found: 185.1894; HPLC (chiral column: CHIRALPAK IF3; solvent: hexane/2-propanol = 200/1; flow rate: 1.0 mL/min; detection: at 254 nm): t_R = 7.3 min (minor) and 9.4 min (major); $[\alpha]_D^{20}$ = +70 (*c* = 0.26, $CHCl_3$, for 85% ee sample).

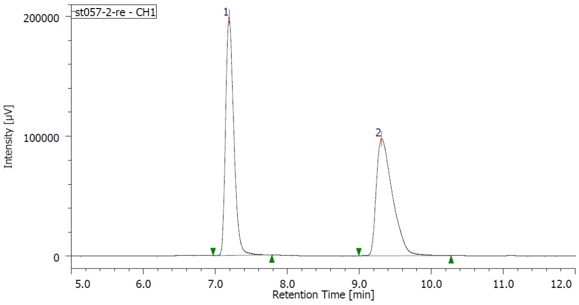

クロマトグラム情報
ユーザー名 a
更新日時 2018/06/13 19:30:34
コメント IF3 Hex IPA 200 1
HPLC システム名 HPLC-2
測定日 2018/06/13 19:02:08
注入量 10.00 [µL]
サンプル# 1
プロジェクト名 for you2
取込時間 90.0 [min]
測定シーケンス st057-IF3-hex-ipa-200-1-254nm-re
コントロールメソッド 1ml254nm
ピーク ID テーブル
検量線テーブル
追加情報

チャンネル情報＋ピーク情報
クロマトグラム名 st057-2-re-CH1
サンプル名
チャンネル名 CH1
データ取り込み間隔 500 [msec]
波形処理メソッド （マニュアル）
数値計算式
判定式

#	ピーク名	CH	tR [min]	面積 [µV·sec]	高さ [µV]	面積%	高さ%	定量値	NTP	分離度	シンメトリー係数	警告
1	Unknown	1	7.192	1571665	199023	50.730	67.104	N/A	19848	6.953	1.312	
2	Unknown	1	9.308	1526419	97568	49.270	32.896	N/A	8397	N/A	1.768	

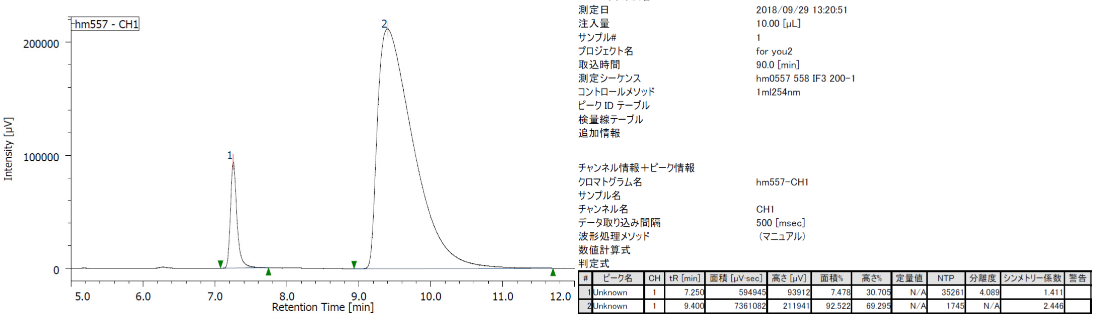

クロマトグラム情報
ユーザー名 a
更新日時 2018/09/30 1:01:43
コメント IF3 Hex IPA 200 1
HPLC システム名 HPLC-2
測定日 2018/09/29 13:20:51
注入量 10.00 [µL]
サンプル# 1
プロジェクト名 for you2
取込時間 90.0 [min]
測定シーケンス hm0557 558 IF3 200-1
コントロールメソッド 1ml254nm
ピーク ID テーブル
検量線テーブル
追加情報

チャンネル情報＋ピーク情報
クロマトグラム名 hm557-CH1
サンプル名
チャンネル名 CH1
データ取り込み間隔 500 [msec]
波形処理メソッド （マニュアル）
数値計算式
判定式

#	ピーク名	CH	tR [min]	面積 [µV·sec]	高さ [µV]	面積%	高さ%	定量値	NTP	分離度	シンメトリー係数	警告
1	Unknown	1	7.250	594945	93912	7.478	30.705	N/A	35261	4.089	1.411	
2	Unknown	1	9.400	7361082	211941	92.522	69.295	N/A	1745	N/A	2.446	

(*S*)-2,3,3,6-tetramethylhept-1-en-4-ol (8u).

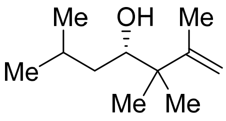

(*S*)-**8u**

Prepared according to the general procedure, then the crude material was purified by silica gel flash column chromatography (CH_2Cl_2/hexane = 1/10 to 1/0, v/v) to afford (*R*)-**8u** as colorless oil (33.3 mg, 78%). Enantiomeric excess was determined after protection of **8u** by *p*-NO_2-benzoylchloride.

IR (neat) ν 3408, 3091, 2957, 2970, 1635, 1468, 1377, 1296, 1069, 983, 893 cm^{-1}; ^1H NMR ($CDCl_3$, 500 MHz) δ 0.90 (t, J = 6.3 Hz, 3H), 0.94 (t, J = 6.9 Hz, 3H), 0.99 (s, 3H), 1.04 (s, 3H), 1.10-1.18 (m, 1H), 1.22-1.32 (m, 1H), 1.46 (d, J = 2.2 Hz, 1H), 1.73-1.85 (m, 4H), 3.56 (dd, J = 2.2, 10.3 Hz, 1H), 4.84 (s, 1H), 4.90 (s, 1H); ^{13}C NMR ($CDCl_3$, 125 MHz) δ 14.1, 19.7, 21.4, 22.6, 22.7, 27.1, 31.0, 32.0, 43.7, 75.5, 111.9, 151.1; LRMS (ESI): *m/z* 193 [$M+Na^+$]; HRMS (ESI): *m/z* calculated for $C_{11}H_{22}NaO^+$ [$M+Na^+$]: 193.1563, found: 193.1570; HPLC (chiral column: CHIRALPAK IF3; solvent: hexane/2-propanol = 200/1; flow rate: 1.0 mL/min; detection: at 254 nm): t_R = 7.2 min (minor) and 8.3 min (major); $[\alpha]_D^{20}$ = +116 (c = 0.84, $CHCl_3$, for 85% ee sample).

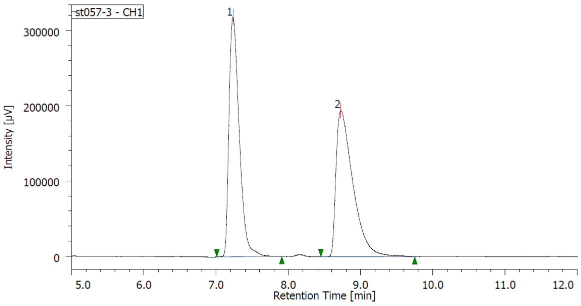

クロマトグラム情報
ユーザー名 a
更新日時 2018/06/13 19:30:33
コメント IF3 Hex IPA 200 1
HPLC システム名 HPLC-2
測定日 2018/06/13 17:42:05
注入量 10.00 [μL]
サンプル# 1
プロジェクト名 for you2
取込時間 90.0 [min]
測定シーケンス st057-IF3-hex-ipa-200-1-254nm
コントロールメソッド 1ml254nm
ピーク ID テーブル
検量線テーブル
追加情報

チャンネル情報＋ピーク情報
クロマトグラム名 st057-3-CH1
サンプル名
チャンネル名 CH1
データ取り込み間隔 500 [msec]
波形処理メソッド （マニュアル）
数値計算式
判定式

#	ピーク名	CH	tR [min]	面積 [μV·sec]	高さ [μV]	面積%	高さ%	定量値	NTP	分離度	シンメトリー係数	警告
1	Unknown	1	7.233	3153500	318811	49.986	62.133	N/A	12718	4.463	1.533	
2	Unknown	1	8.725	3155281	194301	50.014	37.867	N/A	7119	N/A	2.282	

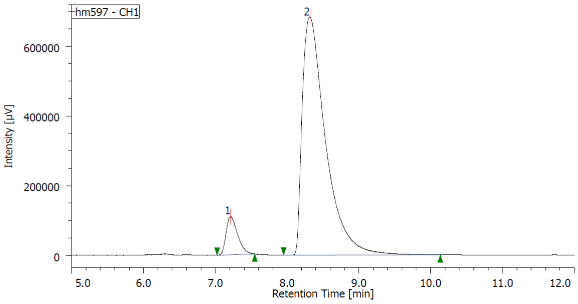

クロマトグラム情報
ユーザー名 a
更新日時 2018/10/16 13:16:48
コメント IF3 Hex IPA 200 1
HPLC システム名 HPLC-1
測定日 2018/10/16 13:03:11
注入量 10.00 [μL]
サンプル# 1
プロジェクト名 for you2
取込時間 90.0 [min]
測定シーケンス hm0597 IF3 200-1
コントロールメソッド 1ml254nm
ピーク ID テーブル
検量線テーブル
追加情報

チャンネル情報＋ピーク情報
クロマトグラム名 hm597-CH1
サンプル名
チャンネル名 CH1
データ取り込み間隔 500 [msec]
波形処理メソッド （マニュアル）
数値計算式
判定式

#	ピーク名	CH	tR [min]	面積 [μV·sec]	高さ [μV]	面積%	高さ%	定量値	NTP	分離度	シンメトリー係数	警告
1	Unknown	1	7.217	1219503	108816	7.342	13.737	N/A	9558	2.575	1.426	
2	Unknown	1	8.317	15390946	683322	92.658	86.263	N/A	3513	N/A	2.218	

<u>Determination of absolute configuration</u>

The absolute configurations of **8** were determined as follows:

8a-8k: The absolute configuration was determined by optical rotation value of **8b**. Those of others were assigned by analogy.

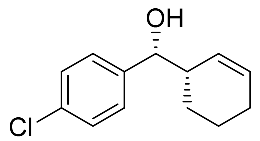

(*R*)-**8b**

This time: $[\alpha]_D^{20}$ = –24.6 (*c* = 1.0, C_6H_6, for 99% ee sample)

Reported value[13]: $[\alpha]_D^{20}$ = –15.0 (*c* = 1.0, C_6H_6, for 64% ee sample)

8l-8v: Following the following reaction scheme, diol **12** was obtained. The absolute configuration was determined by optical rotation value of diol **12**. Those of others were assigned by analogy.

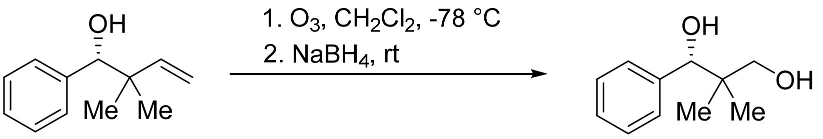

8v **12**

8v (38 mg, 0.216 mmol, 1 eq, 83% ee) were dissolved in CH_2Cl_2 (2 mL) in a round bottom flask. Then the flask was connected to an oxygen tank with a bubbler. The reaction mixture was cooled to -78 ºC. The ozone generator was switched on and the ozone went into the reaction flask. The reaction mixture was allowed to stir until the color changed to blue. $NaBH_4$ (16.8 mg, 0.431 mmol, 2 eq) and MeOH (1 ml) were added and the mixture was warmed up to room temperature. The reaction mixture was stirred for 15 h, and then quenched with saturated *aq.* NH_4Cl. The organic layer was separated, and the aqueous layer was further extracted three times with AcOEt. The combined organic layer was washed with water and brine and dried over Na_2SO_4. After the evaporation under reduced pressure, the residue was purified by silica gel flash column chromatography (hexane/AcOEt = 3/1 to 1/1, v/v) to give the desired diol **9** (30.2 mg, 78% yield) as a colorless oil. $[\alpha]_D^{20}$ = +65.9 (*c* = 2.5, $CHCl_3$, for 83% ee sample)

Reported optical rotation value[14]: $[\alpha]_D^{30}$ = +44.7 (*c* = 1.0, $CHCl_3$, for 99% ee sample)

4. Optimization Study for Aliphatic Aldehydes

During the optimization of racemic reaction, DCE solvent was found to be better solvent than DCM in terms of reactivity.

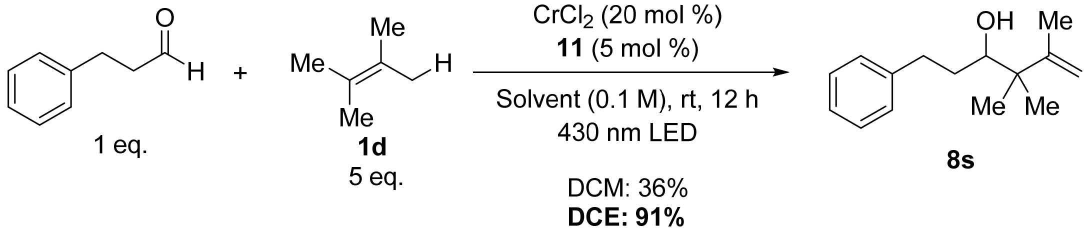

Addition of $MgPhPO_3$ was effective for improving enantioselectivity, while the reaction rate was slower than in the presence of $Mg(ClO_4)_2$. 48 hours was required for the completion of the reaction.

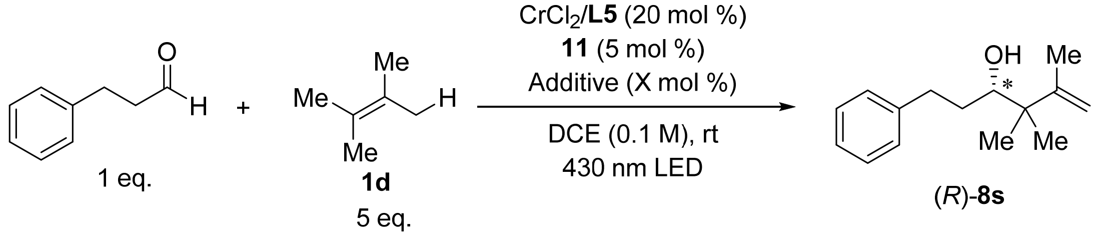

Mg(ClO$_4$)$_2$ (X = 100), 12 h: 67%, 48% ee
MgPhPO$_3$ (X = 20), 48 h: 82%, 81% ee

5. Mechanistic Study

5.1 Redox potentials of intermediates in catalytic cycle

We assumed that the proposed catalytic cycle (Figure 2 in the text) is feasible based on the following redox potentials of intermediates. Although oxidation potential of cyclohexene is higher than that of excited state of photocatalyst **10**, one electron transfer would be possible based on the related precedent (cyclopentene and photocatalyst **11**).[15] In addition, addition of Mg(ClO$_4$)$_2$ might facilitate this step by stabilizing ion pair **3** (see ref 20 in the text).

Cyclohexene **1a**: [**1a**/**3**] = +2.37 V versus SCE[16]

Photocatalyst **10**: [D–Acr$^{\bullet}$/ D$^{\bullet+}$–Acr$^{\bullet}$] = +2.24 V versus SCE, [D–Acr^{+}/ D–Acr$^{\bullet}$] = -0.46 V versus SCE[17]

Cr catalyst: [Cr(III)/Cr(II)] = -0.41 V versus SCE[18]

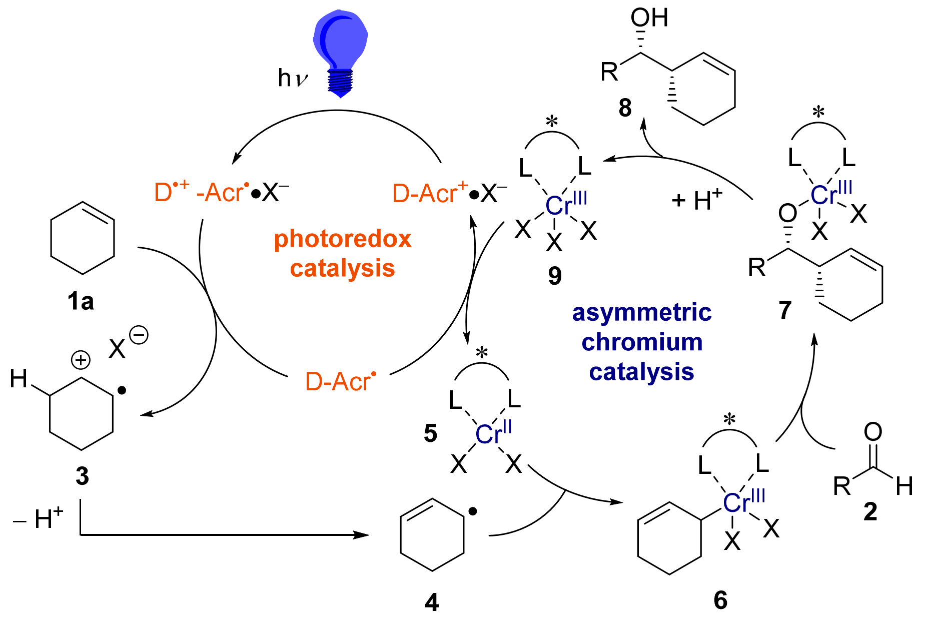

5.2 TEMPO trapping experiment

To confirm the generation of an allyl radical, a radical trapping experiment by the addition of TEMPO (2,2,6,6-tetramethyl-1-piperidinyloxy, free radical) was conducted.

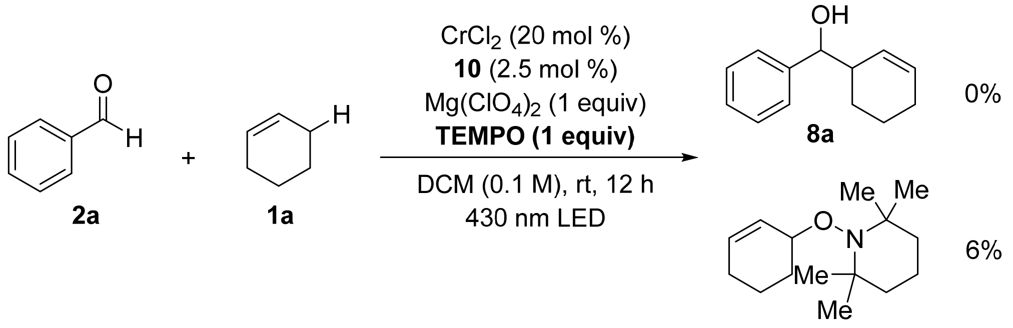

$CrCl_2$ (6.1 mg, 0.05 mmol, 20 mol %), cyclohexene **1a** (507 µL, 5.0 mmol, 20 eq), benzaldehyde **2a** (25.5 µL, 0.25 mmol, 1 eq), $Mg(ClO_4)_2$ (55.8 mg, 0.25 mmol, 100 mol %) and photocatalyst **10** (2.5 mg, 0.00625 mmol, 2.5 mol %) were dissolved in degassed CH_2Cl_2 (2.5 mL) in a screw-capped vial under argon atmosphere at room temperature. Then, TEMPO (39.1 mg, 0. 25 mmol, 1 eq) were added to the reaction mixture. The vial was subjected to 430 nm LED irradiation for 12 hours under temperature control (ca. 27–29 ºC). Then, the reaction mixture was passed through a pad of silica gel with CH_2Cl_2. After evaporation, the crude mixture was analyzed by NMR spectroscopy using 1,1,2,2-tetrachloroethane as internal standard. No desired product **8a** was formed, and TEMPO adduct[19] was detected in 6% NMR yield. Therefore, we assume allyl radical is formed as an intermediate of the reaction.

5.3 Radical clock experiment

To deny the involvement of ketyl radicals, a radical clock experiment was performed using 2-phenylcyclopropylcarbaldehyde as electrophile.

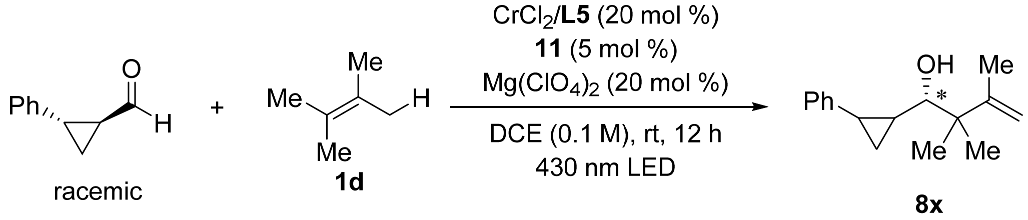

$CrCl_2$ (6.1 mg, 0.05 mmol, 20 mol %) and **L5** (17.9 mg, 0.05 mmol, 20 mol %) were dissolved in degassed 1,2-dichloroethane (2.5 mL) in a screw-capped vial under argon atmosphere and stirred for 1 hour at room temperature. 2,3-Dimethyl-2-butene **1d** (119 µL, 1.0 mmol, 5 eq), $Mg(ClO_4)_2$ (11.2 mg, 0.05 mmol, 20 mol %), 2-phenylcyclopropylcarbaldehyde[20] (33 µL, 0.25 mmol, 1 eq) and photocatalyst **11** (5.2 mg, 0.0125 mmol, 5 mol %) were added to the reaction mixture. The vial was subjected to 430 nm LED irradiation for 12 hours under temperature control (ca. 27–29 ºC). Then, the reaction mixture was passed through a pad of silica gel with a CH_2Cl_2 eluent. After evaporation, the crude mixture was analyzed by NMR spectroscopy using 1,1,2,2-tetrachloroethane as internal standard. Desired product **8x** was formed (77%, 1.1/1 dr), and no ring opened product was observed. Therefore, we assume that ketyl radicals are not formed in the reaction.

5.4 Alternative pathway of chirality induction

We studied a feasibility of an alternative asymmetric induction mechanism involving in situ-generated $Mg(ClO_4)_2$/**L5** as chiral Lewis acid by premixing $Mg(ClO_4)_2$ and **L5**. However, the desired reaction did not proceed at all, indicating that **L5** remains coordinating to the chromium atom.

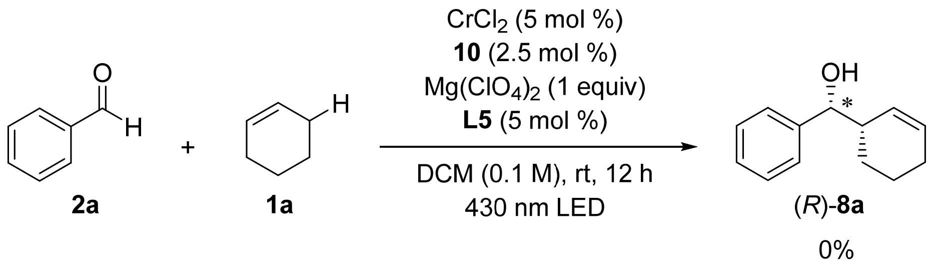

$Mg(ClO_4)_2$ (55.8 mg, 0.25 mmol, 100 mol %) and **L5** (4.5 mg, 0.0125 mmol, 5 mol %) were dissolved in degassed CH_2Cl_2 (2.5 mL) in a screw-capped vial under argon atmosphere and the mixture was stirred for 1 hour at room temperature. Cyclohexene **1a** (507 μL, 5.0 mmol, 20 eq), $CrCl_2$ (1.5 mg, 0.0125 mmol, 5 mol %), benzaldehyde **2a** (25.5 μL, 0.25 mmol, 1 eq) and photocatalyst **10** (2.5 mg, 0.00625 mmol, 2.5 mol %) were added to the reaction mixture. The vial was subjected to 430 nm LED irradiation for 12 hours under temperature control (ca. 27–29 ºC). Then, the reaction mixture was passed through a pad of silica gel with CH_2Cl_2. After evaporation, the crude mixture was analyzed by NMR. However, no desired product **8a** was observed. Therefore, we assume that the asymmetric induction is not due to $Mg(ClO_4)_2$/**L5**, but due to $CrCl_2$/**L5**.

5.5 Transient absorption analysis

Nanosecond time-resolved transient absorption measurements were carried out using the laser system provided by UNISOKU Co., Ltd. Measurements of nanosecond transient absorption spectrum were performed according to the following procedure. A deaerated solution containing a sample in a quartz cell (1 cm × 1 cm) was excited by a Nd:YAG laser (Continuum SLII-10, 4-6 ns fwhm, λex = 355 nm, 5mJ/pulese). The photodynamics were monitored by continuous exposure to a xenon lamp (150 W) as a probe light and a photomultiplier tube (Hamamatsu 2949) as a detector. The solution in a quartz cuvette was deoxygenated by nitrogen purging for 10 min prior to measurements. In following experiments, the sample containing $Mg(ClO_4)_2$ was prepared using saturated $Mg(ClO_4)_2$ solution in DCM. ($Mg(ClO_4)_2$ (2 mg) was added to DCM (10 ml) and sonicated 30 min at room temperature. The supernatant was used as saturated $Mg(ClO_4)_2$ solution in DCM.)

6. References

1) C. Chen, K. Tagami, Y. Kishi, *J. Org. Chem.* **1995**, *60*, 5386.

2) W. Chen, Q. Yang, T. Zhou, Q. Tian, G. Zhang, *Org. Lett.* **2015**, *17*, 5236.

3) M. Kurosu, M. -H. Lin, Y. Kishi, *J. Am. Chem. Soc.* **2004**, *126*, 12248.

4) J. L. Hofstra, A. H. Cherney, C. M. Ordner, S. E. Reisman, *J. Am. Chem. Soc.* **2018**, *140*, 139.

5) M. Hatano, K. Moriyama, T. Maki, K. Ishihara, *Angew. Chem. Int. Ed.* **2010**, *49*, 3823.

6) A. G. Griesbeck, S. Stadtmiiller, *J. Am. Chem. Soc.* **1991**, *113*, 6923.

7) G. P. Howell, A. J. Minnaard, B. L. Feringa, *Org. Biomol. Chem.* **2006**, *4*, 1278.

8) W. Wang, T. Zhang, M. Shi, *Organometallics* **2009**, *28*, 2640.

9) H. C. Brown, K. S. Bhat, P. K. Jadhav, *J. Chem. Soc., Perkin Trans. 1,* **1991**, *0*, 2633.

10) M. Kimura, D. Nojiri, M. Fukushima, S. Oi, Y. Sonoda, Y. Inoue, *Org. Lett.* **2009**, *11*, 3794.

11) a) E. Skucas, J. F. Bower, M. J. Krische, *J. Am. Chem. Soc.* **2007**, *129*, 12678. b) F. Amemiya, K. Fuse, T. Fuchigamia, M. Atobe, *Chem. Commun.* **2010**, *46*, 2730.

12) N. Hayashi, H. Honda, M. Yasuda, I. Shibata, A. Baba, *Org. Lett.* **2006**, *8*, 4553.

13) W. Wang, T. Zhang, M. Shi, *Organometallics* **2009**, *28*, 2640.

14) T. Ichibakase, M. Nakajima, *Org. Lett.* **2011**, *13*, 1579.

15) R. Zhou, H. Liu, H. Tao, X. Yu, J. Wu, *Chem. Sci.* **2017**, *8*, 4654.

16) H. G. Roth, N. A. Romero, D. A. Nicewicz, *Synlett* **2016**, *27*, 714.

17) T. Tsudaka, H. Kotani, K. Ohkubo, T. Nakagawa, N. V. Tkachenko, H. Lemmetyinen, S. Fukuzumi, *Chem. Eur. J.* **2017**, *23*, 1306.

18) A. Fürstner, N. Shi, *J. Am. Chem. Soc.* **1996**, *118*, 12349.

19) L. Li, Z. Yu, Z. Shen, *Adv. Synth. Catal.* **2015**, *357*, 3495.

20) A. Weber, K. Doehl, J. Sachs, A. C. M. Nordschild, D. Schroeder, A. Kulik, T. Fischer, L. Schmitt, N. Teusch, J. Pietruszka, *Biomol. Med. Chem.* **2017**, *25*, 6115.

7. NMR Charts

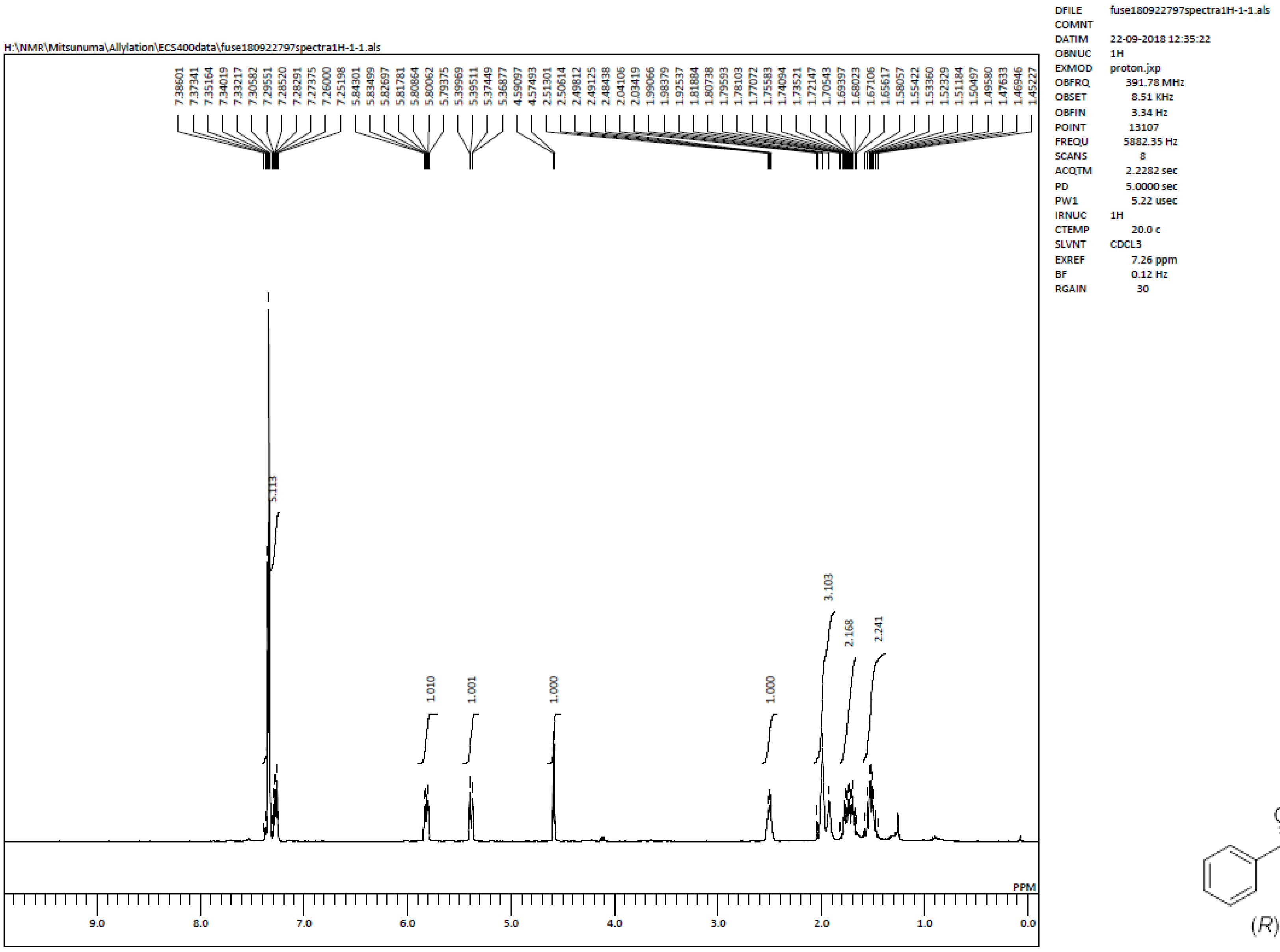

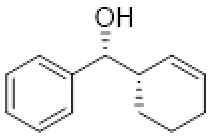

(*R*)-**8a**

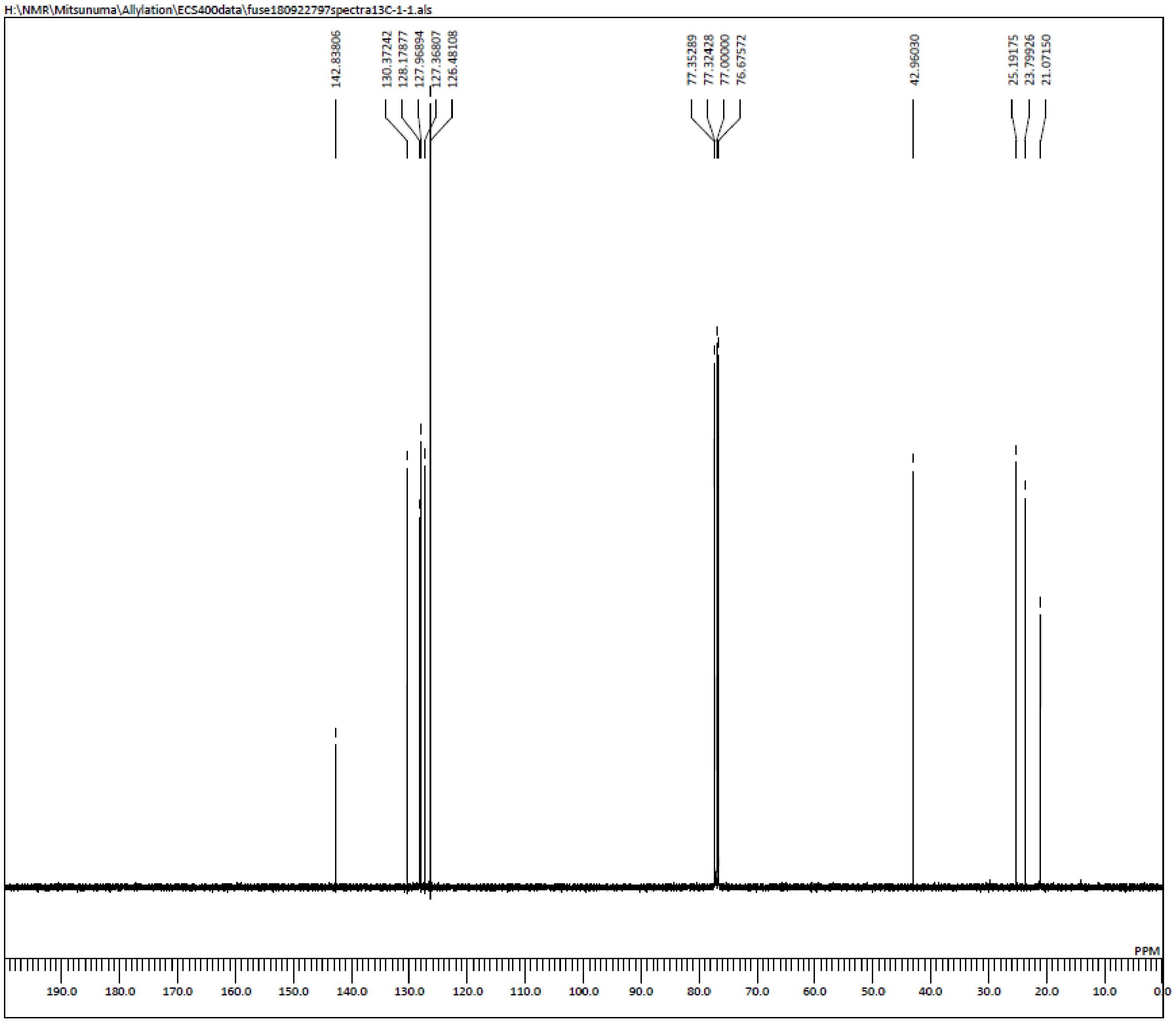

DFILE	fuse180922797spectra13C-1-1.als
COMNT	
DATIM	22-09-2018 12:37:14
OBNUC	13C
EXMOD	carbon.jxp
OBFRQ	98.52 MHz
OBSET	4.64 KHz
OBFIN	8.74 Hz
POINT	26214
FREQU	24630.54 Hz
SCANS	1693
ACQTM	1.0643 sec
PD	2.0000 sec
PW1	3.12 usec
IRNUC	1H
CTEMP	20.3 c
SLVNT	CDCL3
EXREF	77.00 ppm
BF	0.12 Hz
RGAIN	60

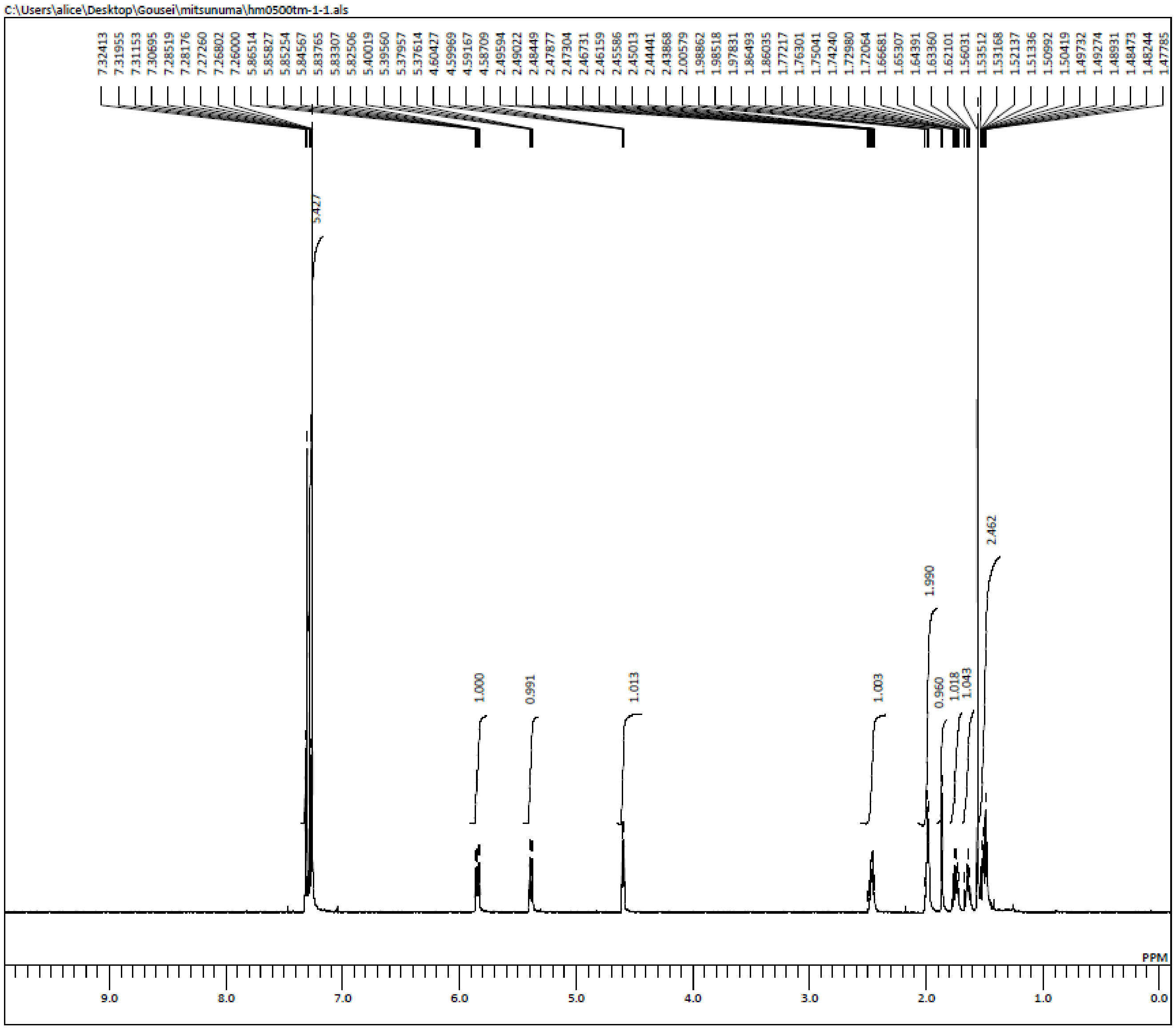

DFILE	hm0500tm-1-1.als
COMNT	
DATIM	2018-09-17 17:08:10
OBNUC	1H
EXMOD	proton.jxp
OBFRQ	500.16 MHz
OBSET	2.41 KHz
OBFIN	6.01 Hz
POINT	13107
FREQU	7507.51 Hz
SCANS	8
ACQTM	1.7459 sec
PD	5.0000 sec
PW1	5.55 usec
IRNUC	1H
CTEMP	21.5 c
SLVNT	CDCL3
EXREF	7.26 ppm
BF	0.12 Hz
RGAIN	34

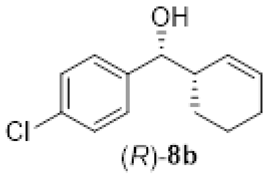

(*R*)-**8b**

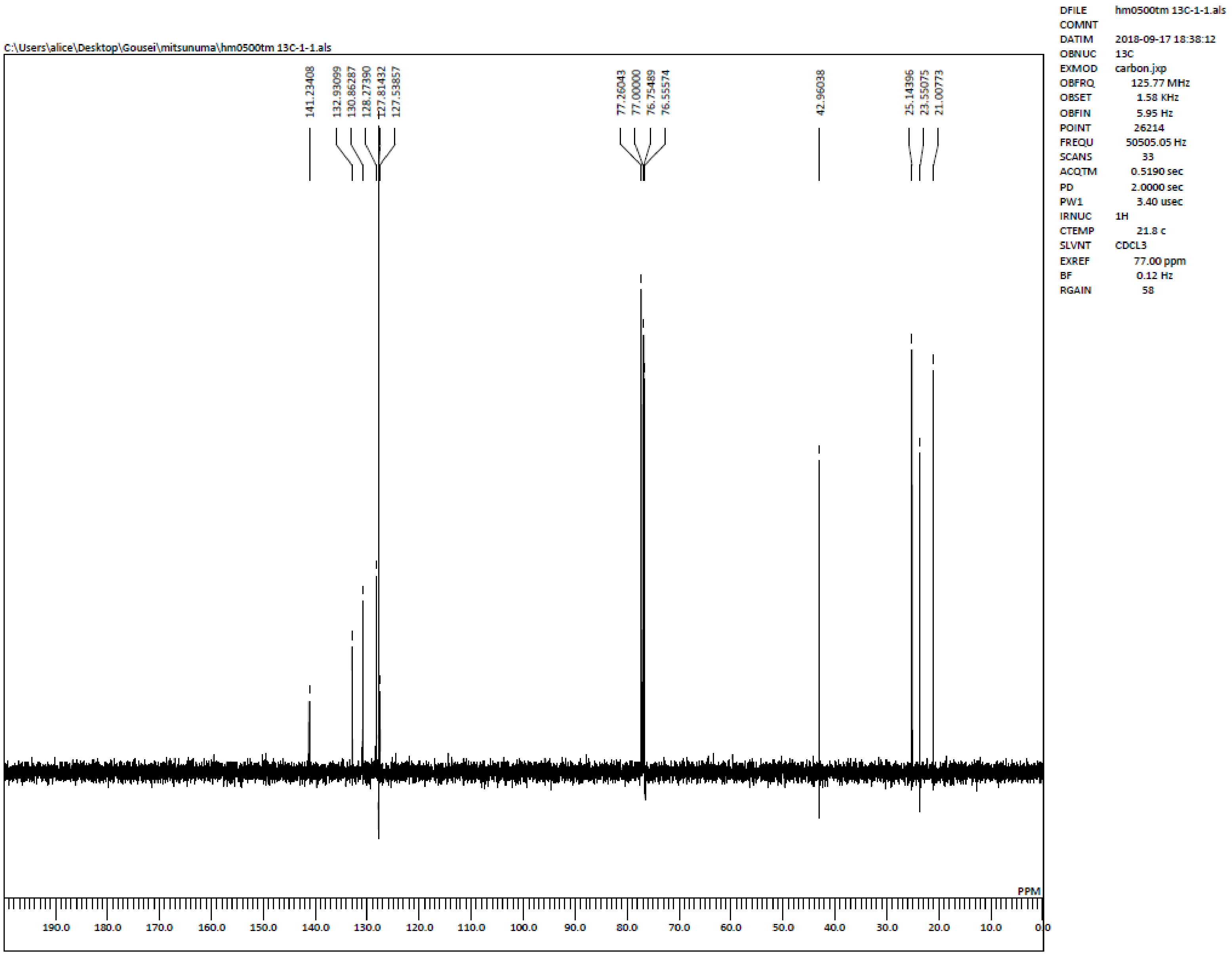

C:\Users\alice\Desktop\Gousei\mitsunuma\hm0500tm 13C-1-1.als
141.23408
132.93099
130.86287
128.27390
127.81432
127.53857
77.26043
77.00000
76.75489
76.55574
42.96038
25.14396
23.55075
21.00773
PPM
190.0 180.0 170.0 160.0 150.0 140.0 130.0 120.0 110.0 100.0 90.0 80.0 70.0 60.0 50.0 40.0 30.0 20.0 10.0 0.0
DFILE hm0500tm 13C-1-1.als
COMNT
DATIM 2018-09-17 18:38:12
OBNUC 13C
EXMOD carbon.jxp
OBFRQ 125.77 MHz
OBSET 1.58 KHz
OBFIN 5.95 Hz
POINT 26214
FREQU 50505.05 Hz
SCANS 33
ACQTM 0.5190 sec
PD 2.0000 sec
PW1 3.40 usec
IRNUC 1H
CTEMP 21.8 c
SLVNT CDCL3
EXREF 77.00 ppm
BF 0.12 Hz
RGAIN 58

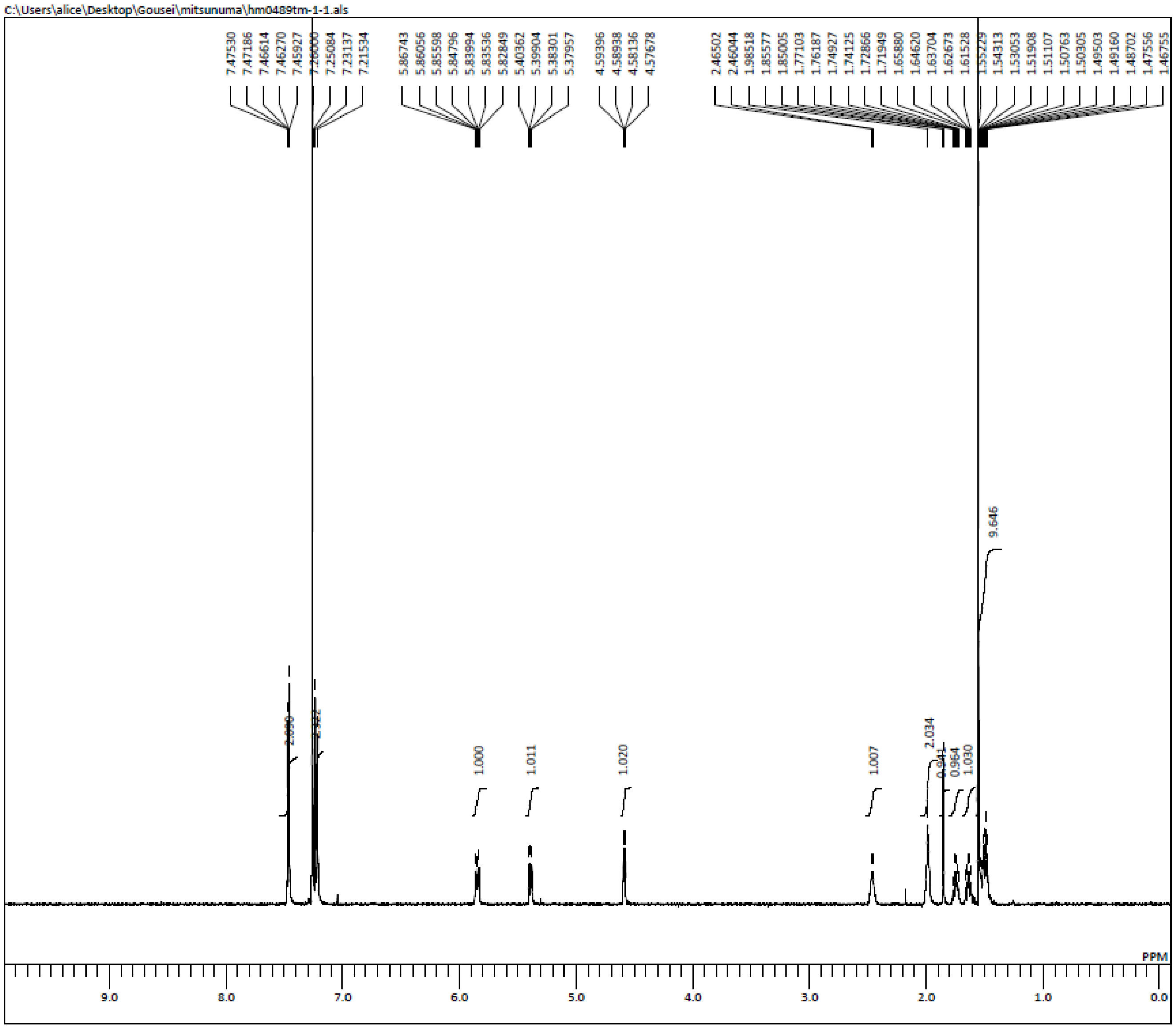

DFILE hm0489tm-1-1.als
COMNT
DATIM 2018-09-16 23:30:58
OBNUC 1H
EXMOD proton.jxp
OBFRQ 500.16 MHz
OBSET 2.41 KHz
OBFIN 6.01 Hz
POINT 13107
FREQU 7507.51 Hz
SCANS 8
ACQTM 1.7459 sec
PD 5.0000 sec
PW1 5.55 usec
IRNUC 1H
CTEMP 21.7 c
SLVNT CDCL3
EXREF 7.26 ppm
BF 0.12 Hz
RGAIN 34

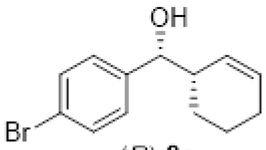

(*R*)-**8c**

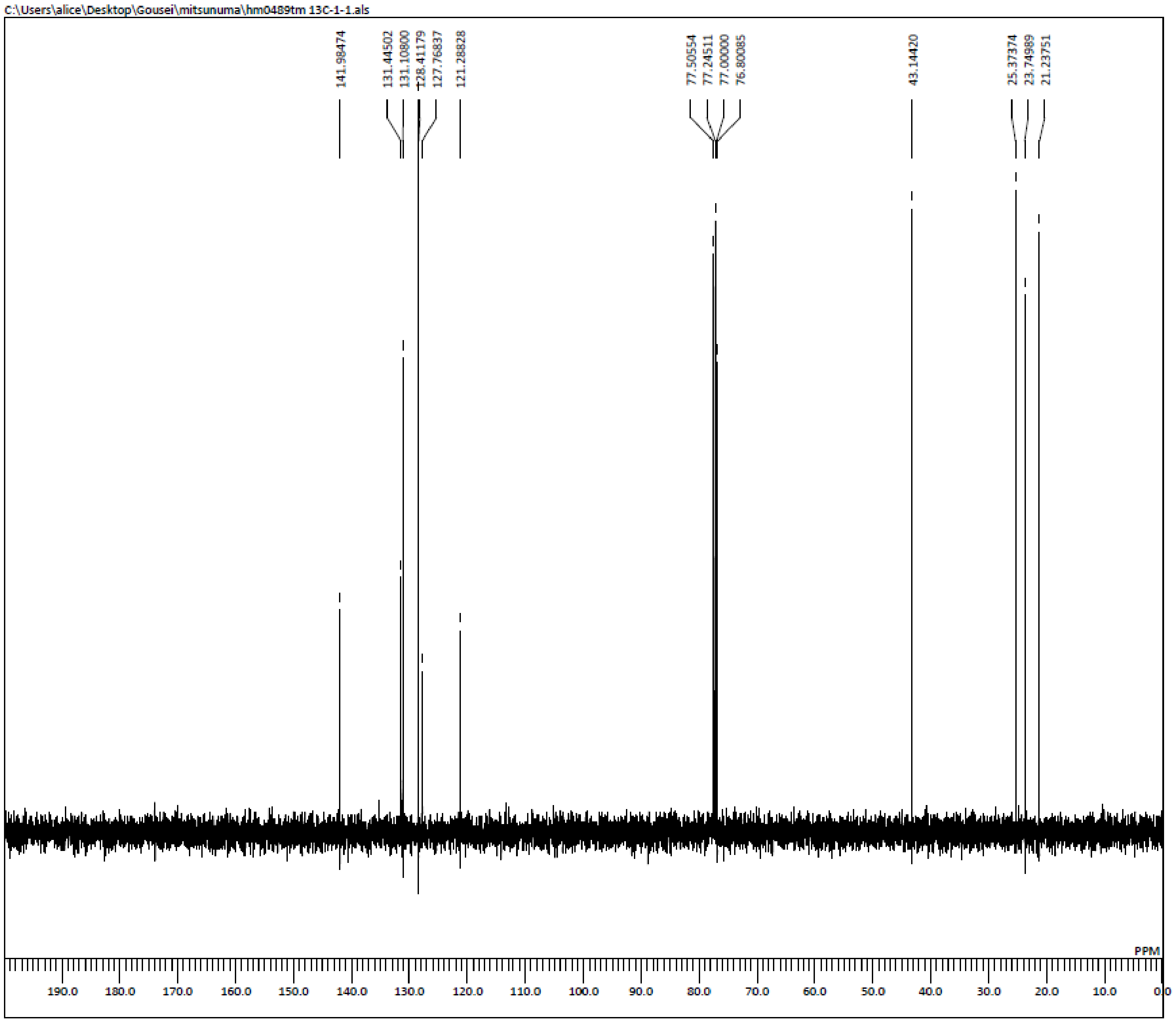

DFILE	hm0489tm 13C-1-1.als
COMNT	
DATIM	2018-09-17 17:15:15
OBNUC	13C
EXMOD	carbon.jxp
OBFRQ	125.77 MHz
OBSET	1.58 KHz
OBFIN	5.95 Hz
POINT	26214
FREQU	50505.05 Hz
SCANS	20
ACQTM	0.5190 sec
PD	2.0000 sec
PW1	3.40 usec
IRNUC	1H
CTEMP	21.8 c
SLVNT	CDCL3
EXREF	77.00 ppm
BF	0.12 Hz
RGAIN	58

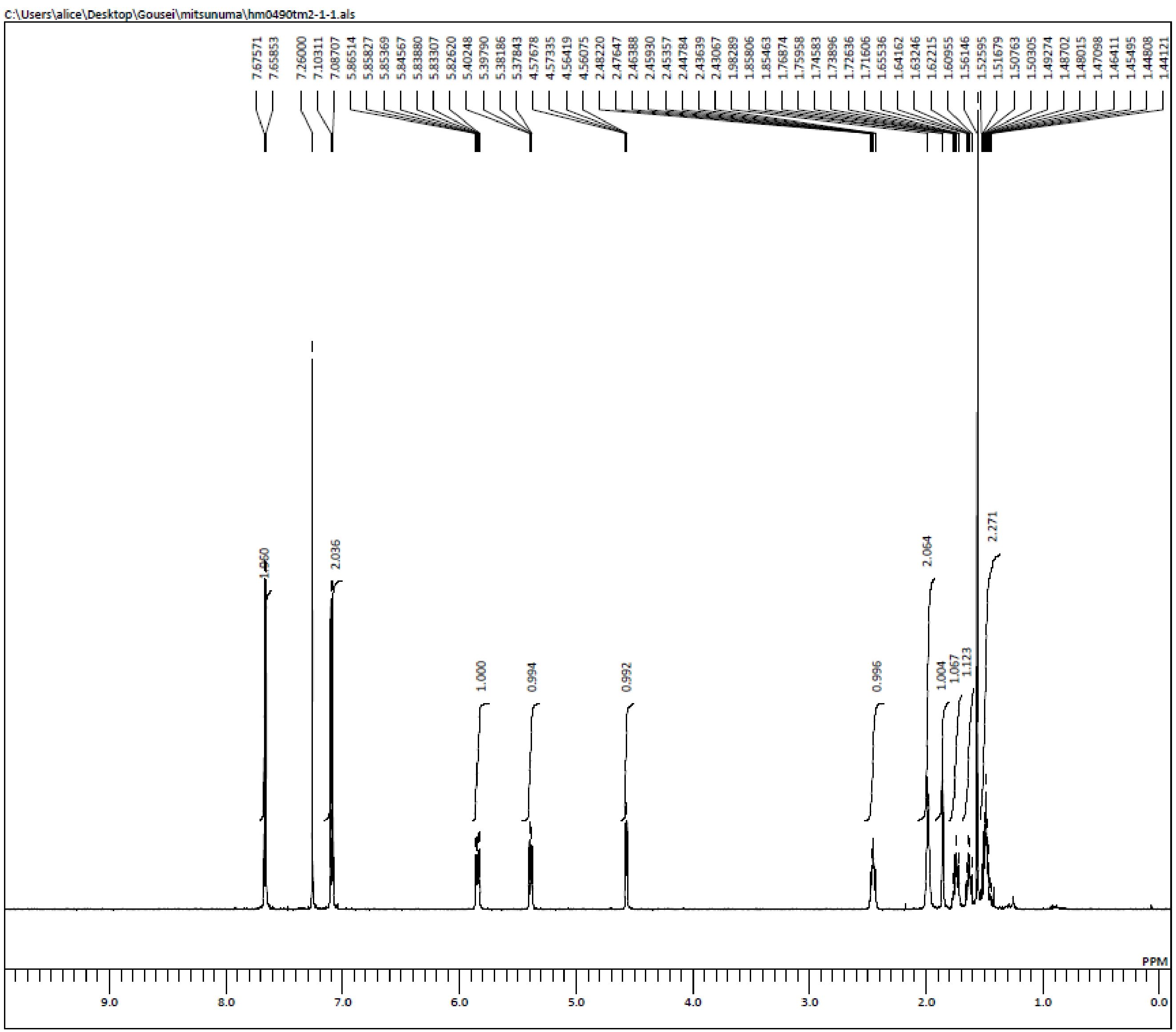

DFILE hm0490tm2-1-1.als
COMNT
DATIM 2018-09-17 18:31:03
OBNUC 1H
EXMOD proton.jxp
OBFRQ 500.16 MHz
OBSET 2.41 KHz
OBFIN 6.01 Hz
POINT 13107
FREQU 7507.51 Hz
SCANS 8
ACQTM 1.7459 sec
PD 5.0000 sec
PW1 5.55 usec
IRNUC 1H
CTEMP 21.9 c
SLVNT CDCL3
EXREF 7.26 ppm
BF 0.12 Hz
RGAIN 34

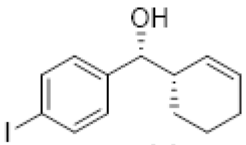

(*R*)-**8d**

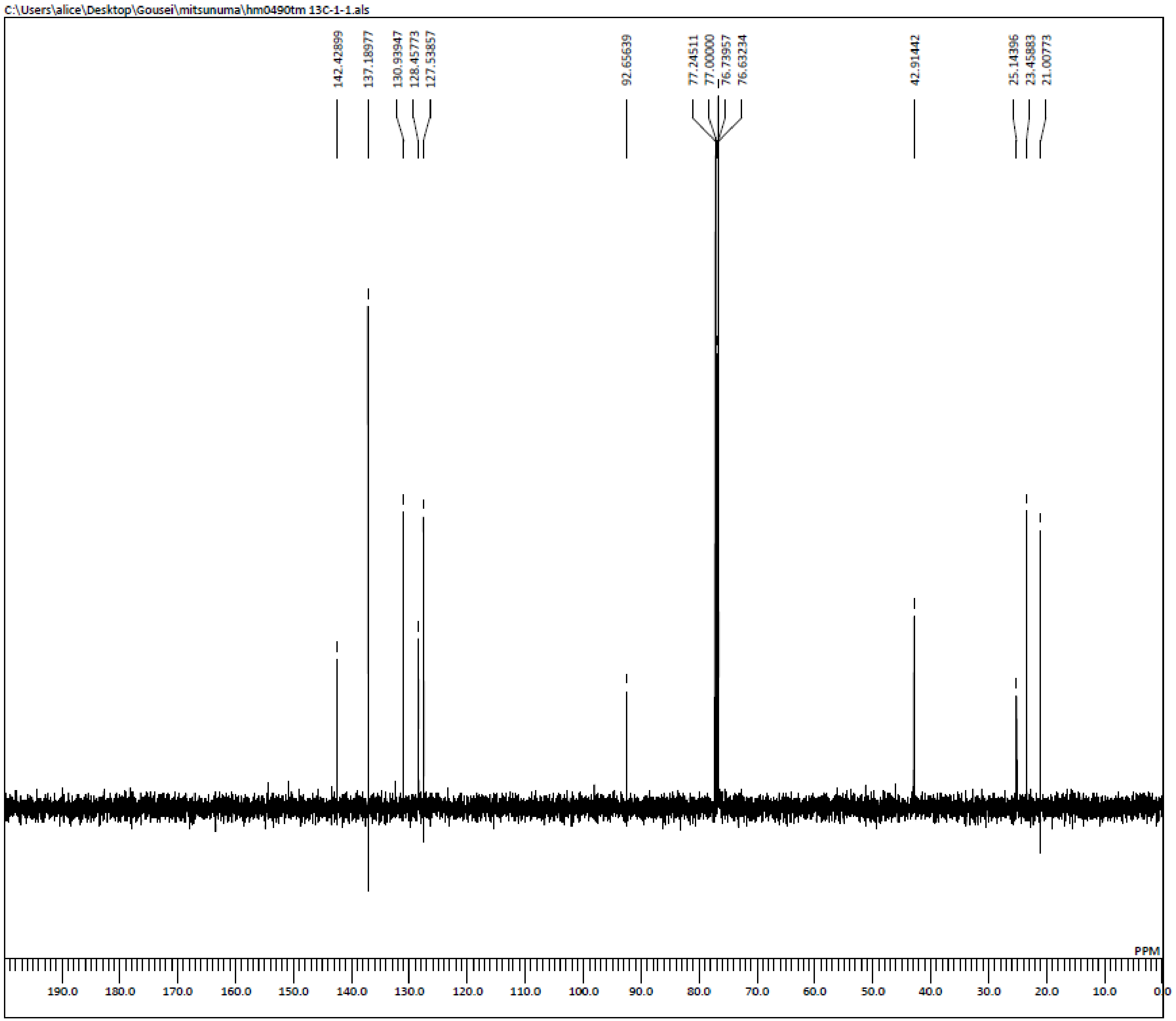

DFILE	hm0490tm 13C-1-1.als
COMNT	
DATIM	2018-09-17 19:02:45
OBNUC	13C
EXMOD	carbon.jxp
OBFRQ	125.77 MHz
OBSET	1.58 KHz
OBFIN	5.95 Hz
POINT	26214
FREQU	50505.05 Hz
SCANS	60
ACQTM	0.5190 sec
PD	2.0000 sec
PW1	3.40 usec
IRNUC	1H
CTEMP	21.8 c
SLVNT	CDCL3
EXREF	77.00 ppm
BF	0.12 Hz
RGAIN	58

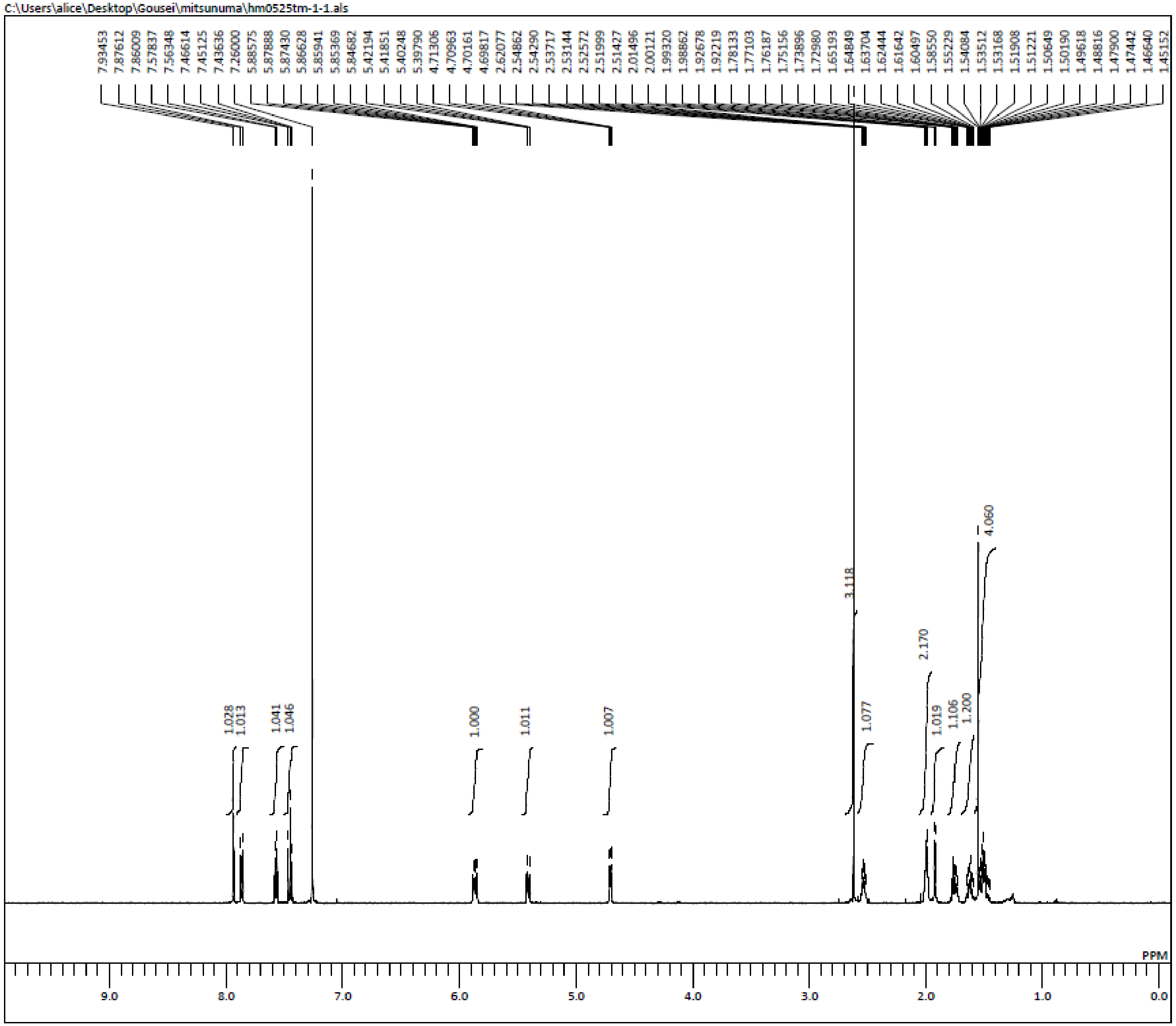

DFILE hm0525tm-1-1.als
COMNT
DATIM 2018-09-20 19:57:29
OBNUC 1H
EXMOD proton.jxp
OBFRQ 500.16 MHz
OBSET 2.41 KHz
OBFIN 6.01 Hz
POINT 13107
FREQU 7507.51 Hz
SCANS 8
ACQTM 1.7459 sec
PD 5.0000 sec
PW1 5.55 usec
IRNUC 1H
CTEMP 21.4 c
SLVNT CDCL3
EXREF 7.26 ppm
BF 0.12 Hz
RGAIN 34

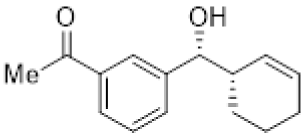

(*R*)-**8e**

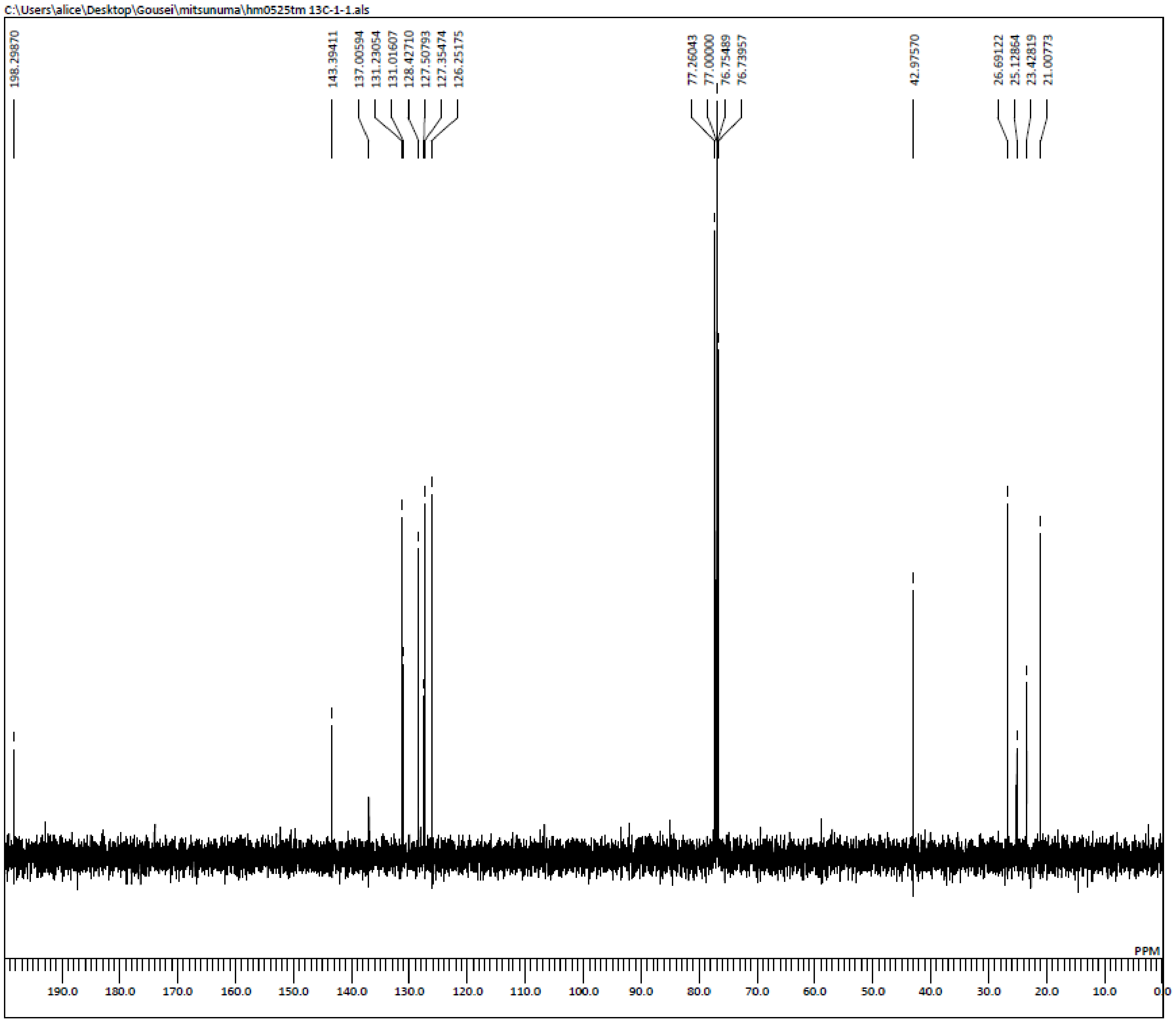

DFILE	hm0525tm 13C-1-1.als
COMNT	
DATIM	2018-09-20 20:17:42
OBNUC	13C
EXMOD	carbon.jxp
OBFRQ	125.77 MHz
OBSET	1.58 KHz
OBFIN	5.95 Hz
POINT	26214
FREQU	50505.05 Hz
SCANS	28
ACQTM	0.5190 sec
PD	2.0000 sec
PW1	3.40 usec
IRNUC	1H
CTEMP	21.5 c
SLVNT	CDCL3
EXREF	77.00 ppm
BF	0.12 Hz
RGAIN	60

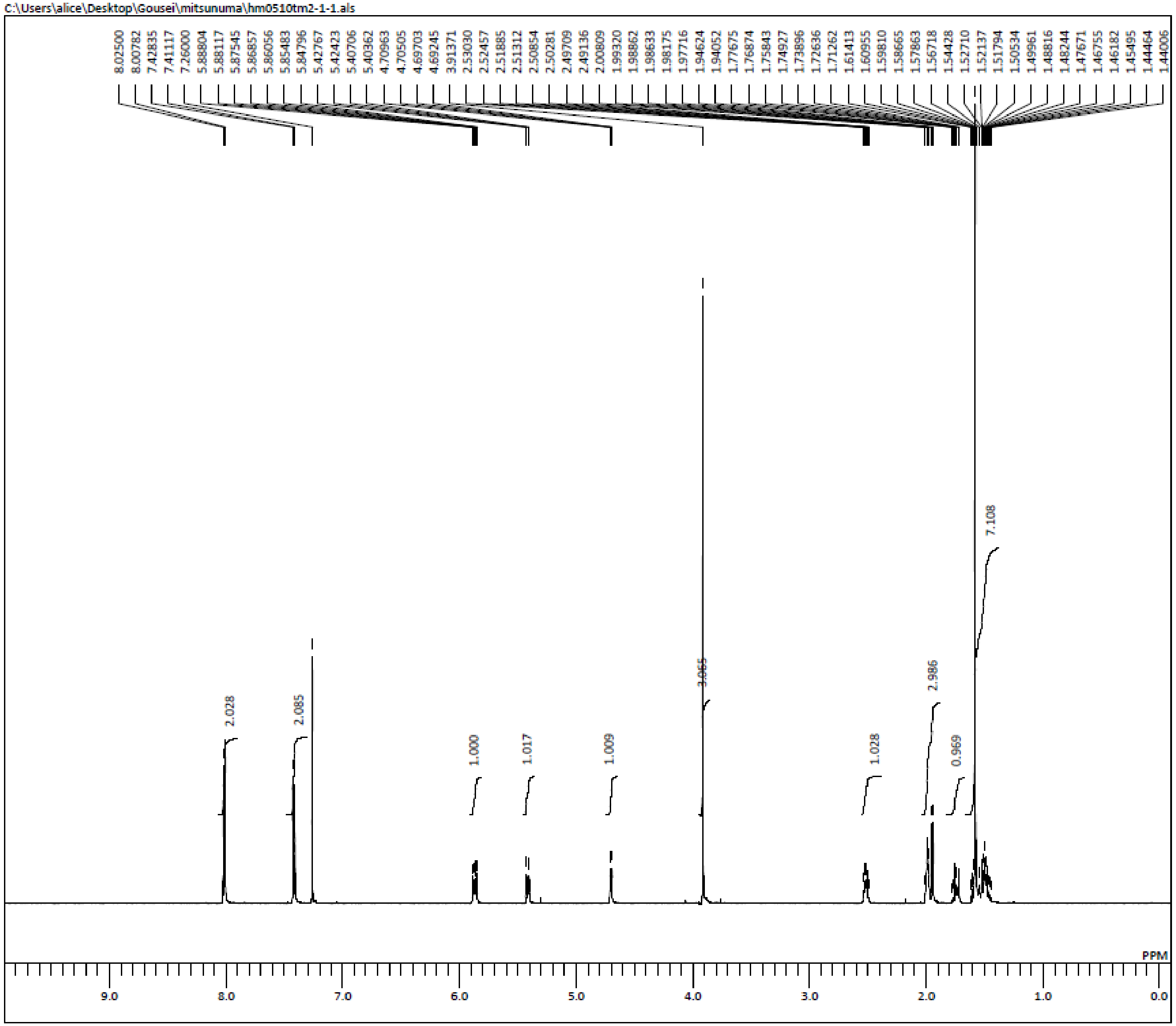

DFILE	hm0510tm2-1-1.als
COMNT	
DATIM	2018-09-19 01:32:34
OBNUC	1H
EXMOD	proton.jxp
OBFRQ	500.16 MHz
OBSET	2.41 KHz
OBFIN	6.01 Hz
POINT	13107
FREQU	7507.51 Hz
SCANS	8
ACQTM	1.7459 sec
PD	5.0000 sec
PW1	5.55 usec
IRNUC	1H
CTEMP	21.9 c
SLVNT	CDCL3
EXREF	7.26 ppm
BF	0.12 Hz
RGAIN	32

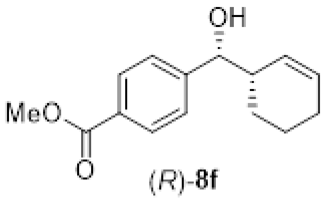

(*R*)-**8f**

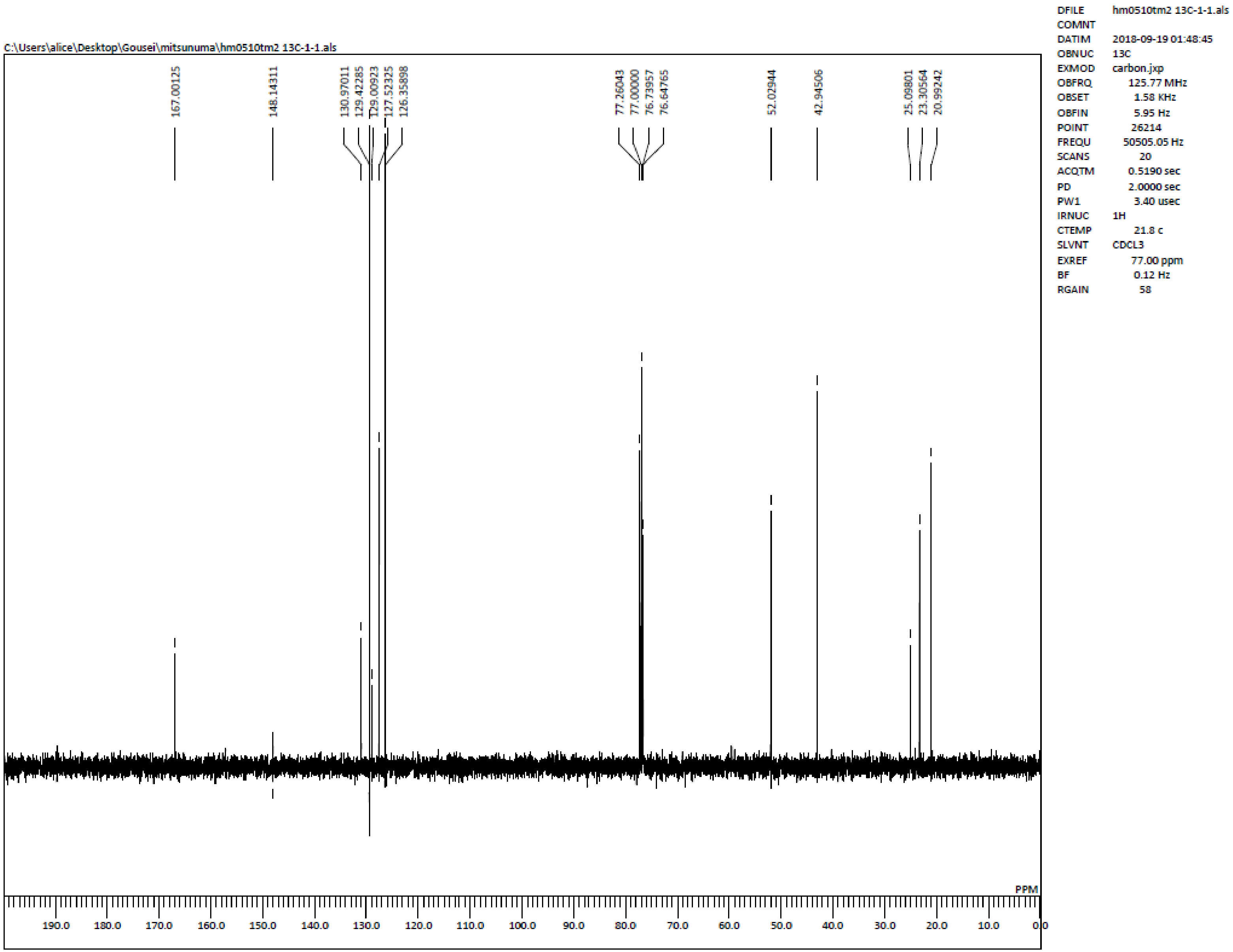
C:\Users\alice\Desktop\Gousei\mitsunuma\hm0510tm2 13C-1-1.als
167.00125
148.14311
130.97011
129.42285
129.00923
127.52325
126.35898
77.26043
77.00000
76.73957
76.64765
52.02944
42.94506
25.09801
23.30564
20.99242
PPM
190.0
180.0
170.0
160.0
150.0
140.0
130.0
120.0
110.0
100.0
90.0
80.0
70.0
60.0
50.0
40.0
30.0
20.0
10.0
0.0
DFILE hm0510tm2 13C-1-1.als
COMNT
DATIM 2018-09-19 01:48:45
OBNUC 13C
EXMOD carbon.jxp
OBFRQ 125.77 MHz
OBSET 1.58 KHz
OBFIN 5.95 Hz
POINT 26214
FREQU 50505.05 Hz
SCANS 20
ACQTM 0.5190 sec
PD 2.0000 sec
PW1 3.40 usec
IRNUC 1H
CTEMP 21.8 c
SLVNT CDCL3
EXREF 77.00 ppm
BF 0.12 Hz
RGAIN 58

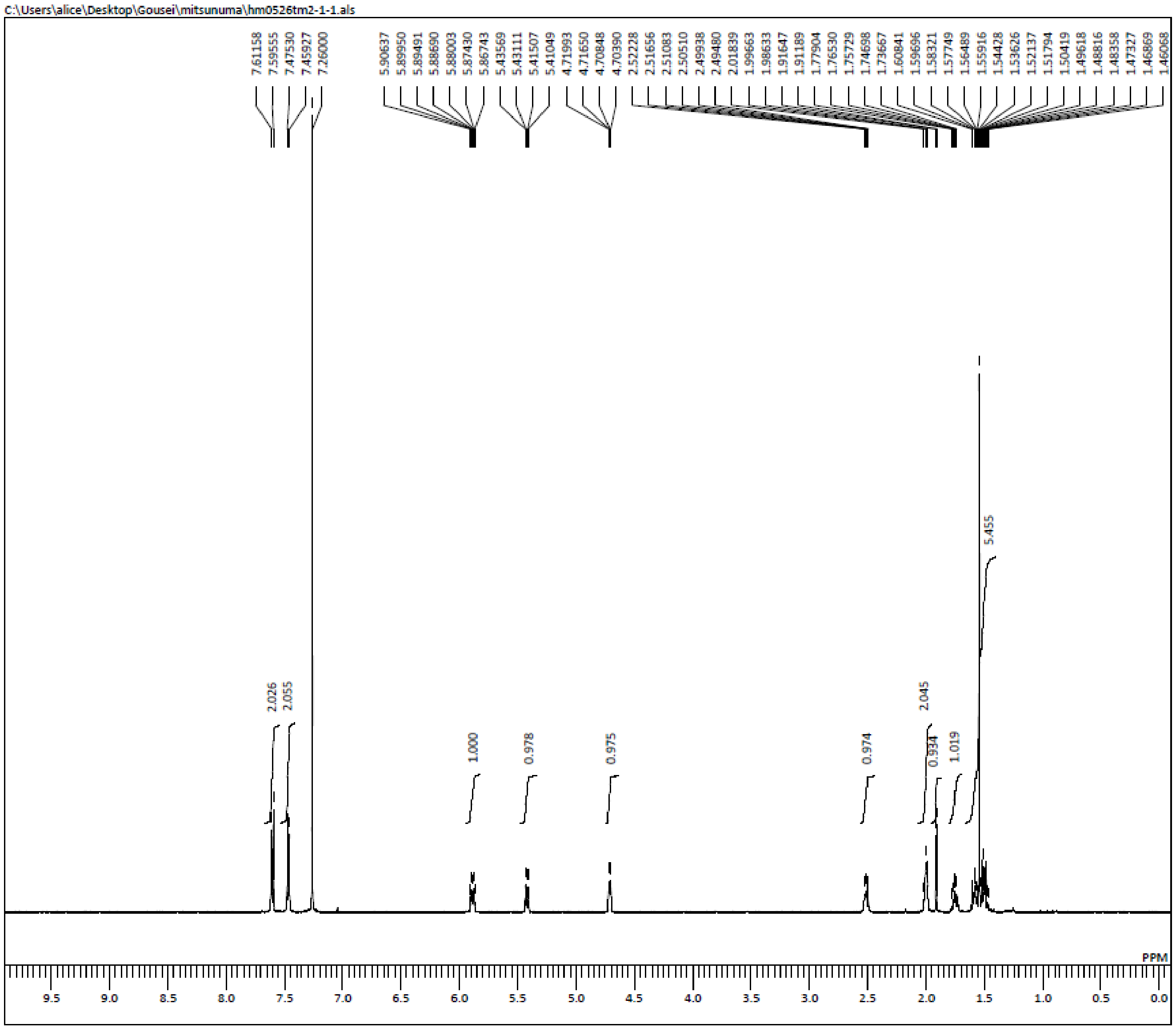

DFILE hm0526tm2-1-1.als
COMNT
DATIM 2018-09-20 20:01:07
OBNUC 1H
EXMOD proton.jxp
OBFRQ 500.16 MHz
OBSET 2.41 KHz
OBFIN 6.01 Hz
POINT 13107
FREQU 7507.51 Hz
SCANS 8
ACQTM 1.7459 sec
PD 5.0000 sec
PW1 5.55 usec
IRNUC 1H
CTEMP 21.4 c
SLVNT CDCL3
EXREF 7.26 ppm
BF 0.12 Hz
RGAIN 34

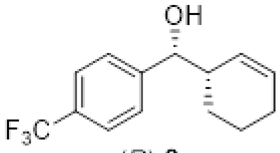

(*R*)-**8g**

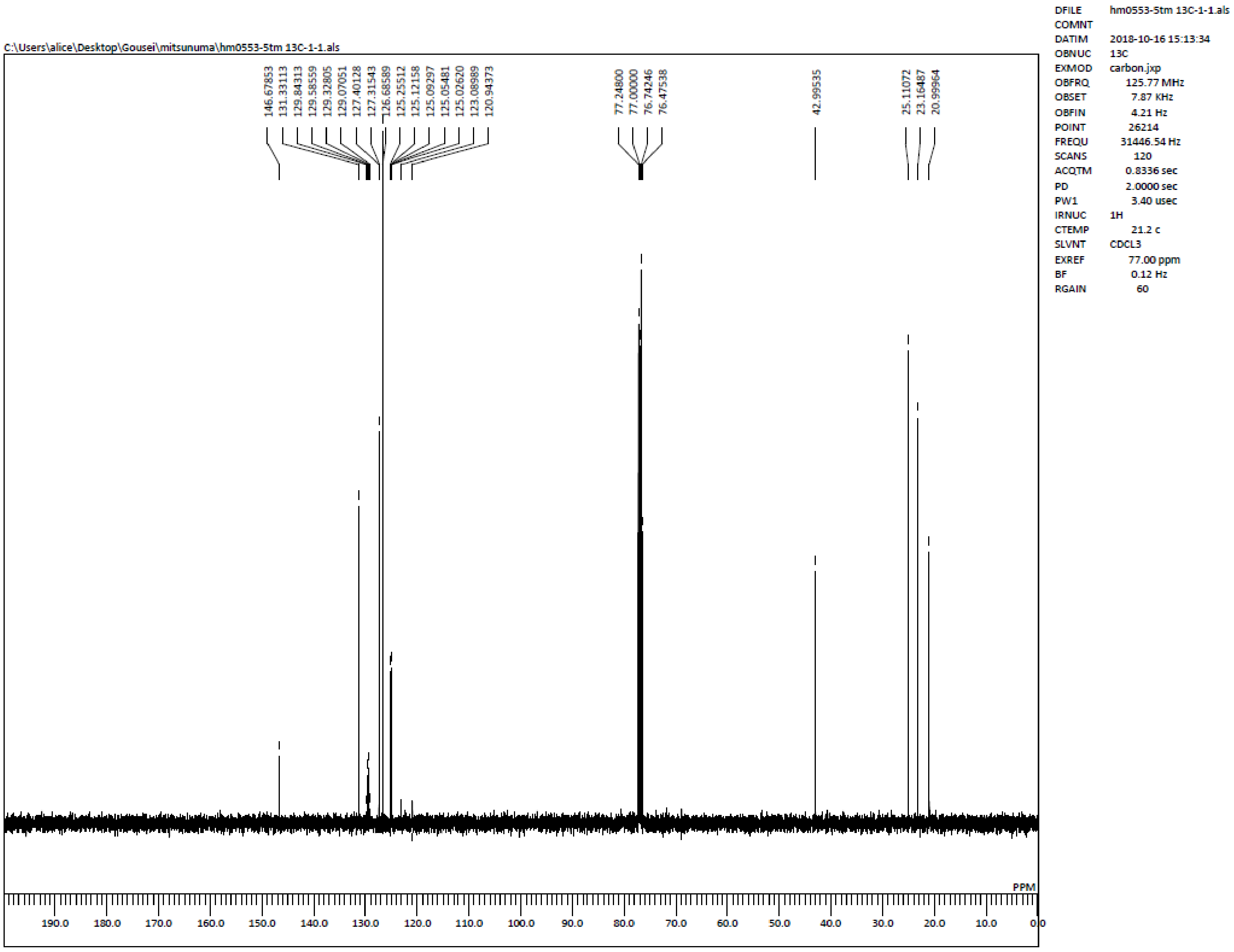
C:\Users\alice\Desktop\Gousei\mitsunuma\hm0553-5tm 13C-1-1.als
146.67853
131.33113
129.84313
129.58559
129.32805
129.07051
127.40128
127.31543
126.68589
125.25512
125.12158
125.09297
125.05481
125.02620
123.08989
120.94373
77.24800
77.00000
76.74246
76.47538
42.99535
25.11072
23.16487
20.99964
PPM
190.0
180.0
170.0
160.0
150.0
140.0
130.0
120.0
110.0
100.0
90.0
80.0
70.0
60.0
50.0
40.0
30.0
20.0
10.0
0.0
DFILE hm0553-5tm 13C-1-1.als
COMNT
DATIM 2018-10-16 15:13:34
OBNUC 13C
EXMOD carbon.jxp
OBFRQ 125.77 MHz
OBSET 7.87 KHz
OBFIN 4.21 Hz
POINT 26214
FREQU 31446.54 Hz
SCANS 120
ACQTM 0.8336 sec
PD 2.0000 sec
PW1 3.40 usec
IRNUC 1H
CTEMP 21.2 c
SLVNT CDCL3
EXREF 77.00 ppm
BF 0.12 Hz
RGAIN 60

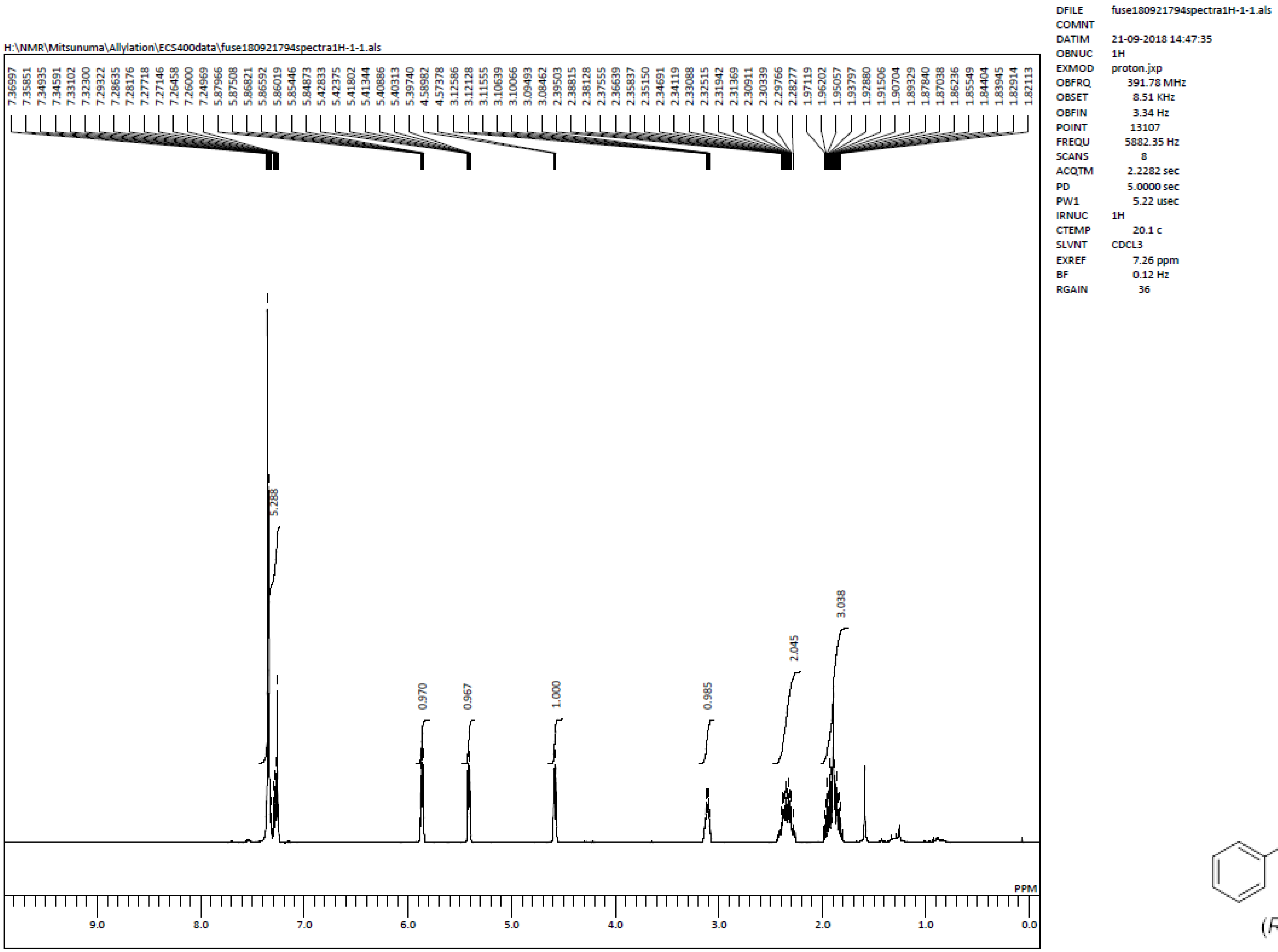
H:\NMR\Mitsunuma\Allylation\ECS400data\fuse180921794spectra1H-1-1.als
7.36997 7.35851 7.34935 7.34591 7.33102 7.32300 7.29322 7.28635 7.28176 7.27718 7.27146 7.26458 7.26000 7.24969 5.87966 5.87508 5.86821 5.86592 5.86019 5.85446 5.84873 5.42833 5.42375 5.41802 5.41344 5.40886 5.40313 5.39740 4.58982 4.57378 3.12586 3.12128 3.11555 3.10639 3.10066 3.09493 3.08462 2.39503 2.38815 2.38128 2.37555 2.36639 2.35837 2.35150 2.34691 2.34119 2.33088 2.32515 2.31942 2.31369 2.30911 2.30339 2.29766 2.28277 1.97119 1.96202 1.95057 1.93797 1.92880 1.91506 1.90704 1.89329 1.87840 1.87038 1.86236 1.85549 1.84404 1.83945 1.82914 1.82113
5.288
0.970
0.967
1.000
0.985
2.045
3.038
PPM
9.0 8.0 7.0 6.0 5.0 4.0 3.0 2.0 1.0 0.0
DFILE fuse180921794spectra1H-1-1.als
COMNT
DATIM 21-09-2018 14:47:35
OBNUC 1H
EXMOD proton.jxp
OBFRQ 391.78 MHz
OBSET 8.51 KHz
OBFIN 3.34 Hz
POINT 13107
FREQU 5882.35 Hz
SCANS 8
ACQTM 2.2282 sec
PD 5.0000 sec
PW1 5.22 usec
IRNUC 1H
CTEMP 20.1 c
SLVNT CDCL3
EXREF 7.26 ppm
BF 0.12 Hz
RGAIN 36

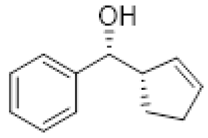
OH

(*R*)-**8h**

H:\NMR\Mitsunuma\Allylation\ECS400data\fuse180921794spectra13C-1-1.als

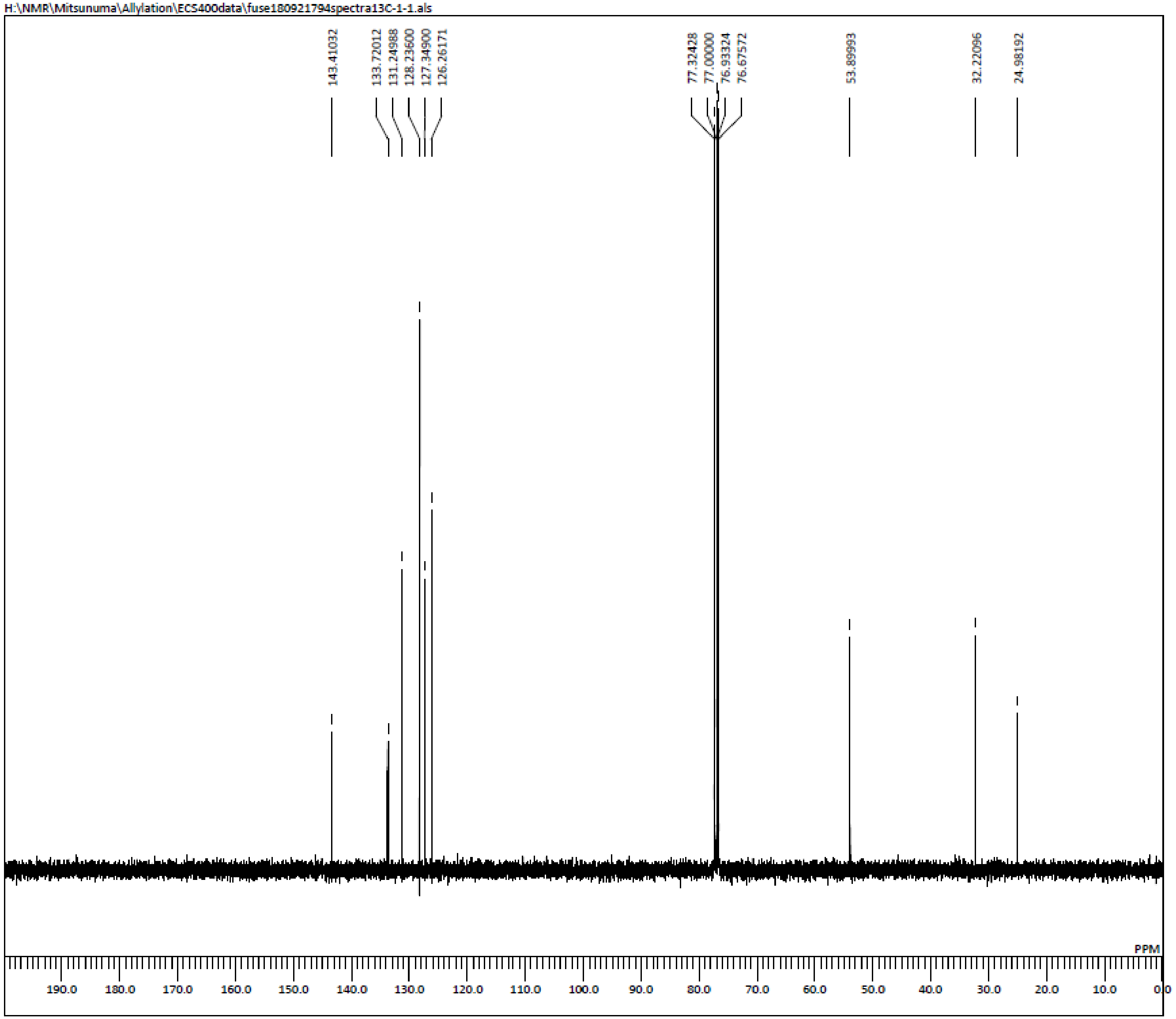

DFILE	fuse180921794spectra13C-1-1.als
COMNT	
DATIM	21-09-2018 14:49:32
OBNUC	13C
EXMOD	carbon.jxp
OBFRQ	98.52 MHz
OBSET	4.64 KHz
OBFIN	8.74 Hz
POINT	26214
FREQU	24630.54 Hz
SCANS	483
ACQTM	1.0643 sec
PD	2.0000 sec
PW1	3.12 usec
IRNUC	1H
CTEMP	20.5 c
SLVNT	CDCL3
EXREF	77.00 ppm
BF	0.12 Hz
RGAIN	60

H:\NMR\Mitsunuma\Allylation\ECS400data\fuse181017hm588spectra1H-1-1.als

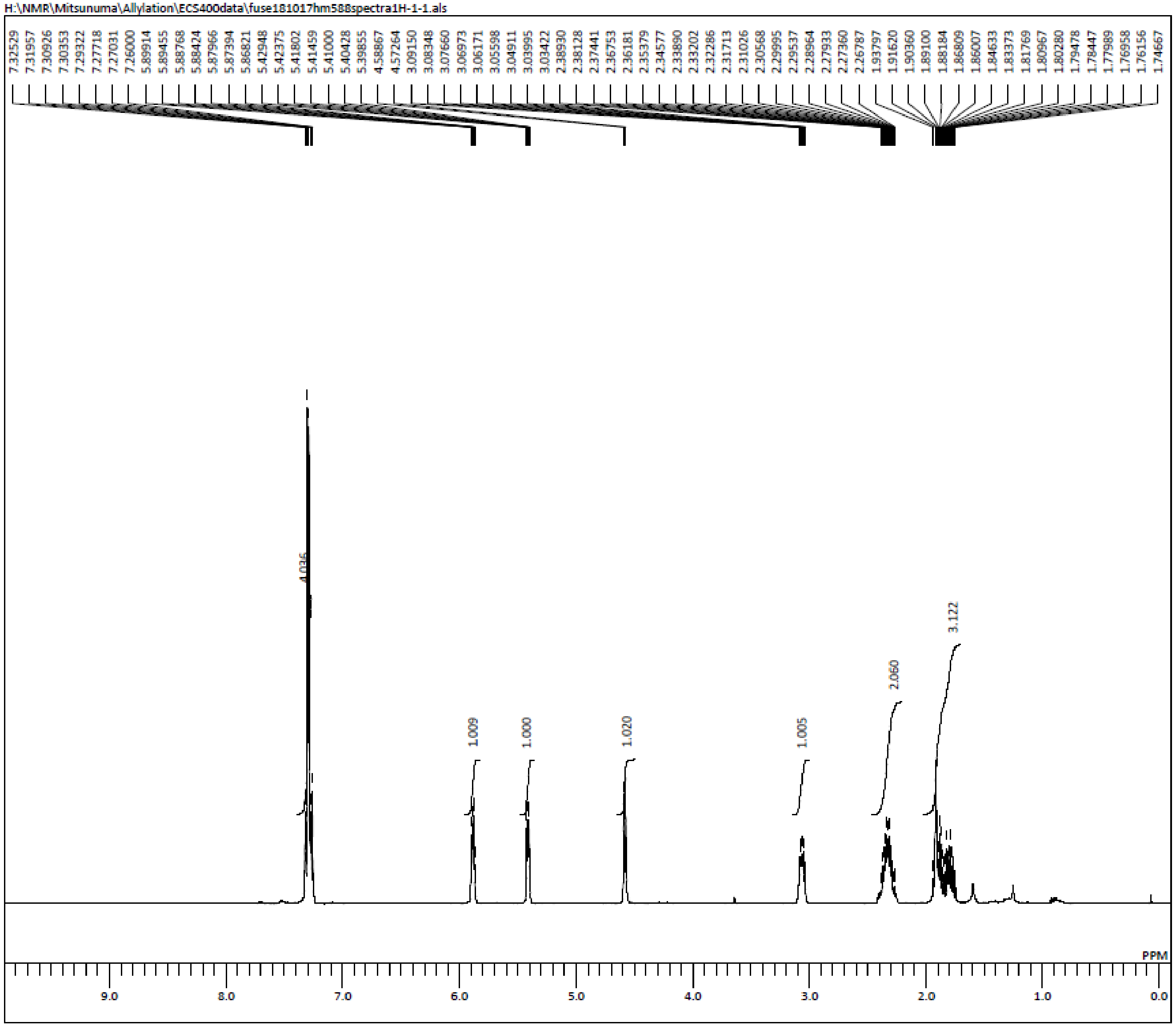

DFILE	fuse181017hm588spectra1H-1-1.als
COMNT	
DATIM	17-10-2018 11:57:09
OBNUC	1H
EXMOD	proton.jxp
OBFRQ	391.78 MHz
OBSET	8.51 KHz
OBFIN	3.34 Hz
POINT	13107
FREQU	5882.35 Hz
SCANS	8
ACQTM	2.2282 sec
PD	8.0000 sec
PW1	5.22 usec
IRNUC	1H
CTEMP	20.1 c
SLVNT	CDCL3
EXREF	7.26 ppm
BF	0.12 Hz
RGAIN	36

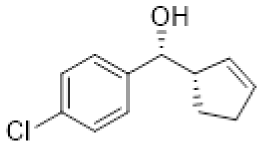

(*R*)-**8i**

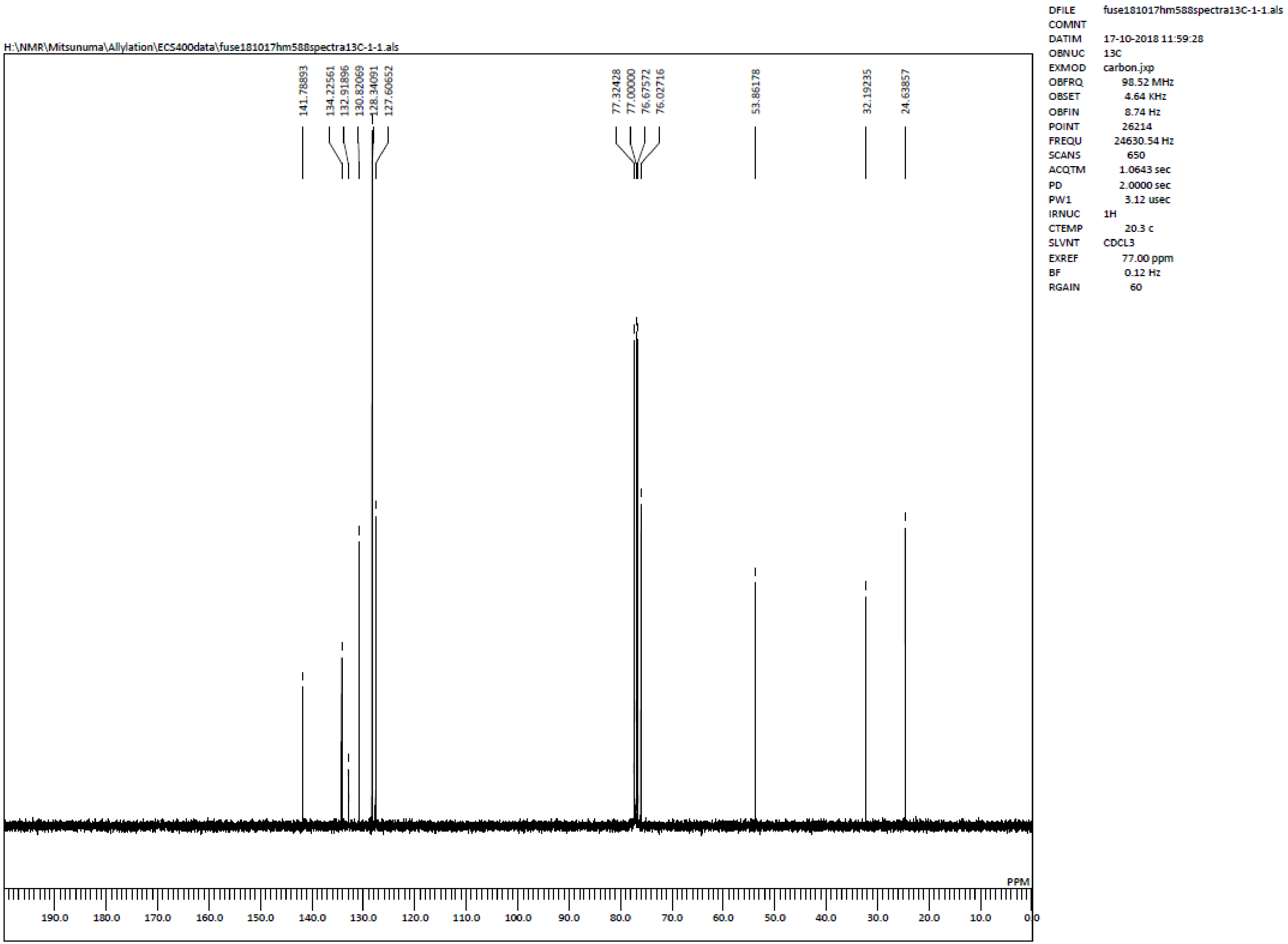
H:\NMR\Mitsunuma\Allylation\ECS400data\fuse181017hm588spectra13C-1-1.als
141.78893
134.22561
132.91896
130.82069
128.34091
127.60652
77.32428
77.00000
76.67572
76.02716
53.86178
32.19235
24.63857
PPM
190.0
180.0
170.0
160.0
150.0
140.0
130.0
120.0
110.0
100.0
90.0
80.0
70.0
60.0
50.0
40.0
30.0
20.0
10.0
0.0
DFILE fuse181017hm588spectra13C-1-1.als
COMNT
DATIM 17-10-2018 11:59:28
OBNUC 13C
EXMOD carbon.jxp
OBFRQ 98.52 MHz
OBSET 4.64 KHz
OBFIN 8.74 Hz
POINT 26214
FREQU 24630.54 Hz
SCANS 650
ACQTM 1.0643 sec
PD 2.0000 sec
PW1 3.12 usec
IRNUC 1H
CTEMP 20.3 c
SLVNT CDCL3
EXREF 77.00 ppm
BF 0.12 Hz
RGAIN 60

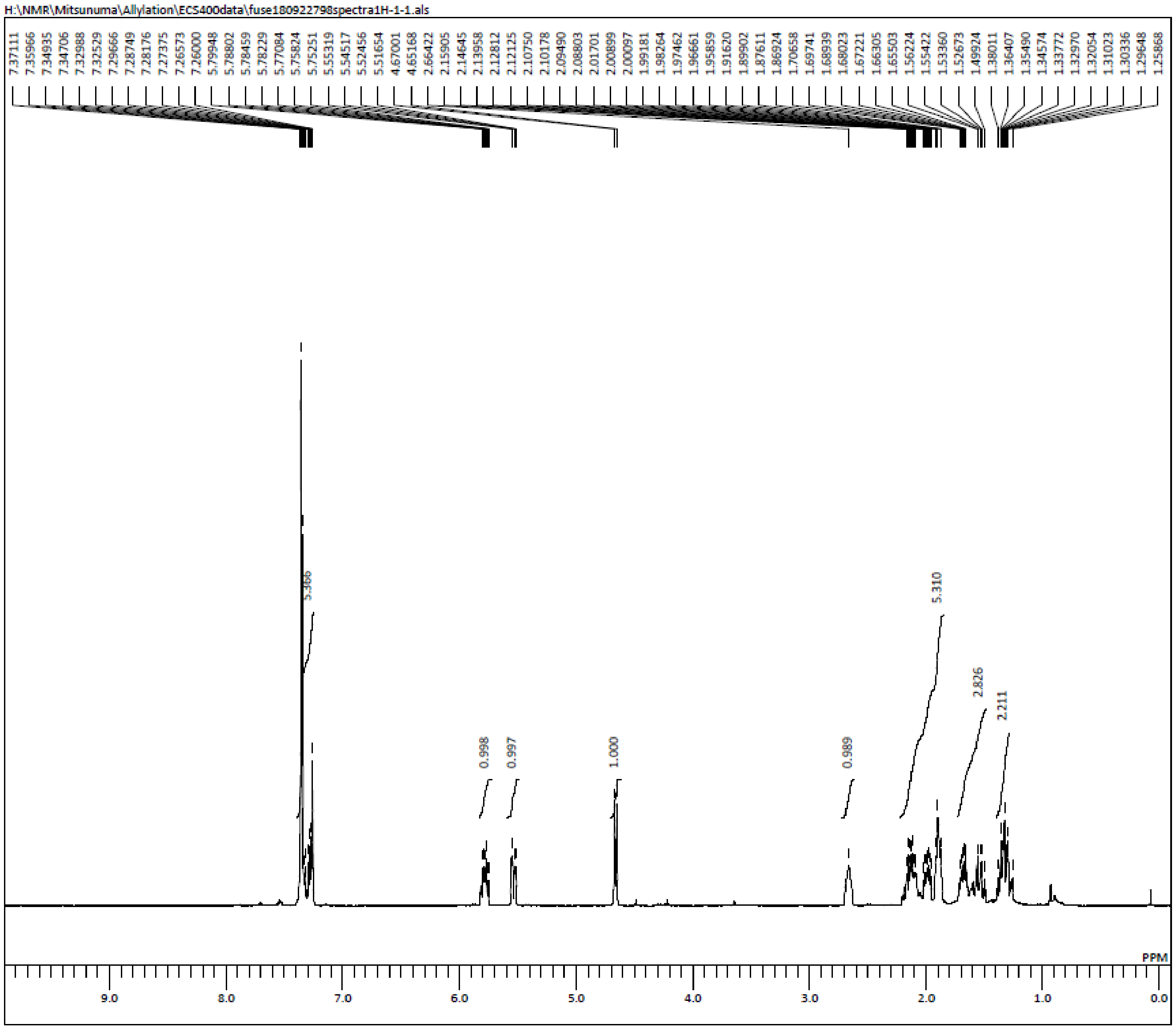

DFILE fuse180922798spectra1H-1-1.als
COMNT
DATIM 22-09-2018 14:07:43
OBNUC 1H
EXMOD proton.jxp
OBFRQ 391.78 MHz
OBSET 8.51 KHz
OBFIN 3.34 Hz
POINT 13107
FREQU 5882.35 Hz
SCANS 8
ACQTM 2.2282 sec
PD 5.0000 sec
PW1 5.22 usec
IRNUC 1H
CTEMP 20.1 c
SLVNT CDCL3
EXREF 7.26 ppm
BF 0.12 Hz
RGAIN 30

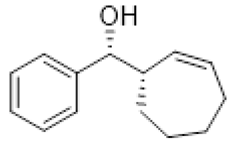

(*R*)-**8j**

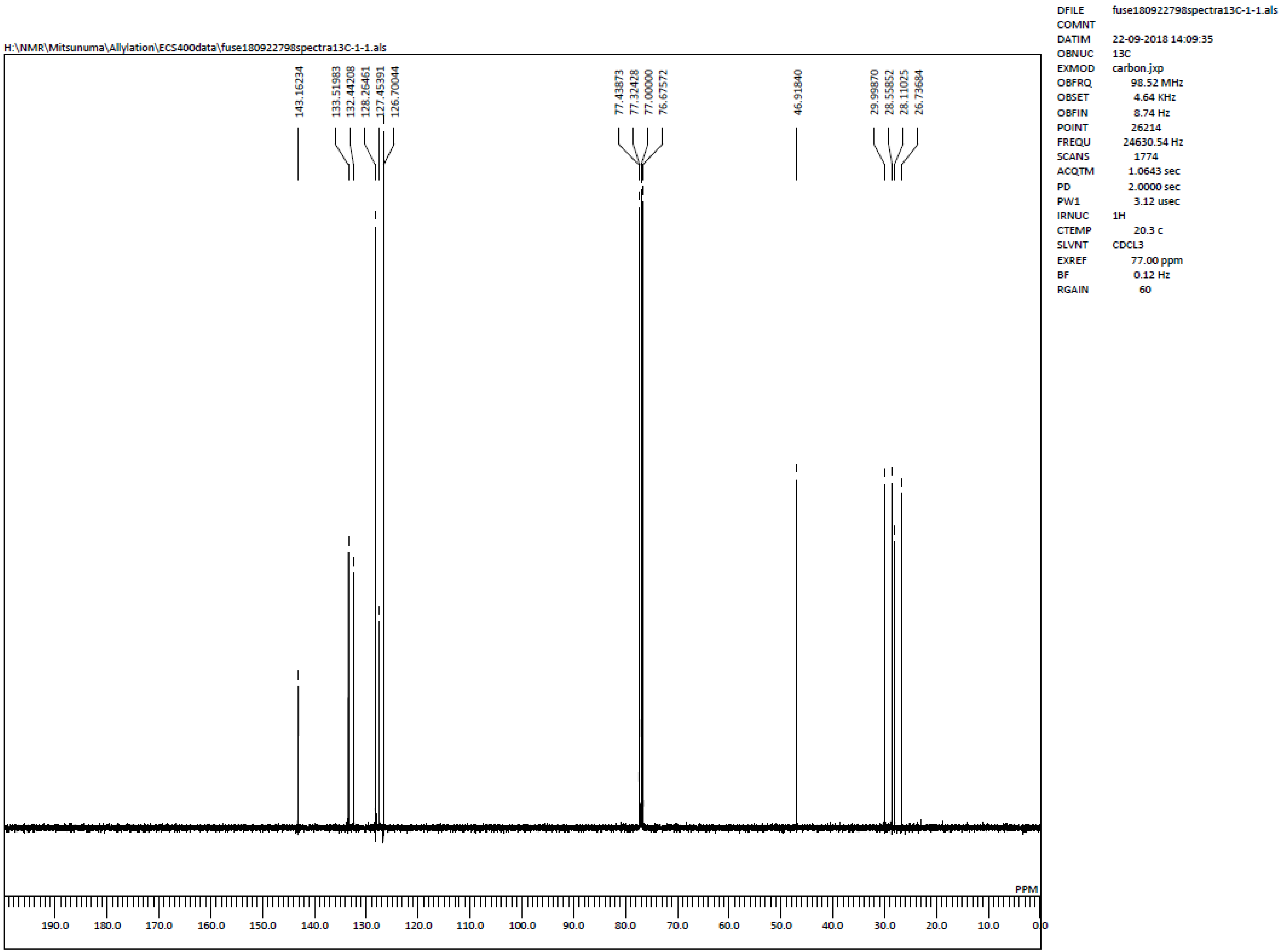
H:\NMR\Mitsunuma\Allylation\ECS400data\fuse180922798spectra13C-1-1.als
143.16234
133.51983
132.44208
128.26461
127.45391
126.70044
77.43873
77.32428
77.00000
76.67572
46.91840
29.99870
28.55852
28.11025
26.73684
PPM
190.0
180.0
170.0
160.0
150.0
140.0
130.0
120.0
110.0
100.0
90.0
80.0
70.0
60.0
50.0
40.0
30.0
20.0
10.0
0.0
DFILE fuse180922798spectra13C-1-1.als
COMNT
DATIM 22-09-2018 14:09:35
OBNUC 13C
EXMOD carbon.jxp
OBFRQ 98.52 MHz
OBSET 4.64 KHz
OBFIN 8.74 Hz
POINT 26214
FREQU 24630.54 Hz
SCANS 1774
ACQTM 1.0643 sec
PD 2.0000 sec
PW1 3.12 usec
IRNUC 1H
CTEMP 20.3 c
SLVNT CDCL3
EXREF 77.00 ppm
BF 0.12 Hz
RGAIN 60

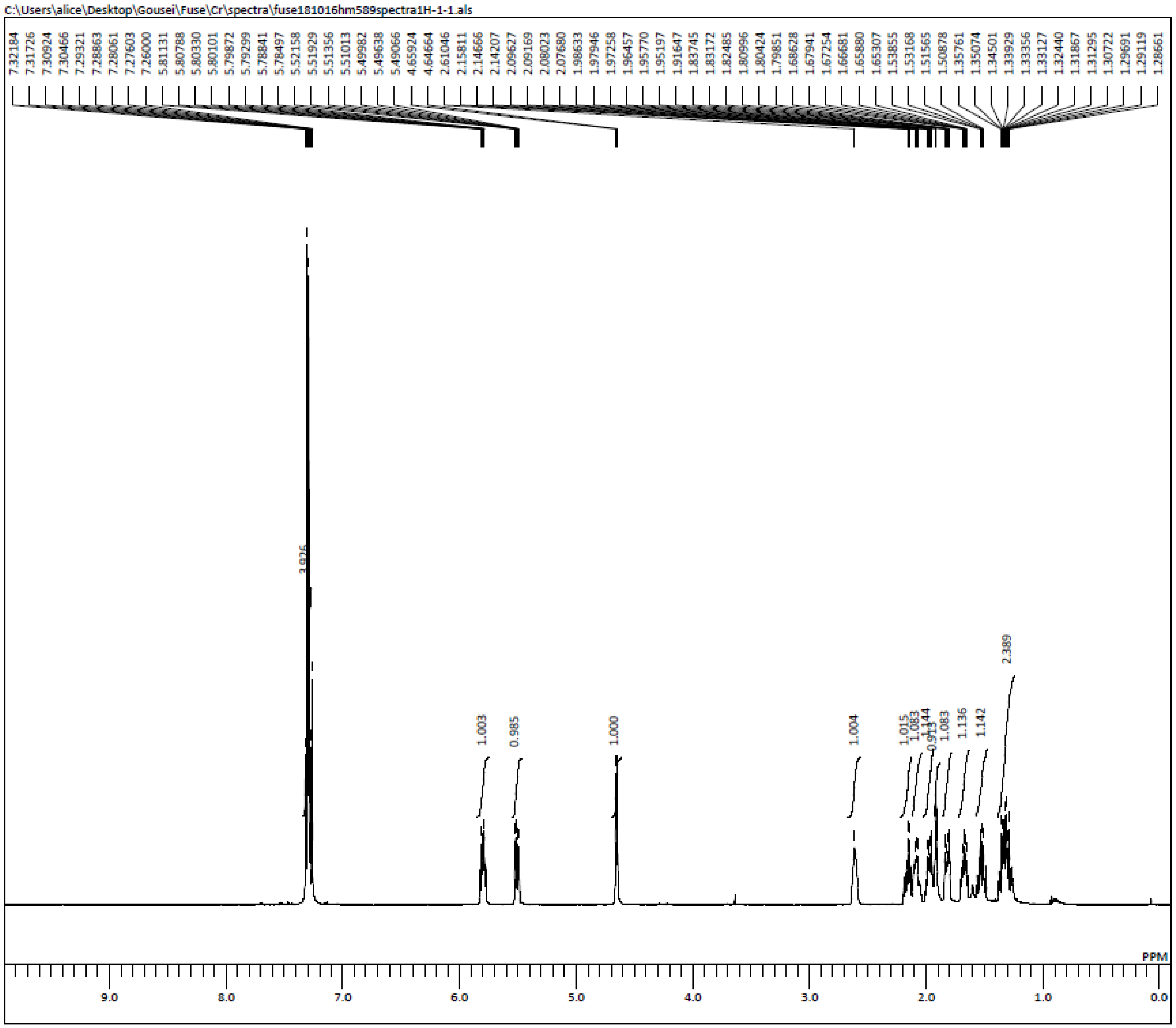

DFILE	fuse181016hm589spectra1H-1-1.als
COMNT	
DATIM	2018-10-16 21:52:32
OBNUC	1H
EXMOD	proton.jxp
OBFRQ	500.16 MHz
OBSET	2.41 KHz
OBFIN	6.01 Hz
POINT	13107
FREQU	7507.51 Hz
SCANS	8
ACQTM	1.7459 sec
PD	5.0000 sec
PW1	5.55 usec
IRNUC	1H
CTEMP	21.8 c
SLVNT	CDCL3
EXREF	7.26 ppm
BF	0.12 Hz
RGAIN	30

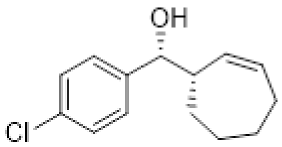

(*R*)-**8k**

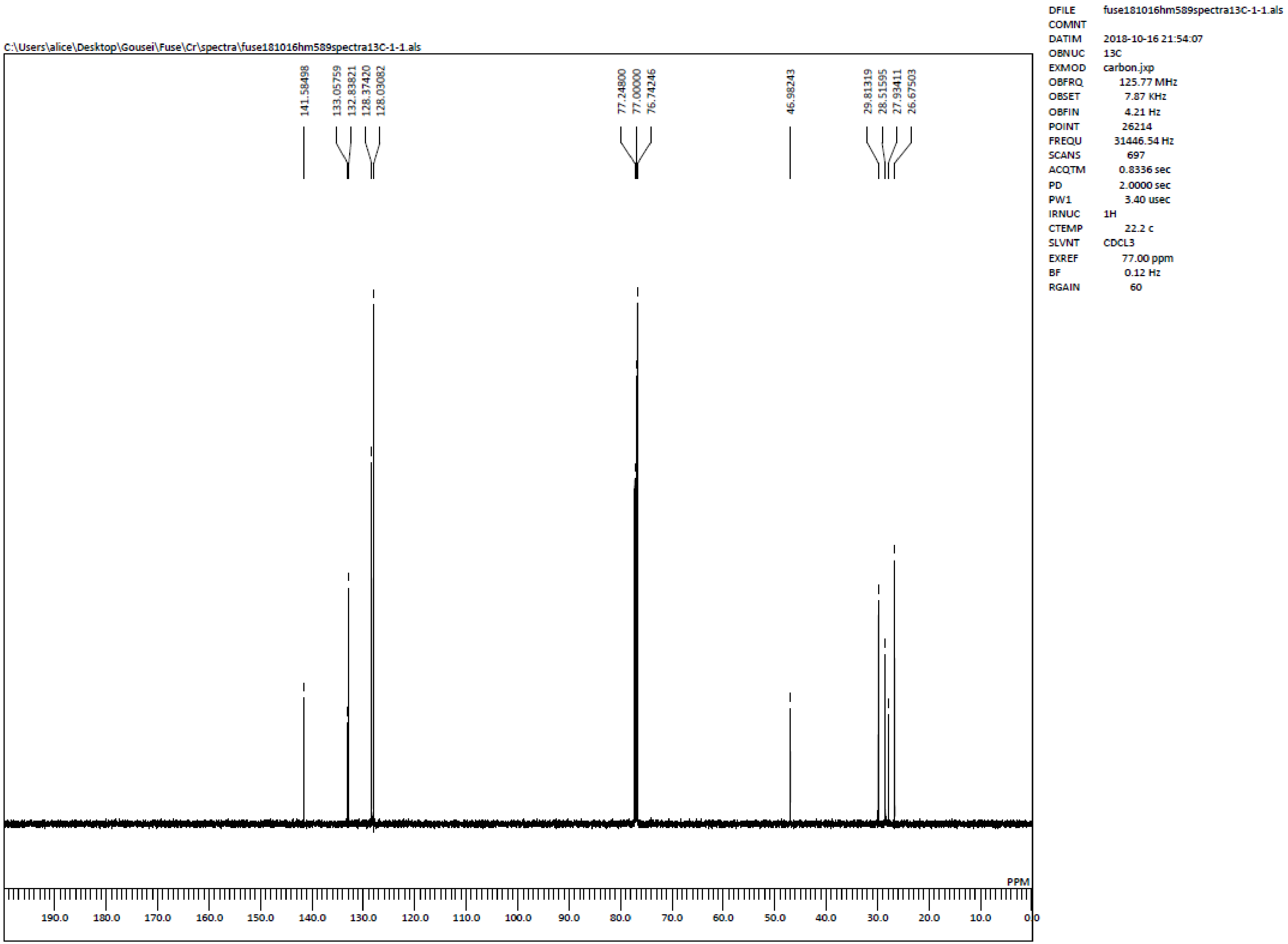
C:\Users\alice\Desktop\Gousei\Fuse\Cr\spectra\fuse181016hm589spectra13C-1-1.als
141.58498
133.05759
132.83821
128.37420
128.03082
77.24800
77.00000
76.74246
46.98243
29.81319
28.51595
27.93411
26.67503
PPM
190.0
180.0
170.0
160.0
150.0
140.0
130.0
120.0
110.0
100.0
90.0
80.0
70.0
60.0
50.0
40.0
30.0
20.0
10.0
0.0
DFILE fuse181016hm589spectra13C-1-1.als
COMNT
DATIM 2018-10-16 21:54:07
OBNUC 13C
EXMOD carbon.jxp
OBFRQ 125.77 MHz
OBSET 7.87 KHz
OBFIN 4.21 Hz
POINT 26214
FREQU 31446.54 Hz
SCANS 697
ACQTM 0.8336 sec
PD 2.0000 sec
PW1 3.40 usec
IRNUC 1H
CTEMP 22.2 c
SLVNT CDCL3
EXREF 77.00 ppm
BF 0.12 Hz
RGAIN 60

C:\Users\alice\Desktop\Gousei\mitsunuma\hm0290-1column-1-1.als

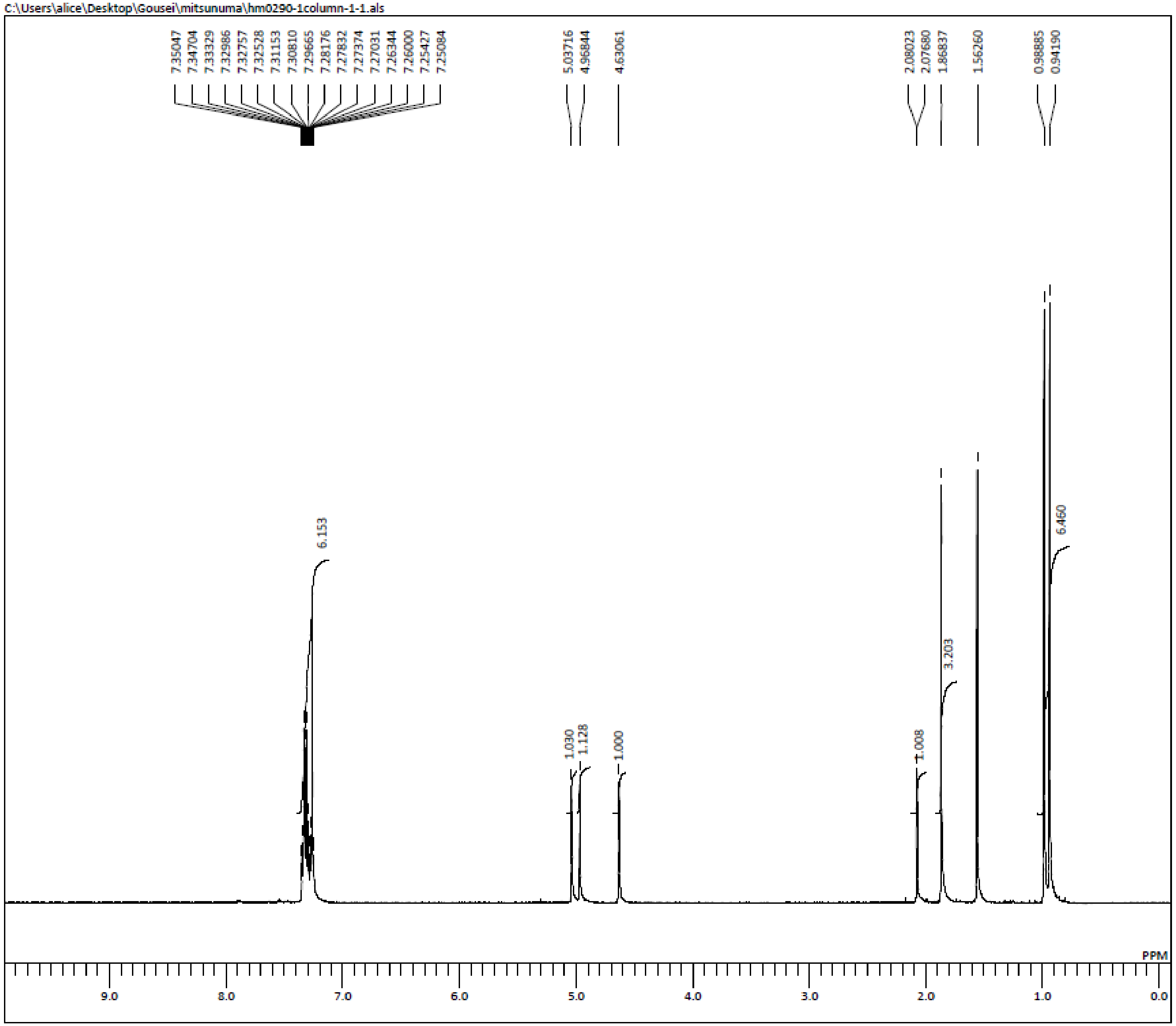

DFILE	hm0290-1column-1-1.als
COMNT	
DATIM	2018-06-29 18:17:57
OBNUC	1H
EXMOD	proton.jxp
OBFRQ	500.16 MHz
OBSET	2.41 KHz
OBFIN	6.01 Hz
POINT	13107
FREQU	7507.51 Hz
SCANS	8
ACQTM	1.7459 sec
PD	5.0000 sec
PW1	5.55 usec
IRNUC	1H
CTEMP	21.6 c
SLVNT	CDCL3
EXREF	7.26 ppm
BF	0.12 Hz
RGAIN	32

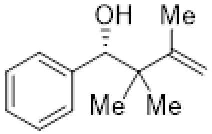

(*S*)-8l

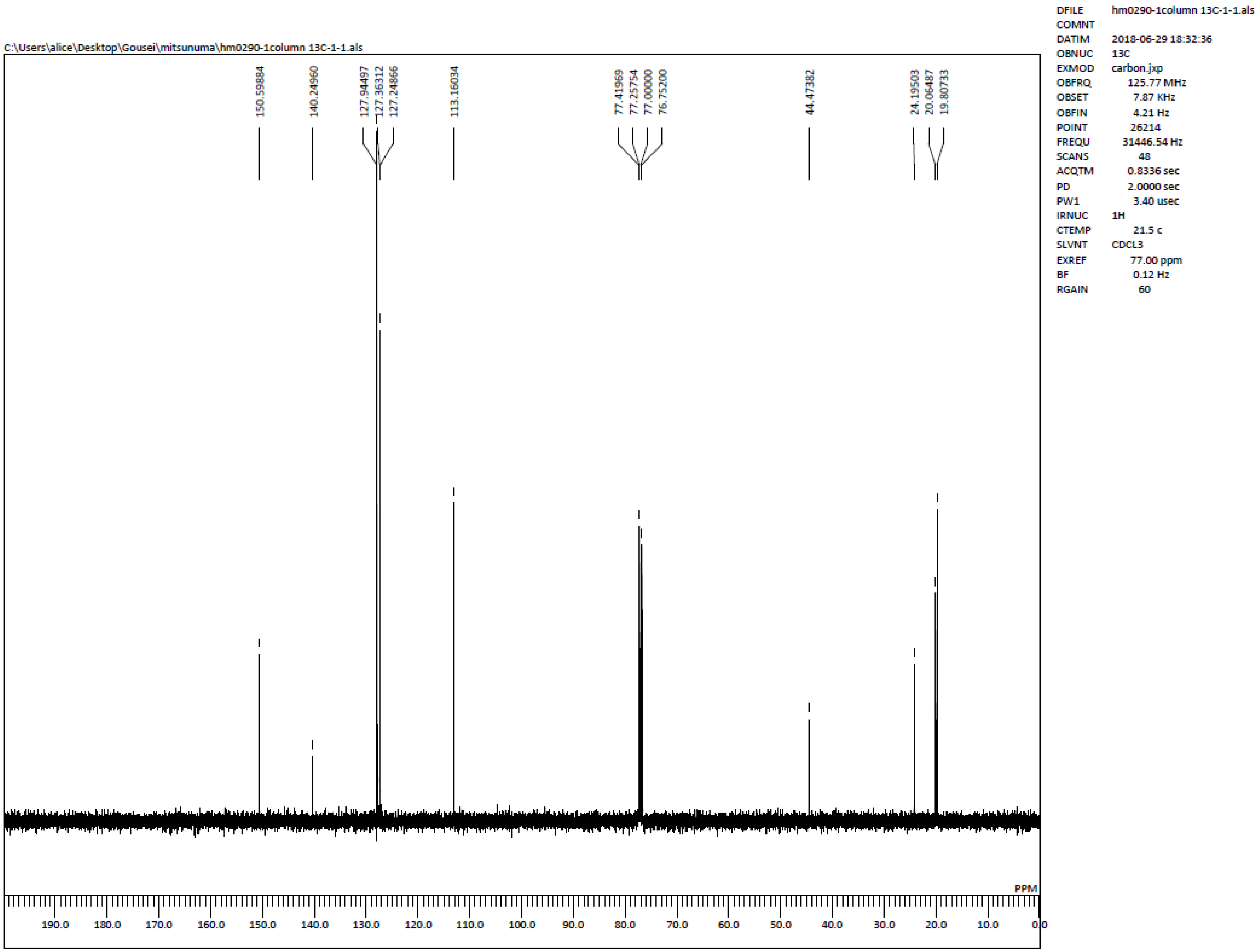
C:\Users\alice\Desktop\Gousei\mitsunuma\hm0290-1column 13C-1-1.als
150.59884
140.24960
127.94497
127.36312
127.24866
113.16034
77.41969
77.25754
77.00000
76.75200
44.47382
24.19503
20.06487
19.80733
PPM
190.0 180.0 170.0 160.0 150.0 140.0 130.0 120.0 110.0 100.0 90.0 80.0 70.0 60.0 50.0 40.0 30.0 20.0 10.0 0.0
DFILE hm0290-1column 13C-1-1.als
COMNT
DATIM 2018-06-29 18:32:36
OBNUC 13C
EXMOD carbon.jxp
OBFRQ 125.77 MHz
OBSET 7.87 KHz
OBFIN 4.21 Hz
POINT 26214
FREQU 31446.54 Hz
SCANS 48
ACQTM 0.8336 sec
PD 2.0000 sec
PW1 3.40 usec
IRNUC 1H
CTEMP 21.5 c
SLVNT CDCL3
EXREF 77.00 ppm
BF 0.12 Hz
RGAIN 60

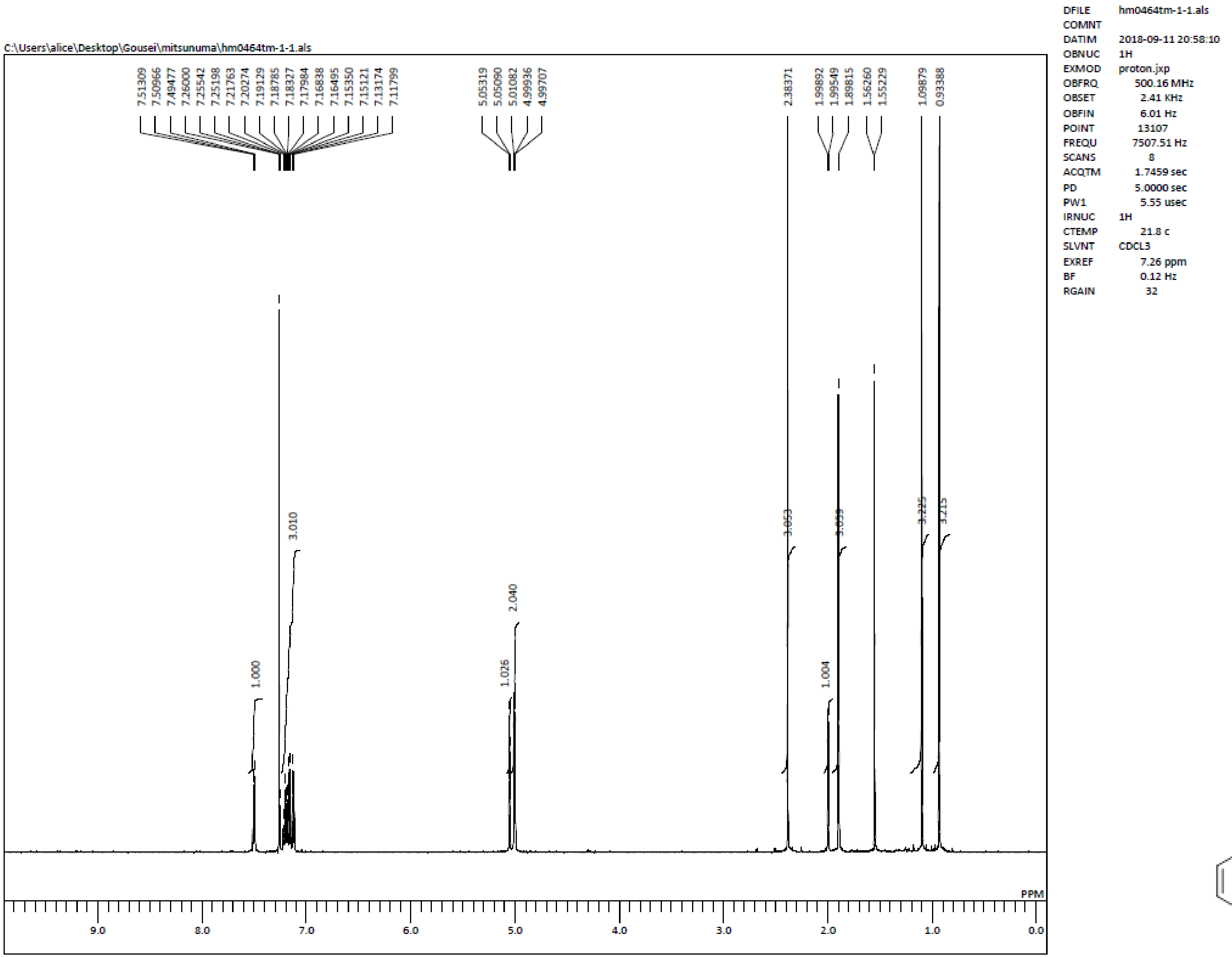
C:\Users\alice\Desktop\Gousei\mitsunuma\hm0464tm-1-1.als
7.51309
7.50966
7.49477
7.26000
7.25542
7.25198
7.21763
7.20274
7.19129
7.18785
7.18327
7.17984
7.16838
7.16495
7.15350
7.15121
7.13174
7.11799
5.05319
5.05090
5.01082
4.99936
4.99707
2.38371
1.99892
1.99549
1.89815
1.56260
1.55229
1.09879
0.93388
1.000
3.010
1.026
2.040
1.004
PPM
9.0
8.0
7.0
6.0
5.0
4.0
3.0
2.0
1.0
0.0
DFILE hm0464tm-1-1.als
COMNT
DATIM 2018-09-11 20:58:10
OBNUC 1H
EXMOD proton.jxp
OBFRQ 500.16 MHz
OBSET 2.41 KHz
OBFIN 6.01 Hz
POINT 13107
FREQU 7507.51 Hz
SCANS 8
ACQTM 1.7459 sec
PD 5.0000 sec
PW1 5.55 usec
IRNUC 1H
CTEMP 21.8 c
SLVNT CDCL3
EXREF 7.26 ppm
BF 0.12 Hz
RGAIN 32

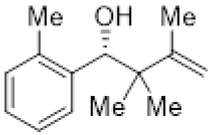
Me
OH
Me
Me
Me

(*R*)-**8m**

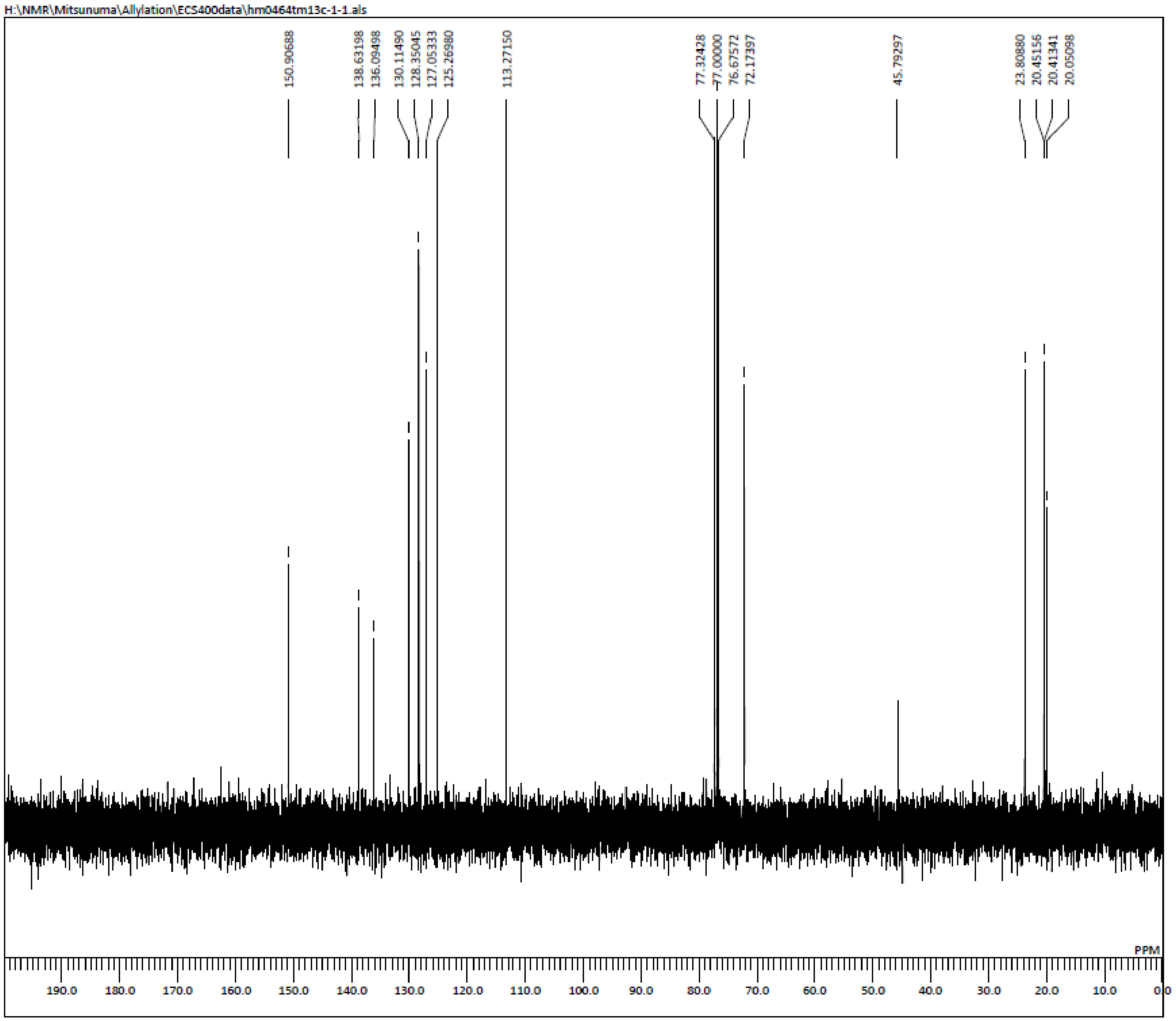

DFILE	hm0464tm13c-1-1.als
COMNT	
DATIM	11-09-2018 22:50:17
OBNUC	13C
EXMOD	carbon.jxp
OBFRQ	98.52 MHz
OBSET	4.64 KHz
OBFIN	8.74 Hz
POINT	26214
FREQU	24630.54 Hz
SCANS	20
ACQTM	1.0643 sec
PD	2.0000 sec
PW1	3.12 usec
IRNUC	1H
CTEMP	20.5 c
SLVNT	CDCL3
EXREF	77.00 ppm
BF	0.12 Hz
RGAIN	60

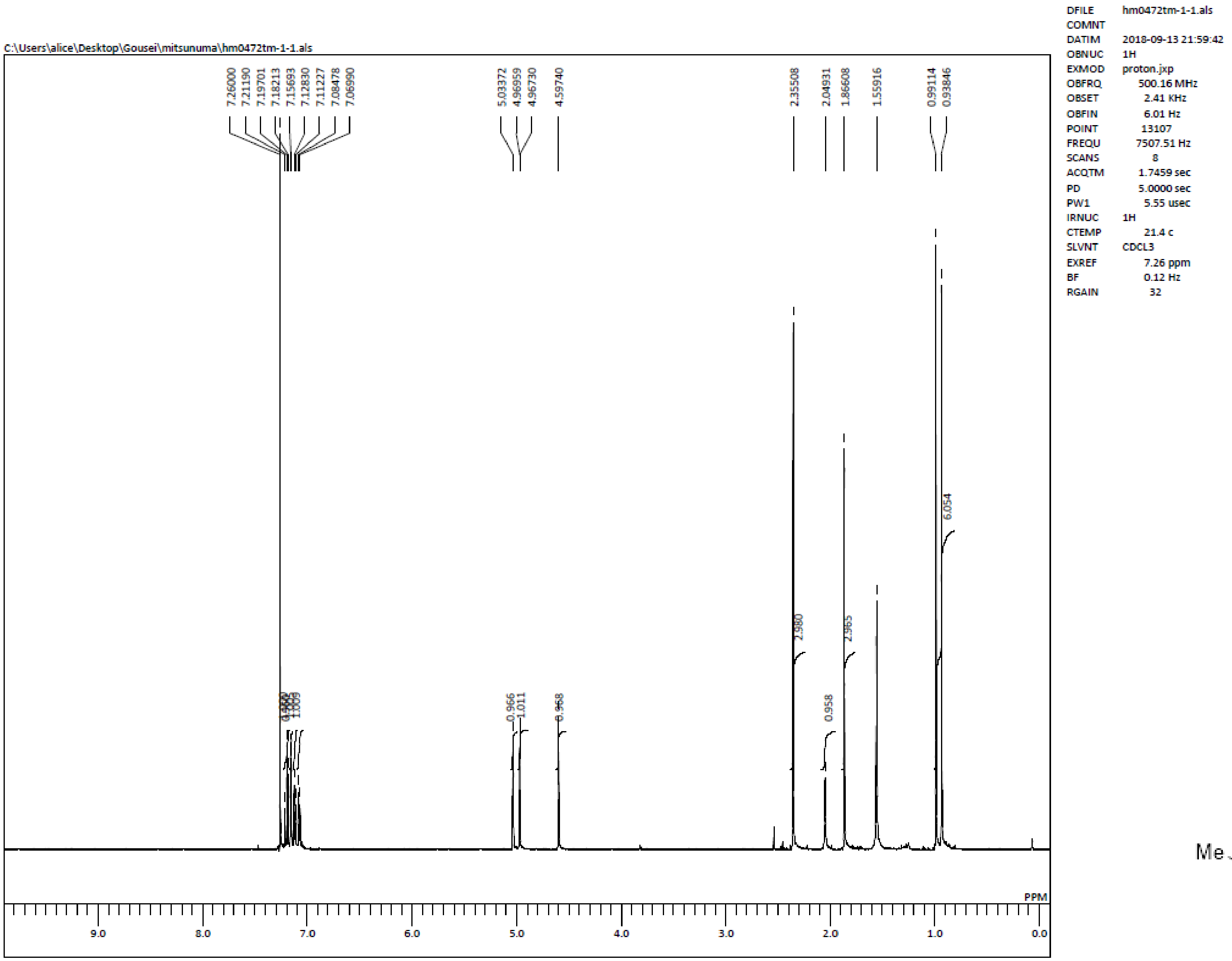
C:\Users\alice\Desktop\Gousei\mitsunuma\hm0472tm-1-1.als
7.26000
7.21190
7.19701
7.18213
7.15693
7.12830
7.11227
7.08478
7.06990
5.03372
4.96959
4.96730
4.59740
2.35508
2.04931
1.86608
1.55916
0.99114
0.93846
0.966
1.011
0.958
2.980
2.965
6.054
PPM
9.0
8.0
7.0
6.0
5.0
4.0
3.0
2.0
1.0
0.0
DFILE hm0472tm-1-1.als
COMNT
DATIM 2018-09-13 21:59:42
OBNUC 1H
EXMOD proton.jxp
OBFRQ 500.16 MHz
OBSET 2.41 KHz
OBFIN 6.01 Hz
POINT 13107
FREQU 7507.51 Hz
SCANS 8
ACQTM 1.7459 sec
PD 5.0000 sec
PW1 5.55 usec
IRNUC 1H
CTEMP 21.4 c
SLVNT CDCL3
EXREF 7.26 ppm
BF 0.12 Hz
RGAIN 32

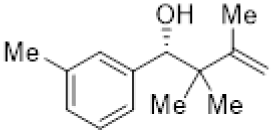
OH
Me
Me
Me
Me

(*R*)-**8n**

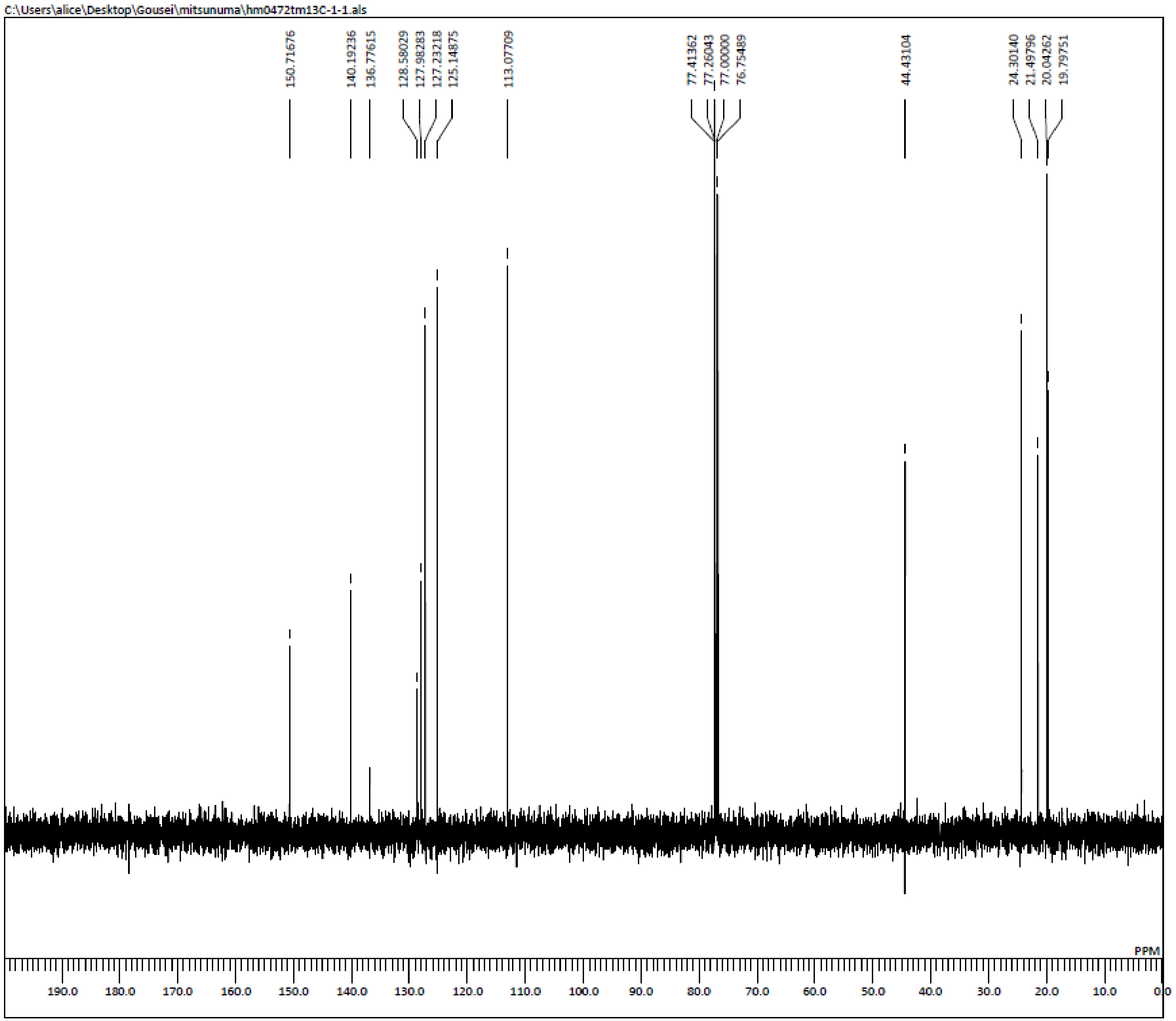

DFILE	hm0472tm13C-1-1.als
COMNT	
DATIM	2018-09-13 22:16:04
OBNUC	13C
EXMOD	carbon.jxp
OBFRQ	125.77 MHz
OBSET	1.58 KHz
OBFIN	5.95 Hz
POINT	26214
FREQU	50505.05 Hz
SCANS	24
ACQTM	0.5190 sec
PD	2.0000 sec
PW1	3.40 usec
IRNUC	1H
CTEMP	21.3 c
SLVNT	CDCL3
EXREF	77.00 ppm
BF	0.12 Hz
RGAIN	58

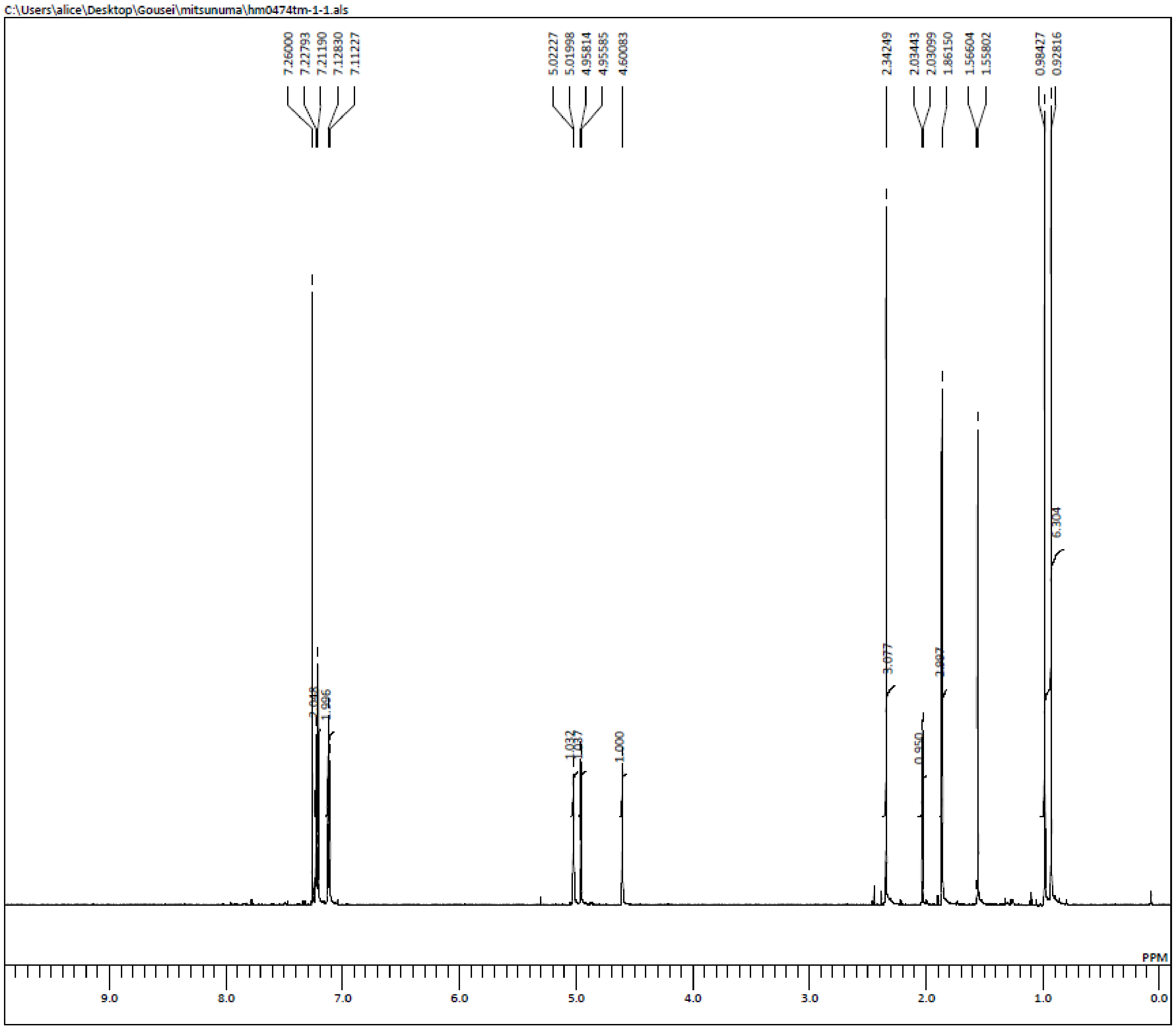

DFILE	hm0474tm-1-1.als
COMNT	
DATIM	2018-09-13 22:03:22
OBNUC	1H
EXMOD	proton.jxp
OBFRQ	500.16 MHz
OBSET	2.41 KHz
OBFIN	6.01 Hz
POINT	13107
FREQU	7507.51 Hz
SCANS	8
ACQTM	1.7459 sec
PD	5.0000 sec
PW1	5.55 usec
IRNUC	1H
CTEMP	21.4 c
SLVNT	CDCL3
EXREF	7.26 ppm
BF	0.12 Hz
RGAIN	34

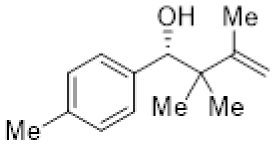

(*S*)-**8o**

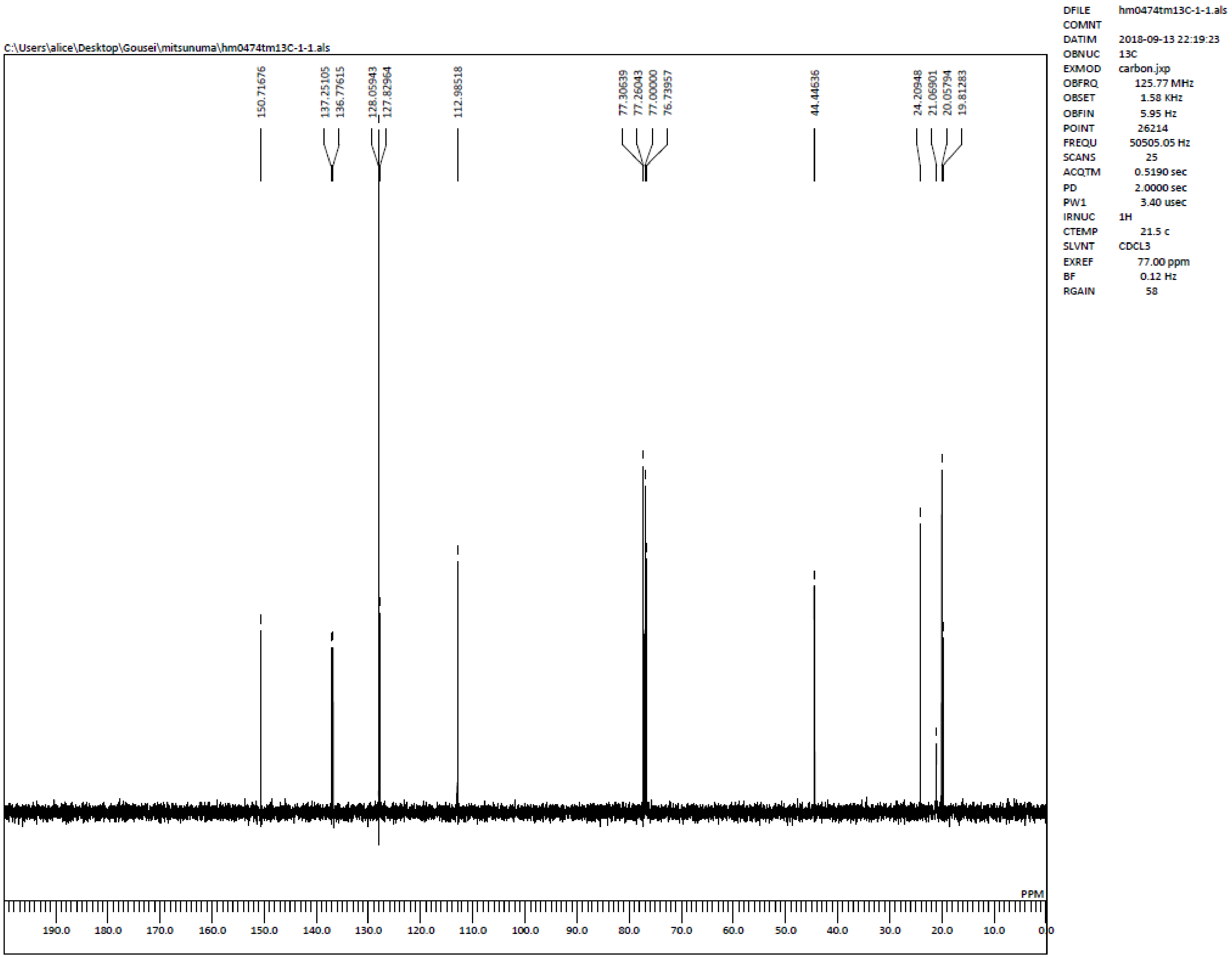

C:\Users\alice\Desktop\Gousei\mitsunuma\hm0474tm13C-1-1.als
150.71676
137.25105
136.77615
128.05943
127.82964
112.98518
77.30639
77.26043
77.00000
76.73957
44.44636
24.20948
21.06901
20.05794
19.81283
PPM
190.0 180.0 170.0 160.0 150.0 140.0 130.0 120.0 110.0 100.0 90.0 80.0 70.0 60.0 50.0 40.0 30.0 20.0 10.0 0.0
DFILE hm0474tm13C-1-1.als
COMNT
DATIM 2018-09-13 22:19:23
OBNUC 13C
EXMOD carbon.jxp
OBFRQ 125.77 MHz
OBSET 1.58 KHz
OBFIN 5.95 Hz
POINT 26214
FREQU 50505.05 Hz
SCANS 25
ACQTM 0.5190 sec
PD 2.0000 sec
PW1 3.40 usec
IRNUC 1H
CTEMP 21.5 c
SLVNT CDCL3
EXREF 77.00 ppm
BF 0.12 Hz
RGAIN 58

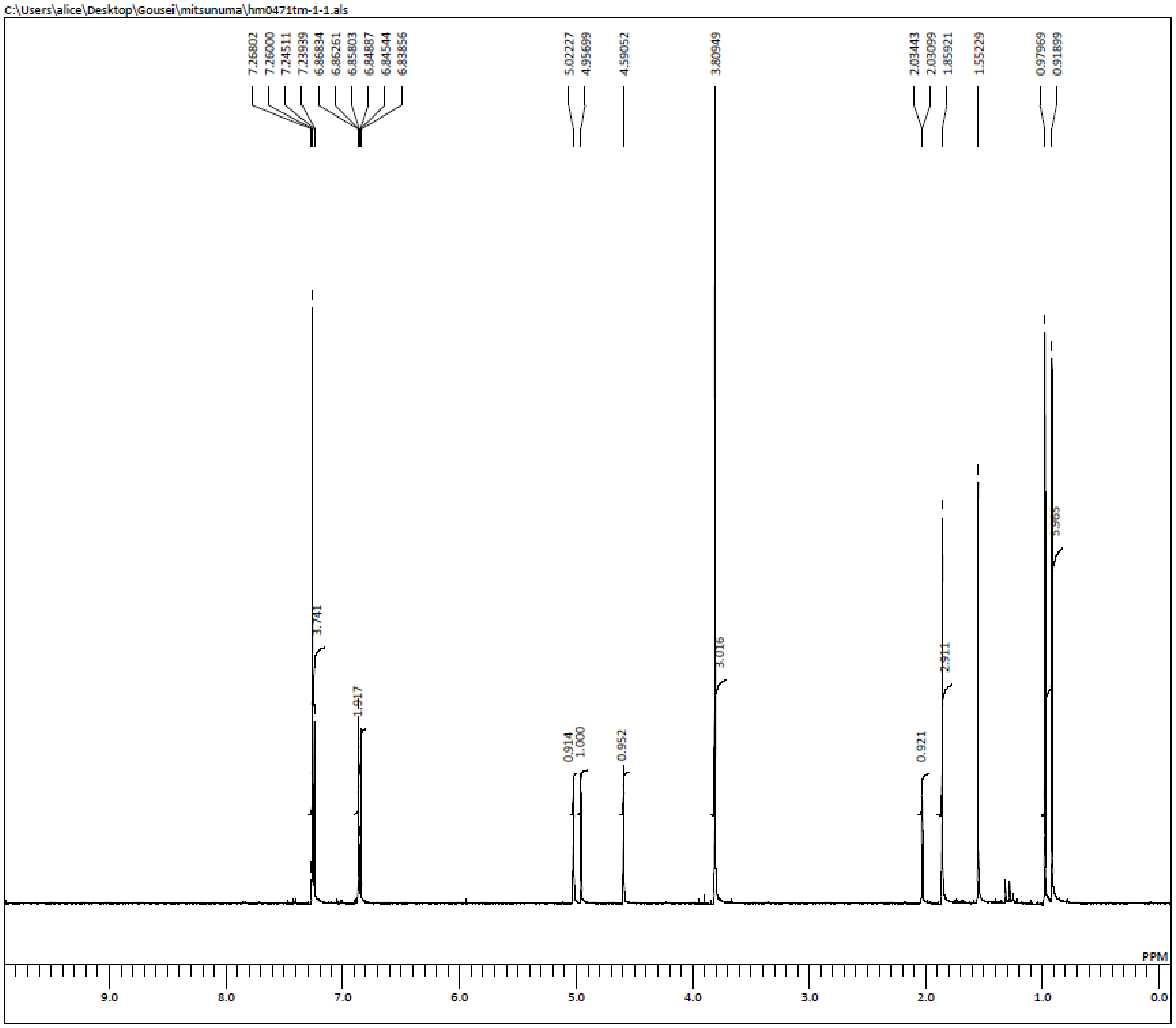

DFILE hm0471tm-1-1.als
COMNT
DATIM 2018-09-11 21:09:39
OBNUC 1H
EXMOD proton.jxp
OBFRQ 500.16 MHz
OBSET 2.41 KHz
OBFIN 6.01 Hz
POINT 13107
FREQU 7507.51 Hz
SCANS 8
ACQTM 1.7459 sec
PD 5.0000 sec
PW1 5.55 usec
IRNUC 1H
CTEMP 21.6 c
SLVNT CDCL3
EXREF 7.26 ppm
BF 0.12 Hz
RGAIN 32

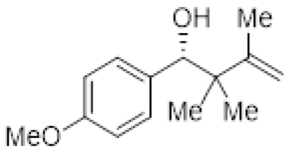

(*S*)-**8p**

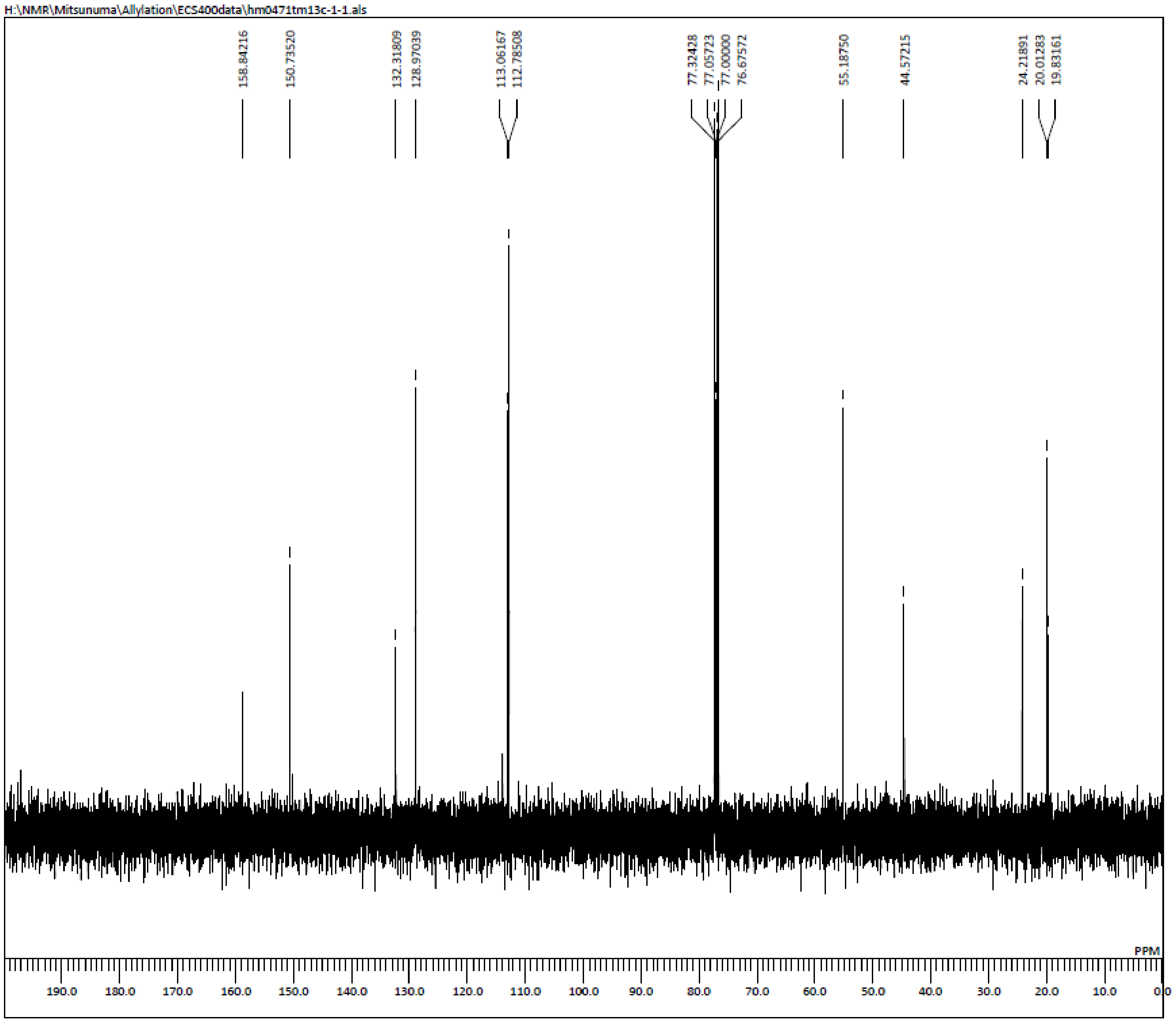

DFILE	hm0471tm13c-1-1.als
COMNT	
DATIM	11-09-2018 22:58:34
OBNUC	13C
EXMOD	carbon.jxp
OBFRQ	98.52 MHz
OBSET	4.64 KHz
OBFIN	8.74 Hz
POINT	26214
FREQU	24630.54 Hz
SCANS	32
ACQTM	1.0643 sec
PD	2.0000 sec
PW1	3.12 usec
IRNUC	1H
CTEMP	20.5 c
SLVNT	CDCL3
EXREF	77.00 ppm
BF	0.12 Hz
RGAIN	60

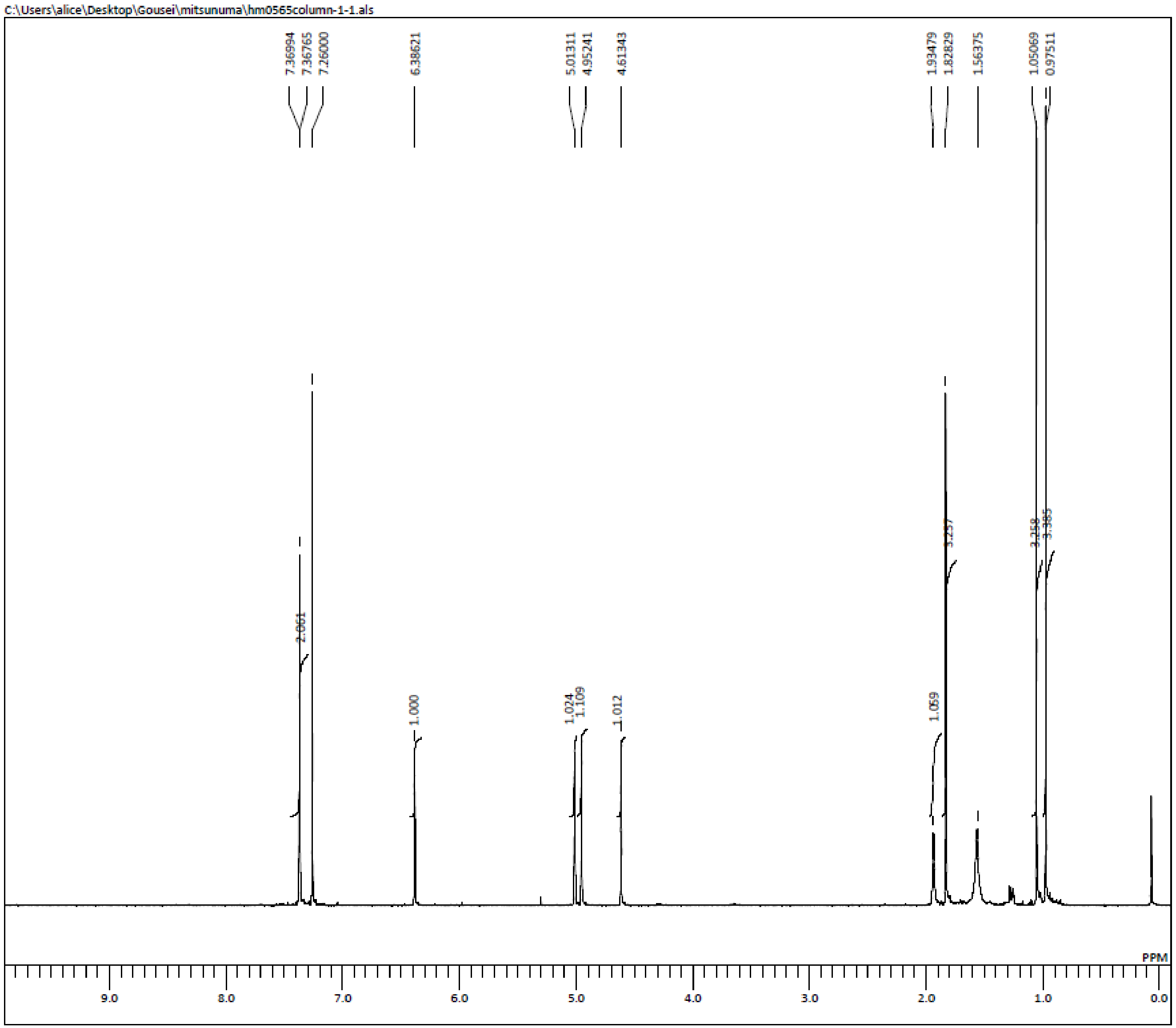

DFILE hm0565column-1-1.als
COMNT
DATIM 2018-10-05 22:27:10
OBNUC 1H
EXMOD proton.jxp
OBFRQ 500.16 MHz
OBSET 2.41 KHz
OBFIN 6.01 Hz
POINT 13107
FREQU 7507.51 Hz
SCANS 8
ACQTM 1.7459 sec
PD 5.0000 sec
PW1 5.55 usec
IRNUC 1H
CTEMP 21.6 c
SLVNT CDCL3
EXREF 7.26 ppm
BF 0.12 Hz
RGAIN 34

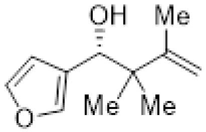

(*R*)-**8q**

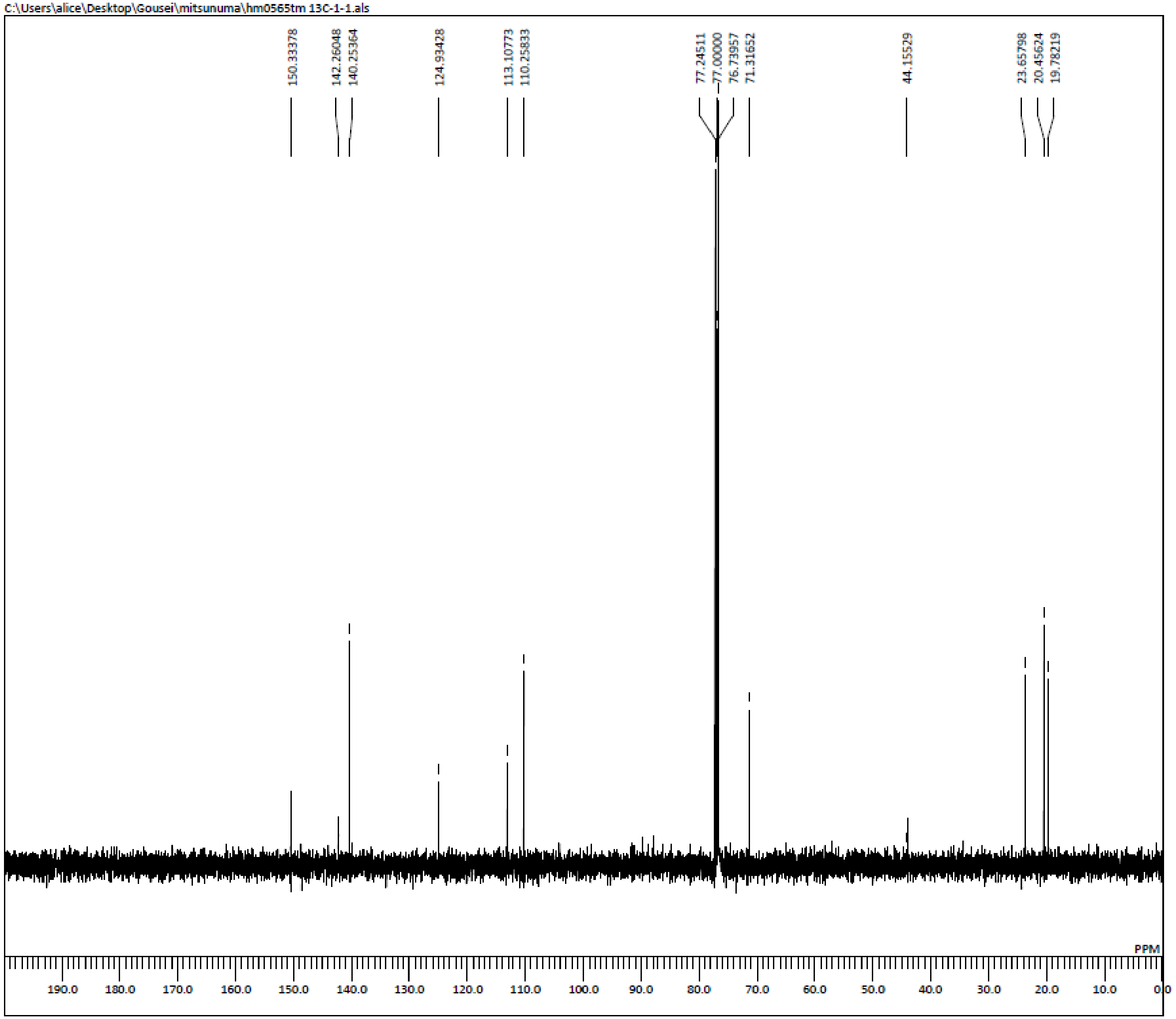

DFILE	hm0565tm 13C-1-1.als
COMNT	
DATIM	2018-10-05 23:09:42
OBNUC	13C
EXMOD	carbon.jxp
OBFRQ	125.77 MHz
OBSET	1.58 KHz
OBFIN	5.95 Hz
POINT	26214
FREQU	50505.05 Hz
SCANS	60
ACQTM	0.5190 sec
PD	2.0000 sec
PW1	3.40 usec
IRNUC	1H
CTEMP	21.7 c
SLVNT	CDCL3
EXREF	77.00 ppm
BF	0.12 Hz
RGAIN	58

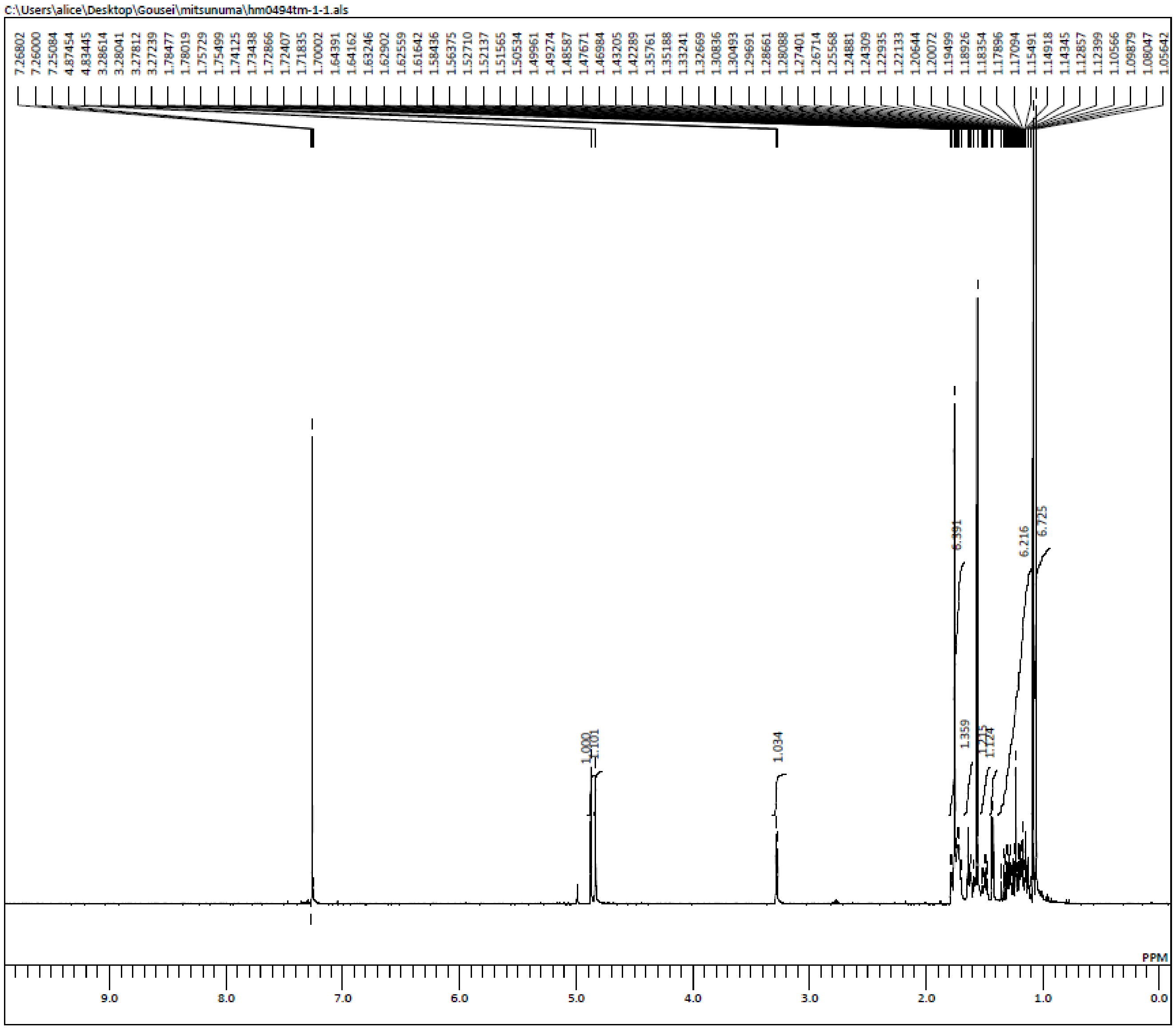

DFILE	hm0494tm-1-1.als
COMNT	
DATIM	2018-09-16 23:46:03
OBNUC	1H
EXMOD	proton.jxp
OBFRQ	500.16 MHz
OBSET	2.41 KHz
OBFIN	6.01 Hz
POINT	13107
FREQU	7507.51 Hz
SCANS	8
ACQTM	1.7459 sec
PD	5.0000 sec
PW1	5.55 usec
IRNUC	1H
CTEMP	21.7 c
SLVNT	CDCL3
EXREF	7.26 ppm
BF	0.12 Hz
RGAIN	32

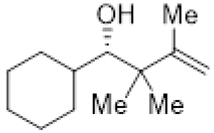

(*S*)-**8r**

H:\NMR\Mitsunuma\Allylation\ECS400data\hm0494tm 13C-1-1.als

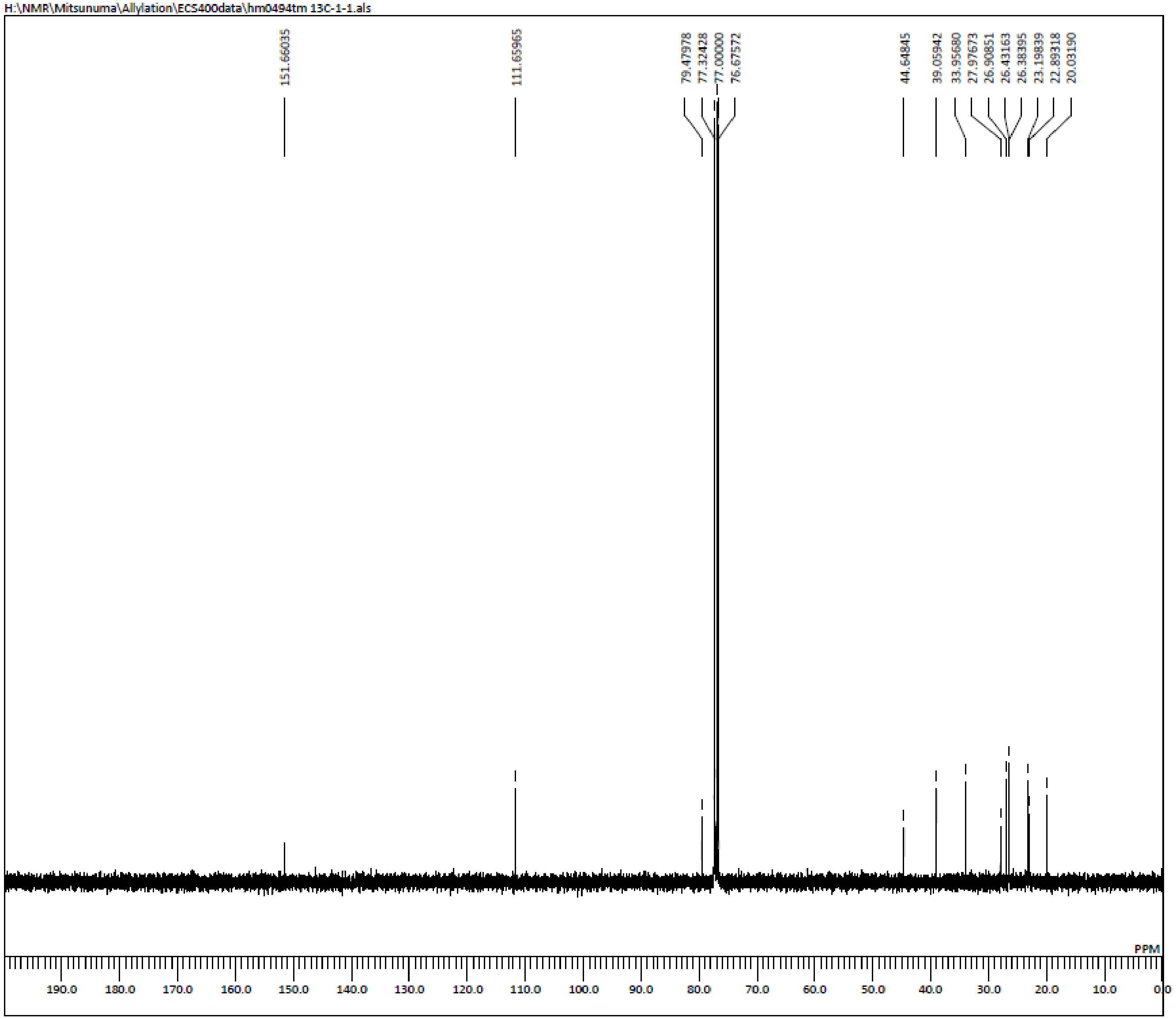

DFILE	hm0494tm 13C-1-1.als
COMNT	
DATIM	17-09-2018 19:04:55
OBNUC	13C
EXMOD	carbon.jxp
OBFRQ	98.52 MHz
OBSET	4.64 KHz
OBFIN	8.74 Hz
POINT	26214
FREQU	24630.54 Hz
SCANS	640
ACQTM	1.0643 sec
PD	2.0000 sec
PW1	3.12 usec
IRNUC	1H
CTEMP	20.5 c
SLVNT	CDCL3
EXREF	77.00 ppm
BF	0.12 Hz
RGAIN	60

C:\Users\alice\Desktop\Gousei\mitsunuma\hm0485tm-1-1.als

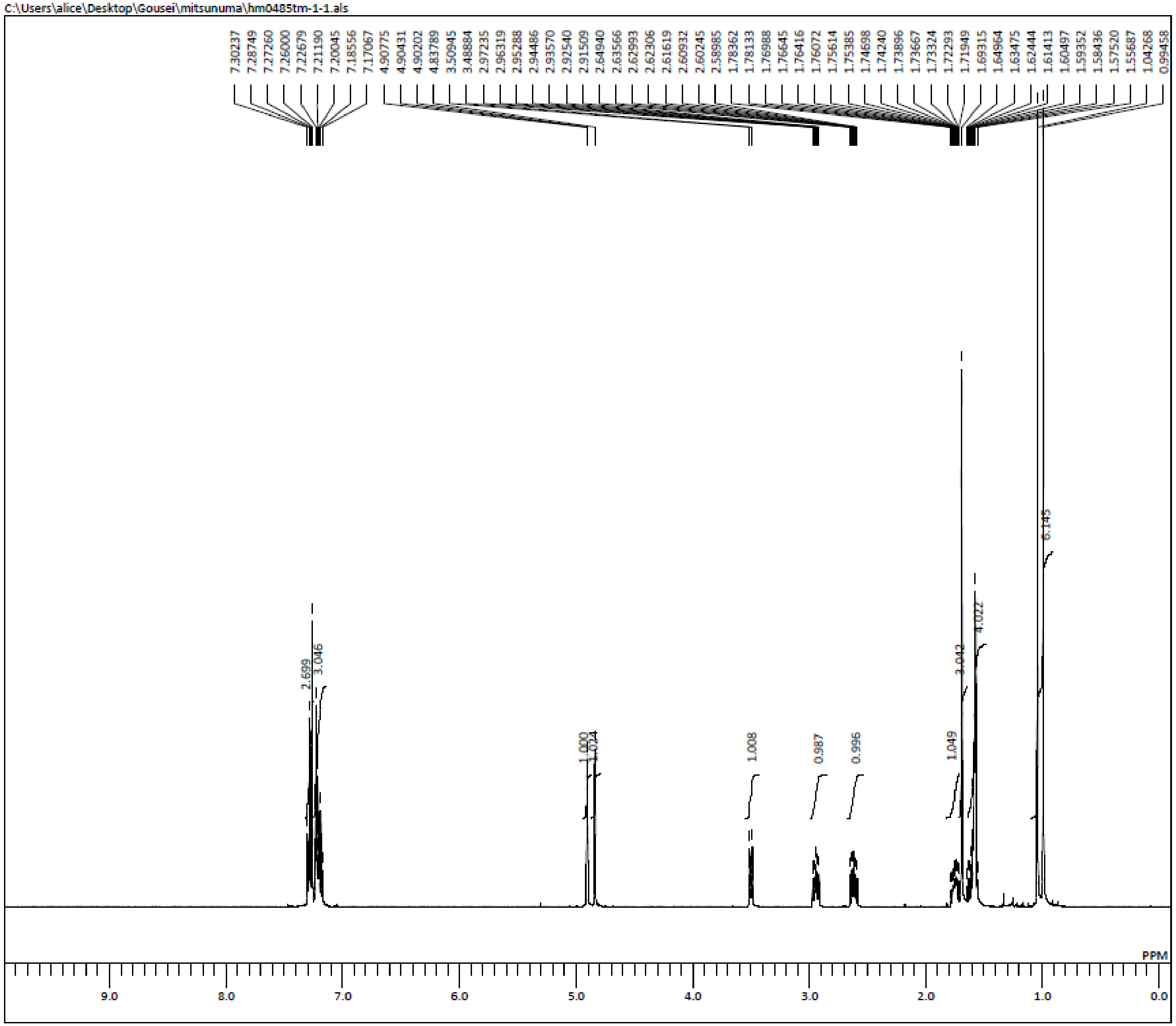

DFILE	hm0485tm-1-1.als
COMNT	
DATIM	2018-09-18 10:24:20
OBNUC	1H
EXMOD	proton.jxp
OBFRQ	500.16 MHz
OBSET	2.41 KHz
OBFIN	6.01 Hz
POINT	13107
FREQU	7507.51 Hz
SCANS	8
ACQTM	1.7459 sec
PD	5.0000 sec
PW1	5.55 usec
IRNUC	1H
CTEMP	21.9 c
SLVNT	CDCL3
EXREF	7.26 ppm
BF	0.12 Hz
RGAIN	32

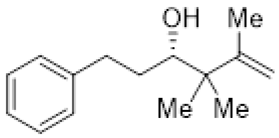

(*S*)-**8s**

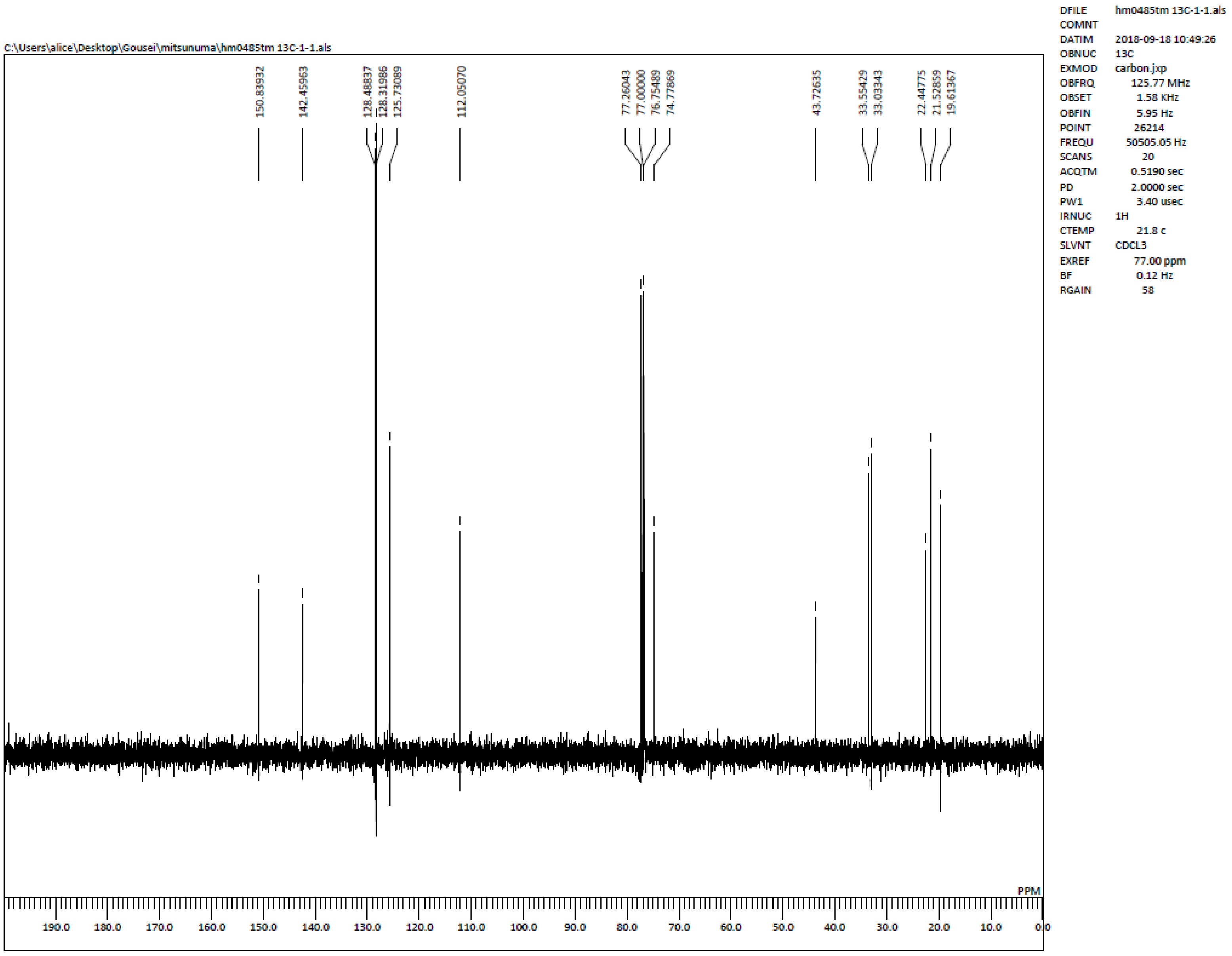
C:\Users\alice\Desktop\Gousei\mitsunuma\hm0485tm 13C-1-1.als
150.83932
142.45963
128.48837
128.31986
125.73089
112.05070
77.26043
77.00000
76.75489
74.77869
43.72635
33.55429
33.03343
22.44775
21.52859
19.61367
PPM
DFILE hm0485tm 13C-1-1.als
COMNT
DATIM 2018-09-18 10:49:26
OBNUC 13C
EXMOD carbon.jxp
OBFRQ 125.77 MHz
OBSET 1.58 KHz
OBFIN 5.95 Hz
POINT 26214
FREQU 50505.05 Hz
SCANS 20
ACQTM 0.5190 sec
PD 2.0000 sec
PW1 3.40 usec
IRNUC 1H
CTEMP 21.8 c
SLVNT CDCL3
EXREF 77.00 ppm
BF 0.12 Hz
RGAIN 58

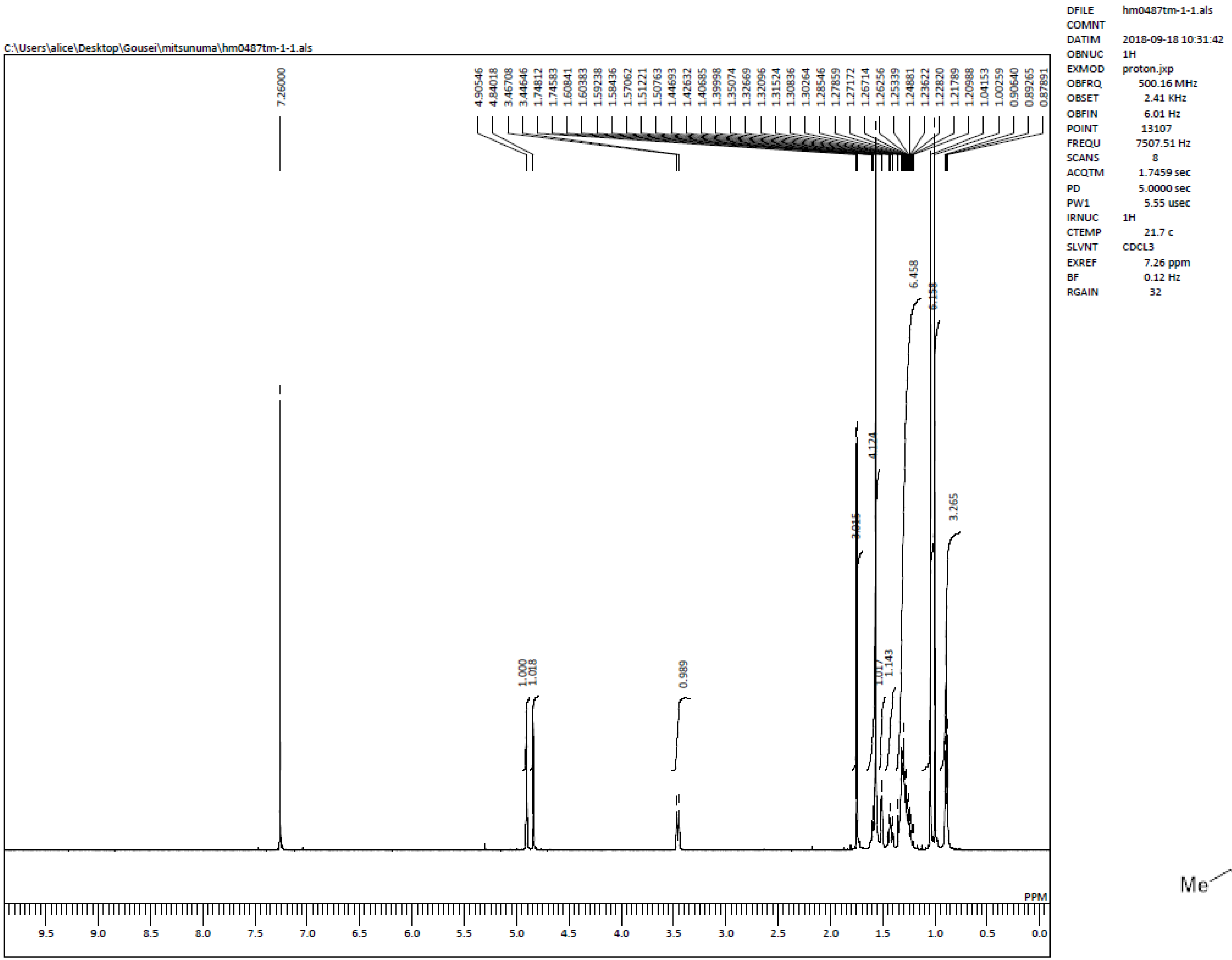
DFILE hm0487tm-1-1.als
COMNT
DATIM 2018-09-18 10:31:42
OBNUC 1H
EXMOD proton.jxp
OBFRQ 500.16 MHz
OBSET 2.41 KHz
OBFIN 6.01 Hz
POINT 13107
FREQU 7507.51 Hz
SCANS 8
ACQTM 1.7459 sec
PD 5.0000 sec
PW1 5.55 usec
IRNUC 1H
CTEMP 21.7 c
SLVNT CDCL3
EXREF 7.26 ppm
BF 0.12 Hz
RGAIN 32
C:\Users\alice\Desktop\Gousei\mitsunuma\hm0487tm-1-1.als
7.26000
4.90546
4.84018
3.46708
3.44646
1.74812
1.74583
1.60841
1.60383
1.59238
1.58436
1.57062
1.51221
1.50763
1.44693
1.42632
1.40685
1.39998
1.35074
1.32669
1.32096
1.31524
1.30836
1.30264
1.28546
1.27859
1.27172
1.26714
1.26256
1.25339
1.24881
1.23622
1.22820
1.21789
1.20988
1.04153
1.00259
0.90640
0.89265
0.87891
1.000
1.018
0.989
3.015
4.124
1.017
1.143
6.458
6.138
3.265
PPM
9.5
9.0
8.5
8.0
7.5
7.0
6.5
6.0
5.5
5.0
4.5
4.0
3.5
3.0
2.5
2.0
1.5
1.0
0.5
0.0

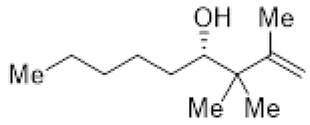
OH
Me
Me
Me
Me

(*S*)-**8t**

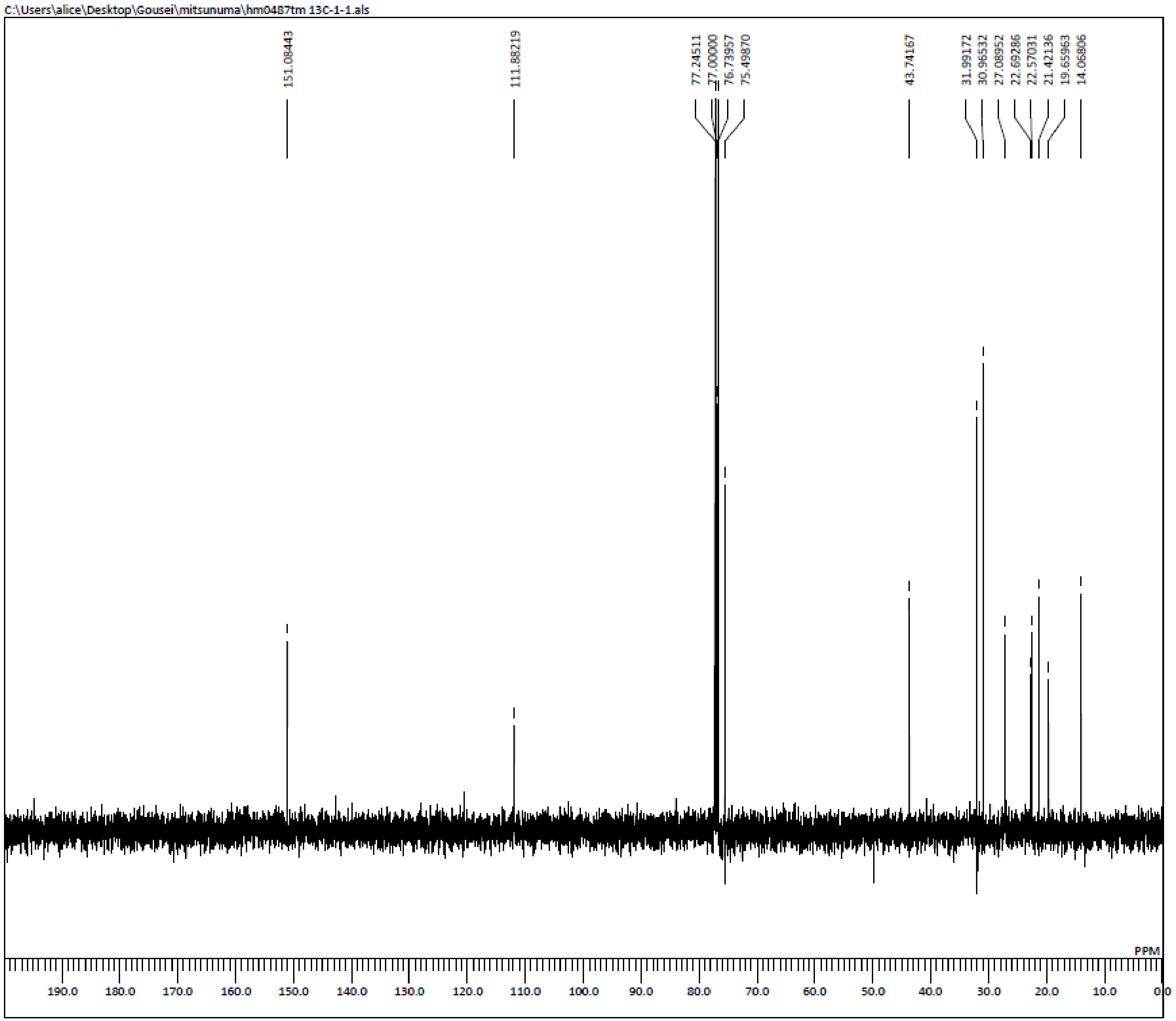

DFILE	hm0487tm 13C-1-1.als
COMNT	
DATIM	2018-09-18 10:55:58
OBNUC	13C
EXMOD	carbon.jxp
OBFRQ	125.77 MHz
OBSET	1.58 KHz
OBFIN	5.95 Hz
POINT	26214
FREQU	50505.05 Hz
SCANS	25
ACQTM	0.5190 sec
PD	2.0000 sec
PW1	3.40 usec
IRNUC	1H
CTEMP	21.6 c
SLVNT	CDCL3
EXREF	77.00 ppm
BF	0.12 Hz
RGAIN	60

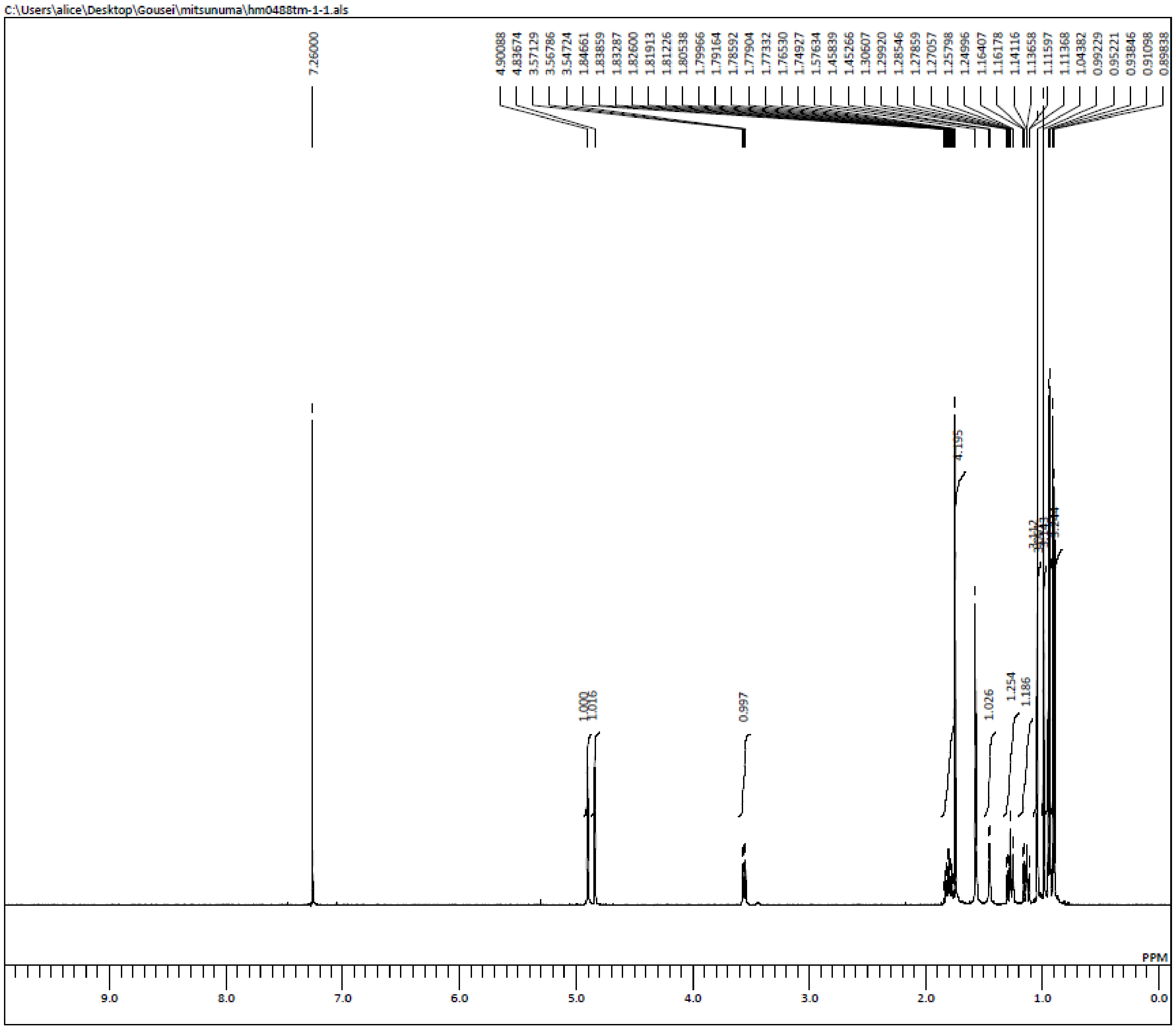

DFILE hm0488tm-1-1.als
COMNT
DATIM 2018-09-18 10:34:60
OBNUC 1H
EXMOD proton.jxp
OBFRQ 500.16 MHz
OBSET 2.41 KHz
OBFIN 6.01 Hz
POINT 13107
FREQU 7507.51 Hz
SCANS 8
ACQTM 1.7459 sec
PD 5.0000 sec
PW1 5.55 usec
IRNUC 1H
CTEMP 21.6 c
SLVNT CDCL3
EXREF 7.26 ppm
BF 0.12 Hz
RGAIN 32

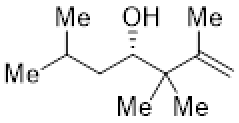

(*S*)-**8u**

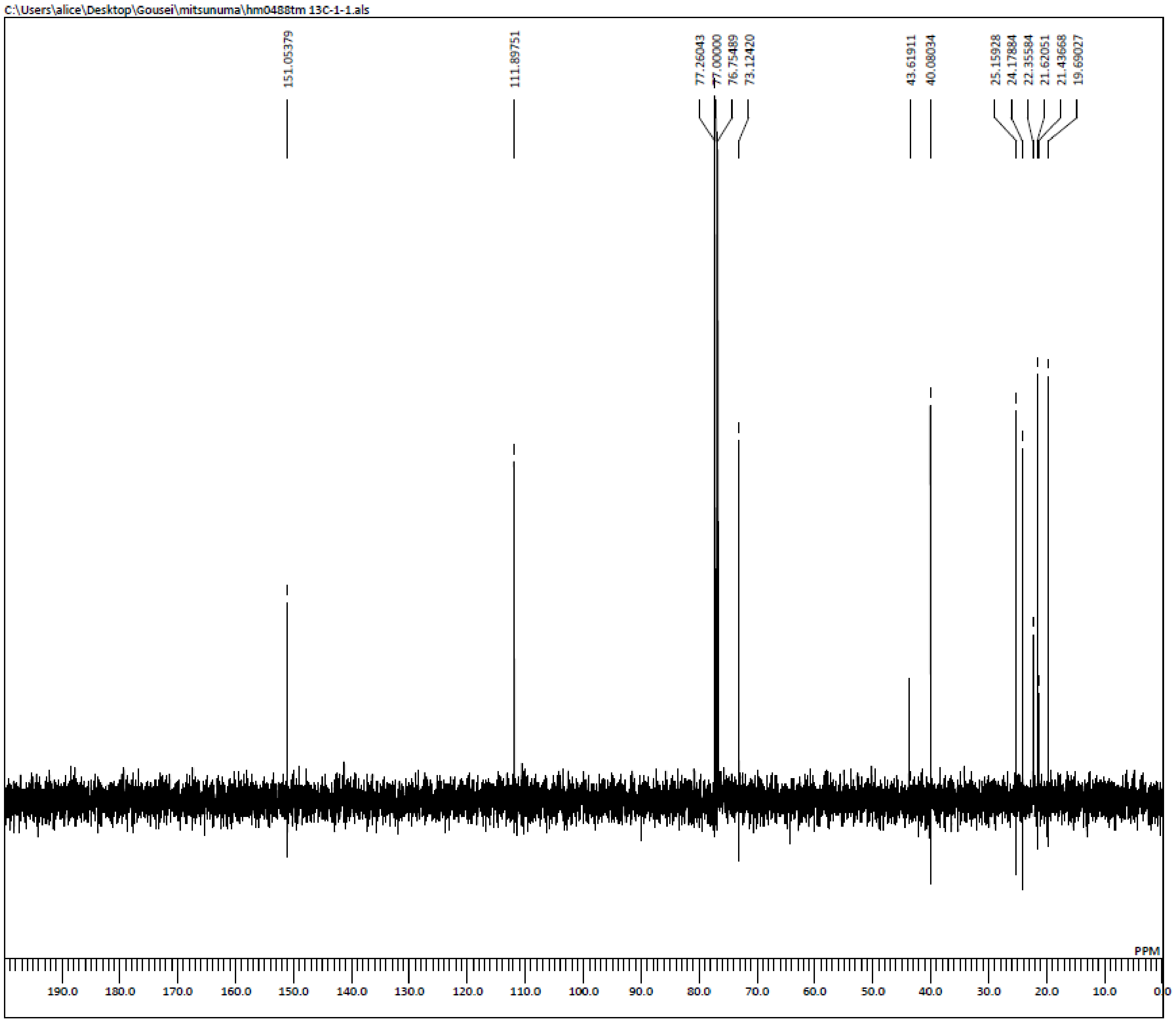

DFILE	hm0488tm 13C-1-1.als
COMNT	
DATIM	2018-09-18 10:58:48
OBNUC	13C
EXMOD	carbon.jxp
OBFRQ	125.77 MHz
OBSET	1.58 KHz
OBFIN	5.95 Hz
POINT	26214
FREQU	50505.05 Hz
SCANS	20
ACQTM	0.5190 sec
PD	2.0000 sec
PW1	3.40 usec
IRNUC	1H
CTEMP	21.3 c
SLVNT	CDCL3
EXREF	77.00 ppm
BF	0.12 Hz
RGAIN	60

H:\NMR\Mitsunuma\Allylation\ECS400data\st079product1-1H-1-1.als

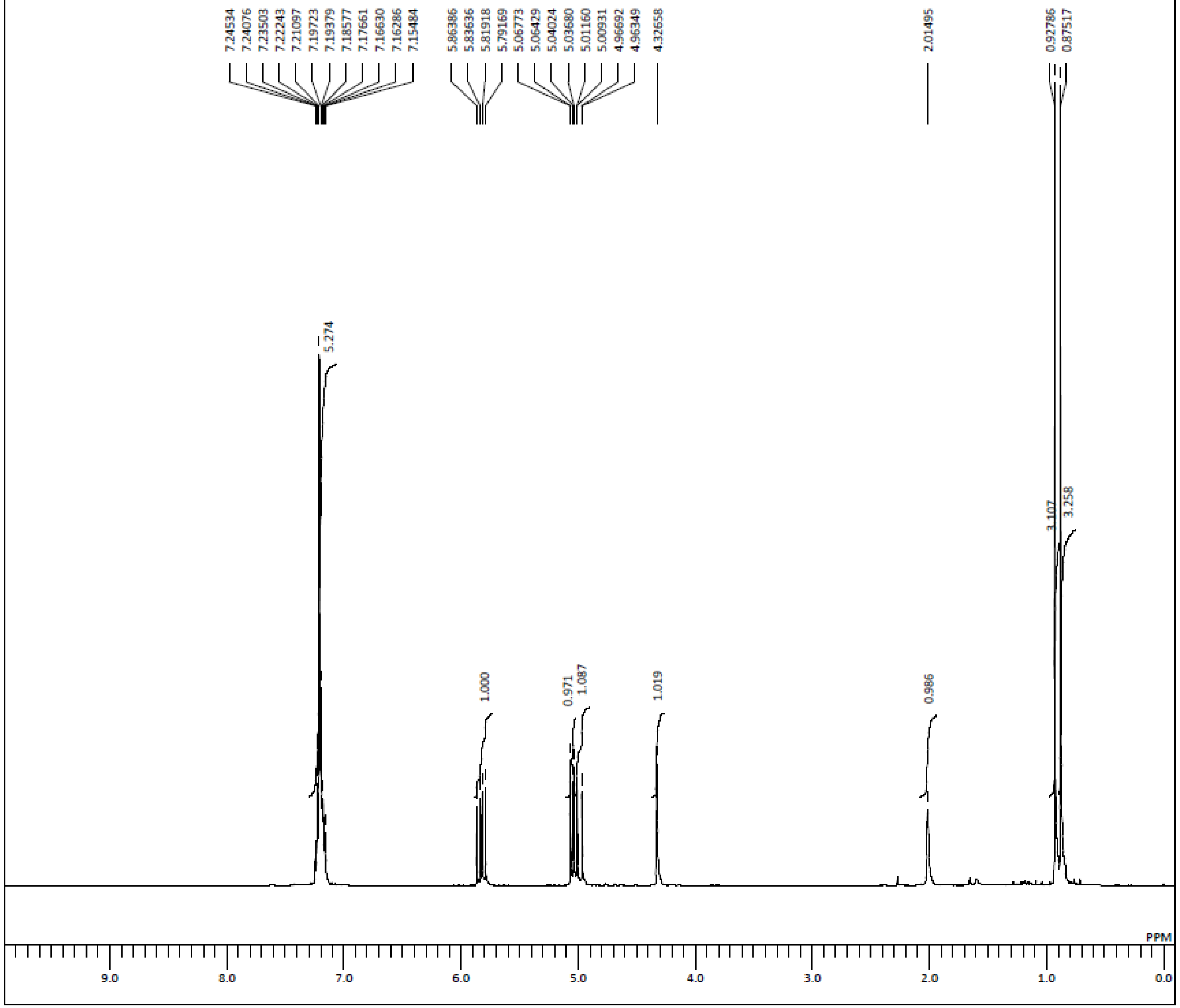

DFILE	st079product1-1H-1-1.als
COMNT	
DATIM	01-10-2018 21:37:08
OBNUC	1H
EXMOD	proton.jxp
OBFRQ	391.78 MHz
OBSET	8.51 KHz
OBFIN	3.34 Hz
POINT	13107
FREQU	5882.35 Hz
SCANS	8
ACQTM	2.2282 sec
PD	5.0000 sec
PW1	5.22 usec
IRNUC	1H
CTEMP	21.1 c
SLVNT	CDCL3
EXREF	0.00 ppm
BF	0.12 Hz
RGAIN	22

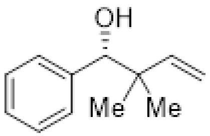

(*R*)-**8v**

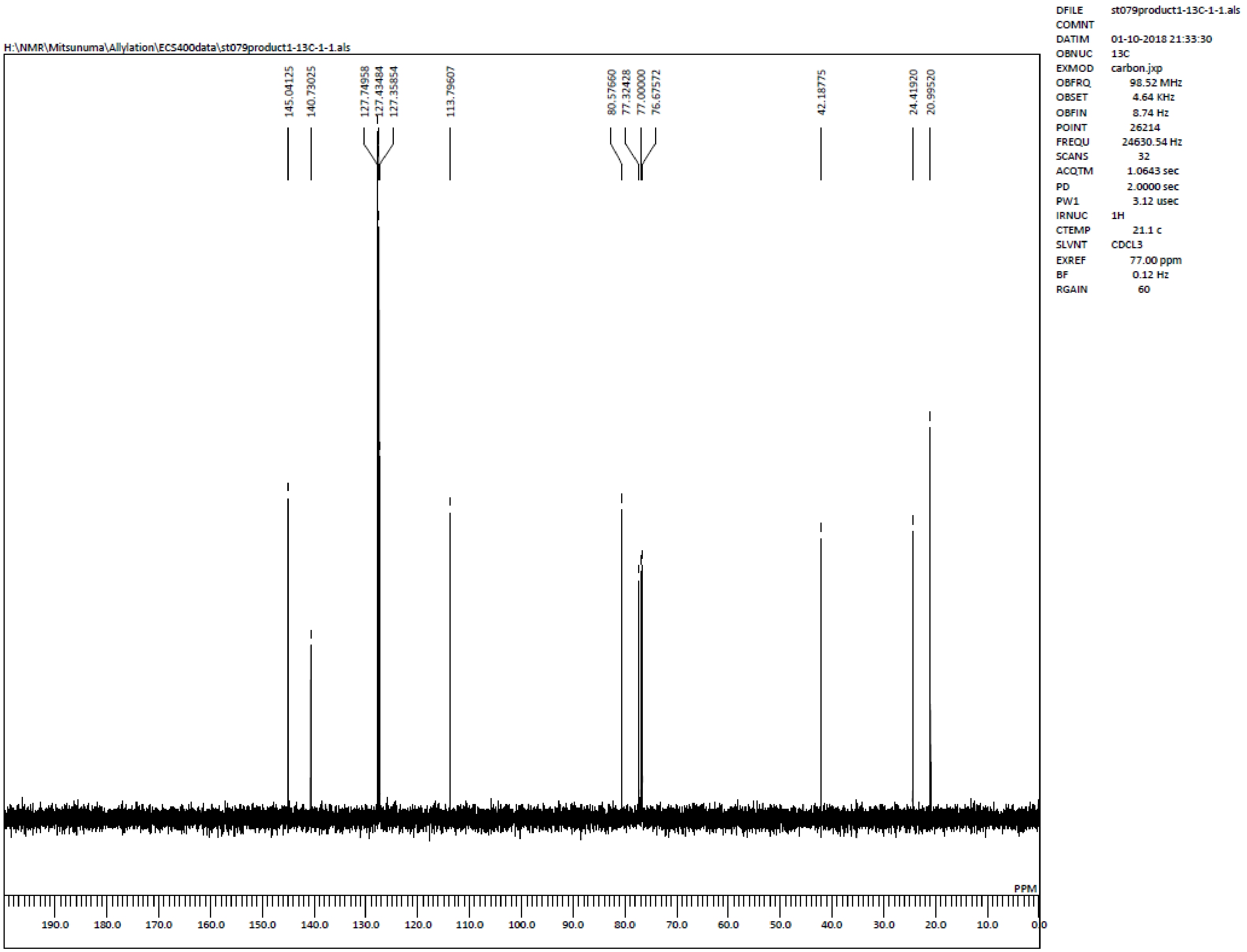
H:\NMR\Mitsunuma\Allylation\ECS400data\st079product1-13C-1-1.als
145.04125
140.73025
127.74958
127.43484
127.35854
113.79607
80.57660
77.32428
77.00000
76.67572
42.18775
24.41920
20.99520
PPM
190.0 180.0 170.0 160.0 150.0 140.0 130.0 120.0 110.0 100.0 90.0 80.0 70.0 60.0 50.0 40.0 30.0 20.0 10.0 0.0
DFILE st079product1-13C-1-1.als
COMNT
DATIM 01-10-2018 21:33:30
OBNUC 13C
EXMOD carbon.jxp
OBFRQ 98.52 MHz
OBSET 4.64 KHz
OBFIN 8.74 Hz
POINT 26214
FREQU 24630.54 Hz
SCANS 32
ACQTM 1.0643 sec
PD 2.0000 sec
PW1 3.12 usec
IRNUC 1H
CTEMP 21.1 c
SLVNT CDCL3
EXREF 77.00 ppm
BF 0.12 Hz
RGAIN 60
